# Biocatalytic production of adiponitrile and related aliphatic linear α,ω-dinitriles

**DOI:** 10.1038/s41467-018-07434-0

**Published:** 2018-11-30

**Authors:** Tobias Betke, Manuel Maier, Heidrun Gruber-Wölfler, Harald Gröger

**Affiliations:** 1 Chair of Organic Chemistry I, Faculty of Chemistry, Bielefeld University, Universitätsstrasse 25, 33615 Bielefeld, Germany; 2 Institute of Process and Particle Engineering, Graz University of Technology, Inffeldgasse 13/III, 8010 Graz, Austria

## Abstract

Linear α,ω-dinitriles are important precursors for the polymer industry. Most prominently, adiponitrile is produced on an annual scale of ca. 1 million tons. However, a drawback of today’s dominating process is the need for large amounts of highly toxic hydrogen cyanide. In this contribution, an alternative approach towards such linear dinitriles is presented based on dehydration of readily available α,ω-dialdoximes at ambient conditions by means of aldoxime dehydratases. In contrast to existing production routes this biocatalytic route enables a highly regio- and chemoselective approach towards dinitriles without the use of hydrogen cyanide or harsh reaction conditions. In addition, a selective synthesis of adiponitrile with substrate loadings of up to 100 g/L and high yields of up to 80% was achieved. Furthermore, a lab scale process on liter scale leading to > 99% conversion at 50 g/L underlines the potential and robustness of this method for technical applicability.

## Abstract (web summary)

Typically, preparation of the polymer precursors α,ω-dinitriles requires hydrogen cyanide. Here, the authors use aldoxime hydratase to produce adiponitrile and related aliphatic linear dinitriles under ambient conditions starting from readily available substrates without needing hydrogen cyanide.

## Introduction

**Fig. 1:**
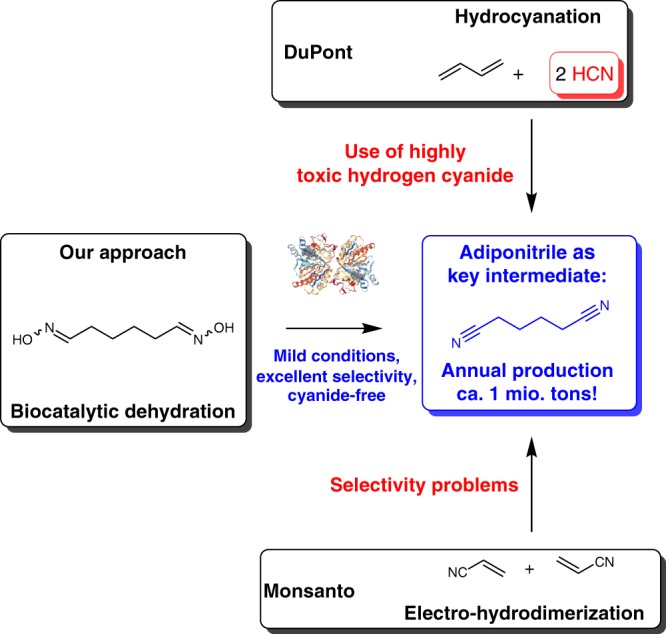
State-of-the-art production of adiponitrile and concept of this work. The hydrocyanation of butadiene and electro-hydrodimerization of acrylonitrile as established routes are shown in addition to the biocatalytic dehydration of α,ω-dialdoximes developed in this study

Nitriles are among the most important functional groups in chemistry and are of outstanding importance within the product tree of our today’s chemical industry^1,2^. Numerous product segments contain nitriles, ranging from pharmaceuticals and fine chemicals in the high price-low volume segment to the field of polymer building blocks and solvents as low price, but high volume chemicals. Besides acrylonitrile the most prominent example in terms of volume is adiponitrile being produced annually on ca. one million tons scale^2^. This compound is of high interest in the field of polymer chemistry due to its easy transformation into hexane-1, 6-diamine and later conversion into polyamides^1–8^. Earlier methods for the synthesis of adiponitrile are based on chlorine chemistry, however they lead to a significant amount of waste, thus making them less sustainable and—in the meantime—also less economical^2,9–11^. An alternative further production process has been developed by Monsanto and is based on an elegant coupling of acrylonitrile via electro-hydrodimerization^12^. However, a drawback is the limited selectivity. Thus, today’s dominant production process, which was developed by DuPont, is based on the use of butadiene in an addition reaction with hydrogen cyanide (Fig. 1)^13,14^. Although being efficient and successfully applied on large scale, high toxicity of hydrogen cyanide is a drawback of this production route as well as selectivity issues.

As a major current challenge in the field of the future’s chemicals product tree is to enable access to the existing bulk chemicals by changing the raw material basis as well as replacing hazardous methodologies and reagents by more environmentally benign processes, attempts have been made over many years and even decades in order to find improved production concepts also for aliphatic, linear dinitriles, in particular adiponitrile. Some of the newly discovered approaches for green chemistry-based nitrile synthesis (and especially for adiponitrile) are based on heterogeneous catalysts like non-noble metal oxides-based nanocatalysts^15^ or homogeneous catalysis, utilizing an iron nitrate/TEMPO system^16^. While these approaches are quite elegant avoiding the use of cyanides and starting from readily available alcohols (e.g., 1,6-hexanediol), some limitations exist. The heterogeneous approach suffers from high reaction temperatures ( ≥ 130 °C) and runs at elevated pressure of 5 bar of pure O_2_, thus raising safety issues. The homogeneous approach runs at mild reaction conditions but high catalyst loading (5 mol%) and tedious separation of the used iron nitrate and TEMPO are drawbacks. On the other hand, nature provides unique opportunities for organic synthesis. Thus, it is worth to identify natural approaches towards the preparation of specific functional groups and adapt them to chemical synthesis.

Here we report a biocatalytic production process of α,ω-dinitriles, which avoids the need of hydrogen cyanide and leads to the dinitriles with excellent selectivity by employing aldoxime dehydratases (Oxds) as in general no side-products were observed (Fig. 1). Towards this end, several α,ω-dialdoximes are synthesized and a substrate scope study with different Oxds is conducted. After identifying adipaldehyde dioxime as a highly suitable substrate for this biocatalytic transformation, a liter scale reaction with 50 g/L substrate loading is successfully conducted.

## Results and Discussion

### Process concept

**Fig. 2:**
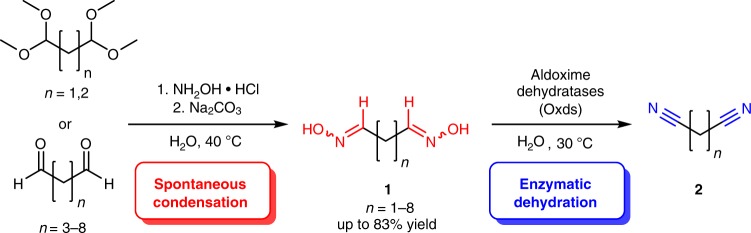
Sustainable chemoenzymatic approach towards α,ω-dinitriles starting from α,ω-dialdehydes or their bis-dimethyl acetals. Eight different α,ω-dialdoximes have been synthesized and investigated as substrates for the enzymatic dehydration by Oxds

This biocatalytic process can be conducted under mild reaction conditions and starts from a dialdoxime **1**, which is accessible via spontaneous condensation of an α,ω-dialdehyde raw material (in non-protected or acetal-protected form) with hydroxylamine. The dialdoxime **1** is then transformed into the desired dinitrile **2** via double dehydration in water utilizing an aldoxime dehydratase (Oxd) as a biocatalyst (Fig. 2). This enzyme class has just recently been discovered and introduced in the field of organic synthesis^17–26^.

However, as up to now, only monoaldoximes, including aromatic, heterocyclic, aryl-aliphatic, and linear aliphatic substrates, have been investigated as substrates for Oxd enzymes, the question arose if dialdoximes **1** are also recognized as substrates. In many cases of biocatalysis such a transformation of two moieties of the same functional group in one molecule turned out to be difficult or even impossible. A prominent example illustrating the difficulty of converting more than one of the same functional groups in a substrate is the enzymatic hydrolysis of diesters, in particular dialkyl malonates. In such a case only one of the two ester groups is hydrolyzed, which is in this case, however, useful and enables a desymmetrization reaction^27^.

### Substrate synthesis

The first research task was to synthesize the required dialdoxime substrates **1** (see Supplementary Fig. 27 to Fig. 42 and Supplementary Methods) for the biotransformations starting from the corresponding dialdehydes (see Supplementary Fig. 15 to Fig. 26 and Supplementary Methods) as most of the C3-C10 dialdoximes are rudimentarily described in literature. In general, a straightforward route towards aldehydes is based on hydroformylation, which represents a homogeneous catalytic process technology with one of the largest production volumes worldwide (exceeding 10 million tons per year)^2^. However, even nowadays no large-scale feasible approach for the double *n*-terminal hydroformylation of olefins is available. Thus, currently a lot of research effort is made towards the improvement of a double *n*-terminal hydroformylation of 1,3-butadiene in order to obtain adipaldehyde via such attractive access route^28–31^. Due to this (partial) restriction of the availability of the dialdehyde starting materials, we had to rely on alternative access routes towards them.

For the preparation of the C3- and C4-dialdoxime (**1a**,**b**) we started from the corresponding bis-dimethyl acetals. In case of the C5-dioxime (**1c**), direct synthesis from commercially available glutaraldehyde was possible. For the C6-dioxime (**1d**), however, we had to synthesize the dialdehyde via oxidation of *trans*−1,2-cyclohexanediol (for further details, see Supplementary Fig. 17 and 18 and Supplementary Methods). Lastly, access to the C7-C10 dioxime (**1e**-**h**) could be gained by oxidation of the corresponding dialcohols to their dialdehydes by utilization of a recently reported TEMPO-analogue^32^. In contrast to monoaldoximes, which are often obtained as oils or low melting solids by condensation of aldehydes with hydroxylamine sources, dialdoximes are high melting, amorphous solids. These dialdoximes **1** are highly stable compounds, which may be stored without any precautions for several months without deterioration. We were able to synthesize the dialdoximes of C3-C10 dialdehydes in moderate to high yields (see Supplementary Fig. 15 to Fig. 26 and Supplementary Methods). The synthesized dialdoximes **1** can be simply purified by filtration and drying in vacuo, yielding them with purities of up to ≥ 99%.

### Proof of the biotransformation process

With the synthesized substrates **1** in hand, as a next step we overexpressed five aldoxime dehydratases (Oxds) in *E.coli* and conducted a broad screening study with all five different whole-cell catalysts (used as resting cells for which cell lysis has not been observed) and the C3-C10 dialdoximes, including a co-solvent evaluation in order to identify suitable and stable biocatalysts for the desired biotransformations (for details, see Supplementary Fig. 1 to Supplementary Fig. 14 and Supplementary Methods). We were pleased to find that in particular the enzymes OxdA (from *Pseudomonas chlororaphis* B23) and OxdB (from *Bacillus* sp. OxB−1) turned out to be suitable for this purpose, showing a high remaining activity for at least 3 h when using 20% (v/v) of DMSO as a co-solvent in the dehydration of (*E*/*Z*)-phenylacetaldehyde oxime, which was used as a model substrate for studying the stability in this reaction medium. As a general technique for biotransformations with hardly soluble substrates in water, a co-solvent is often used for the initial substrate screening since it increases the solubility of substrates to avoid mass transfer limitations in the biotransformation.

**Fig. 3:**
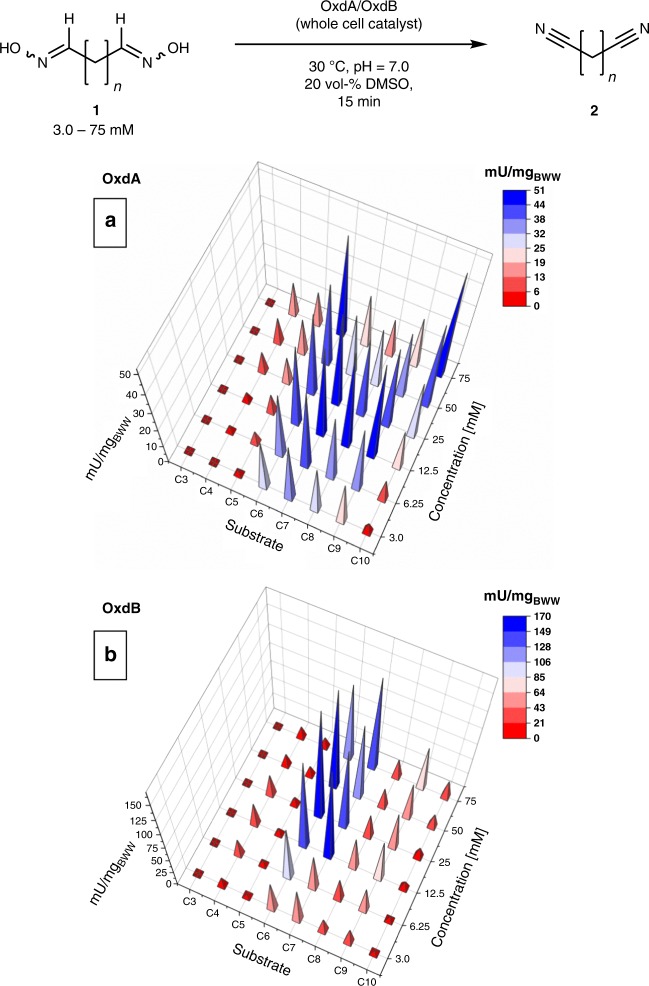
Substrate scope study for the biocatalytic dinitrile synthesis. **a** Activity values of OxdA in mU/mg_BWW_ for the C3-C10 dioximes. **b** Activity values of OxdB in mU/mg_BWW_ for the C3-C10 dioximes. BWW Bio wet weight; U-values calculated according to the conversion of one molecule of dialdoxime to one molecule of dinitrile

Based on the encouraging results in terms of stability when utilizing the OxdA and OxdB enzymes as whole-cell catalysts, we then conducted biotransformations on analytical scale with all eight dialdoxime substrates with a substrate concentration being in the range of 3.0–75 mM in order to get an insight into the impact of low substrate concentrations (being in part below the solubility limit) on the reaction course (Fig. 3).

The reported K_m_-values in the literature for linear, aliphatic monoaldoximes with chain-lenghts from C2-C6 range from 0.25–11.1 mM, thus being in a comparable range as the investigated substrate concentration. Surprisingly, we could observe a strong dependency on the chain-length of the substrate and its conversion by Oxd enzymes. While the C3-dialdoxime (**1a**) was not recognized by any Oxd enzyme as a substrate, in case of the C4- and C5-dialdoxime (**1b**,**c**) either no or a low conversion towards the dinitrile in combination with a strong tendency to accumulate an unkown intermediate was observed (especially on preparative scale experiments, data not shown). However, we were pleased to find that the C6-C10-dialdoximes (**1d**-**h**) were converted by both Oxds completely with excellent efficiency into the desired dinitriles. For most substrates the observed activities reached a maximum at substrate concentrations of 12.5 or 25 mM, leading to activity values of up to 169 U/mg_BWW_ (bww: bio wet weight) for the C6-dioxime when using the enzyme OxdB (for detailed activity values, see Supplementary Table 1). Although precipitation of the dialdoximes could be observed especially for the longer-chain substrates at higher concentrations, it is noteworthy that also in these cases there was mostly no strong negative impact on the enzyme activity.

**Fig. 4:**
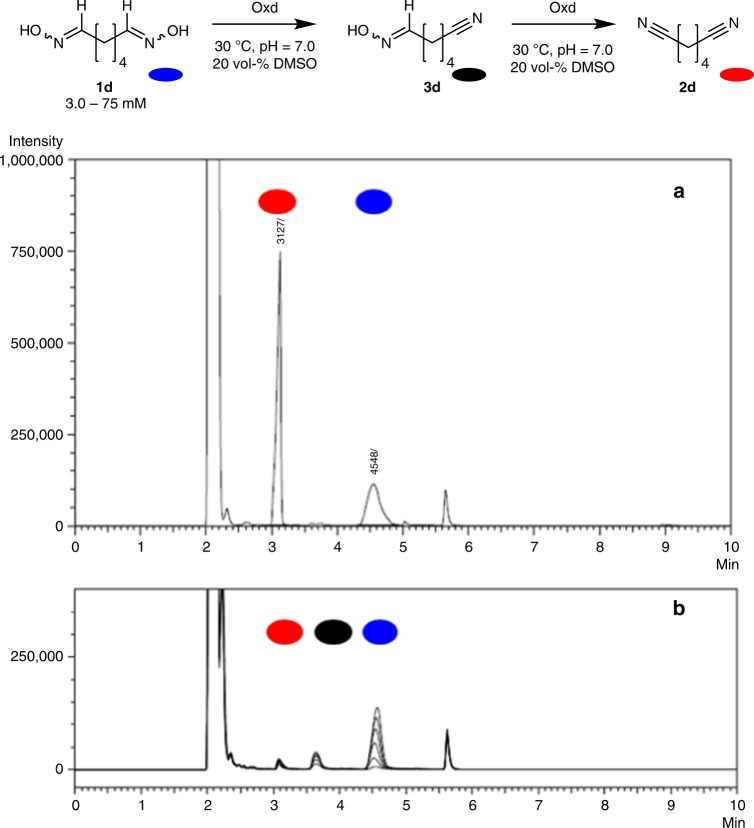
Postulated two-step dehydration of α,ω-dialdoximes to α,ω-dinitriles. **a** GC-chromatograms of a mixture of adiponitrile and adipaldehyde dioxime; **b** Overlay of GC-chromatograms illustrating the formation of the mononitrile-monooxime **3d** as an intermediate in the biocatalytic dehydration of dialdoxime **1d** using the aldoxime dehydratase OxdA (reaction time: 15 min)

In addition, we could gain an insight into the reaction course by means of GC analysis, observing intermediate signals in the corresponding GC-chromatograms. We postulate that they stem from the in situ formed monoaldoxime-mononitrile species **3** (here: **3d**) since the desired conversion of a dialdoxime **1** into the dinitrile **2** has to proceed via a two-step dehydration (as exemplified for the synthesis of adiponitrile **2d** from dialdoxime **1d** in the presence of the aldoxime dehydratase OxdA according to Fig. 4).

### Bioprocess development for adiponitrile synthesis

Based on the encouraging results, in particular for the synthesis of adiponitrile (**2d**), on analytical scale and due to its high industrial relevance, we decided to increase the scale as well as the substrate loading of the adiponitrile synthesis using both OxdA and OxdB as whole-cell catalysts (Table 1). Toward this end, we started the process development with initially 10 g/L substrate loading on a 100 mL scale with adipaldehyde aldoxime (**1d**). Importantly, we conducted the synthesis under an argon atmosphere since Oxds contain ferrous (Fe^II^) heme as a cofactor, which may be deactivated by oxidation to ferric (Fe^III^) heme^33–37^. The initial experiments with 10 g/L substrate loading proceeded smoothly under formation of adiponitrile (**2d**) with complete conversion, both for OxdA and OxdB enzymes (Table 1, entries 1 and 3).

**Table 1:** Preparative scale synthesis of adiponitrile with up to 100g/L substrate loading

^a^*BWW* Bio wet weight, *U* Unit, defined as µmol/min produced product

^b^100 mL reaction volume

^c^25 mL reaction volume

Since our activity studies on analytical scale revealed that active concentrations of less than 12.5 mM are sufficient for high activity of the Oxds, we decided to exclude the DMSO in comparative experiments to allow easier work-up after the biotransformation (Table 1, entries 2 and 4). To our delight, even in the absence of such an organic solvent we also obtained complete conversion after comparable reaction times (Table 1, entries 2 and 4). Since substrate **1d** is a solid and the product adiponitrile (**2d**) is a liquid, one can track the reaction process visually. After complete conversion towards adiponitrile (**2d**), the entire colorless solid has disappeared.

Since the overall activity of the enzyme OxdB towards adipaldehyde dioxime (**1d**) was higher compared to the one of the OxdA enzyme, we focused on the OxdB enzyme for further experiments with increased substrate loading. It is noteworthy that even increasing the substrate loading up to 50 g/L of **1d** (347 mM) led again to a complete conversion towards adiponitrile (entries 5 and 6). A further increase of the substrate loading to 100 g/L (694 mM) led to a maximum conversion of 70–75%, which was not improved even after adding additional whole-cell catalyst (entries 7 and 8). We suggest this may result from decreased enzyme stability under such elevated substrate and product loading conditions. It should be added that in all of these experiments selectivity was excellent and that no side-product formation was observed.

### Rationalizing the impact of reaction media

**Fig. 5:**
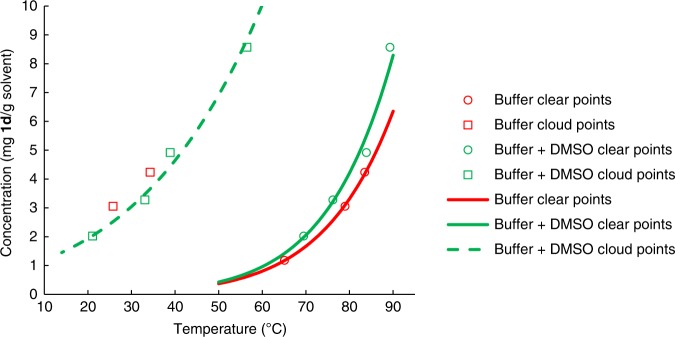
Determination of the solubility of adipaldehyde dioxime (**1d**). The solubility tests were conducted in presence or absence of DMSO in the reaction medium by heating the suspension containing **1d** and successive cooling of it

Taking into account the low substrate solubility, a surprising finding during the bioprocess development for adiponitrile synthesis was the lack of a positive impact of DMSO on the reaction (see Table 1, entries 1–6). Many literature-known biotransformations benefit from using water-miscible co-solvents to enhance the substrate solubility in water. As a hypothesis, we proposed that the solubility of the adipaldehyde dioxime (**1d**) had to be, while quite low, still sufficient in pure aqueous systems to reach a concentration being above the K_m_-value of the OxdB or at least in its K_m_-value range. Thus, a maximum velocity of the enzyme can be reached (or at least a velocity being not too far from its maximum). To proof this hypothesis and to rationalize the experimental finding of a negligible impact of the co-solvent DMSO, we investigated the solubility of adipaldehyde dioxime (**1d**) in 50 mM potassium phosphate buffer solution with and without DMSO (4:1 v/v). The obtained solubility data with these pure solutions (without biomass) indicate an unexpected high similarity of the solubility curves with and without DMSO as a co-solvent, thus being also in accordance with the experimental observation of similar conversions. The obtained data points were fitted with van’t Hoff type equations, whereby a good match was found (Fig. 5).

At the reaction temperature of 30 °C in both solvent systems the cloud points (at which crystallization of the substrate adipaldehyde dioxime occurs) are at ca. 3 g of adipaldehyde dioxime (**1d**) per Kg of aqueous solvent, whereas the clear points are at < 1 g of **1d** per Kg of solvent. Since in the experimental determination of the enzyme activity in dependency on the substrate concentration (according to Fig. 3) at 12.5 mM, which corresponds to an amount of 1.8 g/Kg, still a clear solution was observed (Supplementary Table 1), this indicates that also the presence of the biological material (whole-cells) might contribute to an improved solubility of the substrate compared to pure aqueous solutions. Furthermore, even such a low solubility of 12.5 mM of **1d** has been found to be sufficient for a high activity of the enzyme OxdB (as shown in Fig. 3).

### Liter scale synthesis of adiponitrile

**Fig. 6:**
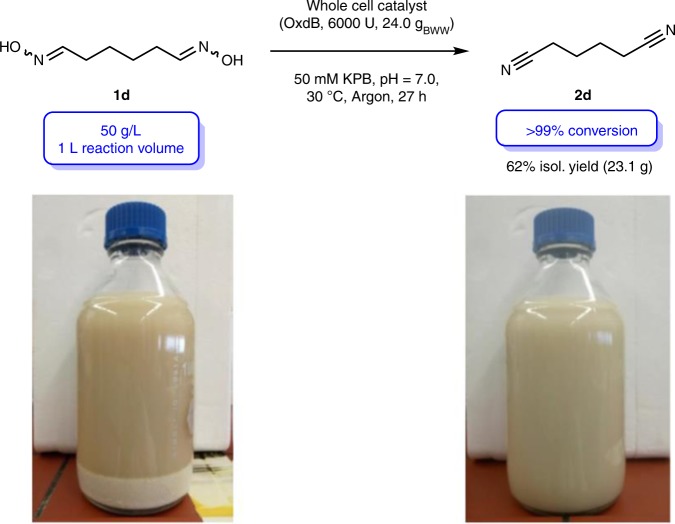
Synthesis of adiponitrile (**2d**) at a substrate loading of 50 g/L on liter scale. The photos show the reaction mixture at the start of the reaction and after a reaction time of 27 h, at which the solid substrate is completed converted

Encouraged by these successful biotransformations at elevated substrate loading, we decided to conduct the adiponitrile synthesis on a liter scale with a substrate loading of 50 g/L in pure aqueous reaction medium without the usage of DMSO (Fig. 6).

In this experiment the reaction proceeded again with excellent conversion of >99% and the colorless solid caused by the substrate has again disappeared after completion of the reaction (see also photos in Fig. 6). After work-up we obtained the adiponitrile (**2d**) in an isolated yield of 62% (23.1 g) and with a purity of > 98% (according to GC and ^1^H-NMR), demonstrating a high practicability, robustness, and scalability of the developed biocatalytic dinitrile synthesis via double aldoxime dehydration, thus underlining the potential of this type of Oxd enzyme technology for technical scale applications.

### A long-term envisioned green adipodinitrile production concept

**Fig. 7:**
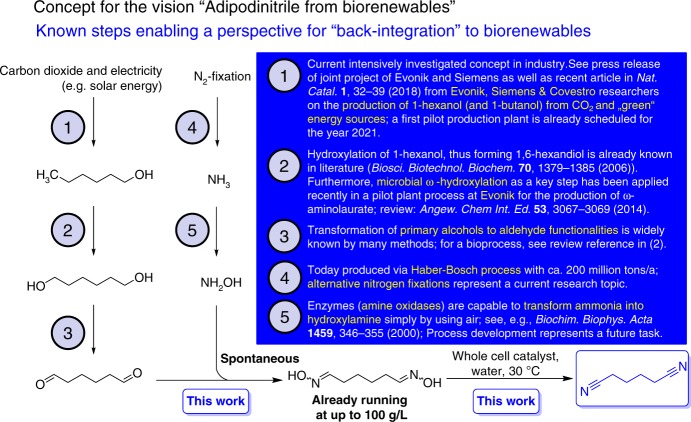
Overall concept for producing adiponitrile based on biorenewable feedstocks. Adipaldehyde may be synthesized starting from CO_2_ and solar energy in the future (step 1, 2, and 3) and the required hydroxylamine may be obtained by nitrogen fixation and subsequent selective oxidation of ammonia (step 4 and 5). Afterwards, the dioxime synthesis and biocatalytic dehydration would give access to adiponitrile based on completely renewable resources

Finally it should be added that a petrochemical approach based on the still challenging hydroformylation of butadiene is by far not the only industrially conceivable access to the C6-dialdehyde being needed as precursor for the biotransformation. There is even an overall process concept conceivable giving access to both raw materials (C6-dialdehyde and hydroxylamine) starting from exclusively renewable starting materials. Such a long-term vision, which is based on a sustainable as well as economically attractive access to both substrates for the C6-dialdoxime, namely the C6-dialdehyde and hydroxylamine, is summarized in Fig. 7.

With respect to such an alternative route to the C6-dialdehyde, the intermediate 1-hexanol is accessible from carbon dioxide and electricity in combination with electrochemistry and biotechnology. As very recently reported within a joint undertaking of an industry consortium consisting of Evonik AG, Siemens AG, and Covestro AG, such an approach delivers this compound^38–40^. A first pilot production is already planned for 2021, thus underlining the technical and scientific attractiveness of this concept^38,39^. In addition, ω-terminal hydroxylation^41^ by means of microbial processes is well known in general, as well as the transformation of 1-hexanol into 1,6-hexandiol^42^. For the subsequent transformation into the C6-dialdehyde, the use of oxoammonium salts^43^ was studied but also a biotechnological approach can be proposed in analogy to the successful formation of (functionalized) long chain alkyl-type aldehydes as demonstrated within the biocatalytic synthesis of an ω-aminolaurate even on pilot plant scale^41^. Since this C6-dialdehyde, however, is prone to side reactions and might be difficult to be isolated, direct in situ transformation to the C6-dialdoxime is an attractive option. The high stability of the C6-dialdoxime as well as its expected lower inhibition and deactivation of biocatalysts compared to the (C6-)dialdehyde makes it a more stable and attractive intermediate. This option to circumvent accumulation and isolation of the sensitive C6-dialdehyde by in situ-conversion to the C6-dialdoxime could make the route towards 1,6-hexanediamine via adiponitrile and subsequent hydrogenation more preferred compared to a direct reductive amination of the C6-dialdehyde. Furthermore, also for the second reagent, hydroxylamine, being utilized today as a commodity chemical for the multi-million tons scale production of ε-caprolactam^2^, an alternative option for a large-scale access exists. Since hydroxylamine is a metabolite in nature and since the enzyme for ammonia hydroxylation is already disclosed one can start from ammonia and just by means of air and an amine oxidase then hydroxylamine is formed^44^. Thus, not necessarily the current technical route might be the only conceivable approach in the future and in addition to the efficient biotransformation utilizing aldoxime dehydratases as described above a general way to adiponitrile from fully renewable raw materials exist with all individual steps—except for the reported biotransformation as the missing link—being described already in literature.

In conclusion, to the best of our knowledge this process concept starting from the corresponding dialdoximes represents a general access to adiponitrile and other dinitriles by means of enzyme catalysis. A broad substrate scope was demonstrated and an efficient bioprocess on liter scale was developed enabling the production of adiponitrile with >99% conversion at a substrate loading of 50 g/L. For the future we will focus on further bioprocess development, as well as on an efficient production of the raw materials. As for the bioprocess, recycling of the biocatalyst and conducting the dinitrile synthesis in other reaction media like organic media are remaining challenges. As for the raw material issue, in addition to the conceptual approach starting from biorenewable resources the utilization of chemocatalytic hydroformylation can be considered, which is known as a highly efficient methodology for aldehyde synthesis. Currently, a lot of research effort is made towards the improvement of a double *n*-terminal hydroformylation of 1,3-butadiene in order to obtain adipaldehyde with favorable production data^28–31^. Together with an economical dialdehyde synthesis, the described Oxd enzyme-based technology then would enable a safe, robust, and attractive chemoenzymatic production route towards aliphatic linear α,ω-dinitriles, e.g., adiponitrile.

## Methods

### Materials

The compounds *trans*−1,2-cyclohexanediol, sodium periodate, glutaraldehyde (50 wt% in water), malonaldehyde bis(dimethyl acetal), succinaldehyde bis(dimethyl acetal), Bobbitt’s salt, hydroxylamine hydrochloride, sodium carbonate, 1,7-heptanediol, 1,8-octanediol, 1,9-nonanediol, 1,10-decanediol for the synthesis of substrates, and all dinitriles for reference purposes were purchased commercially and used as received.

### Enzyme preparation

Recombinant expression of the aldoxime dehydratase from *Pseudomonas chlororaphis* B23 (OxdA), aldoxime dehydratase from *Bacillus* sp. OxB−1 (OxdB), aldoxime dehydratase from *Fusarium graminearum* MAFF305135 (OxdFG), aldoxime dehydratase from *Rhodococcus* sp. N−771 (OxdRE) and aldoxime dehydratase from *Rhodococcus globerulus* A−4 (OxdRG) was conducted as described in the Supplementary Methods, which contains a detailed protocol for the whole-cell catalyst preparation. A description of the structures of plasmids encoding the Oxd enzyes as well as expression data (SDS-PAGE) are given in the Supplementary Fig. 1 to Fig. 5 and Fig. 6, respectively, and Supplementary Table 2. The enzyme activities of these Oxd are summarized in Supplementary Table 1.

### Screening of Oxd enzymes

The protocol of these screening experiments and analytical data of the resulting dinitrile products are given in the Supplementary Methods and Supplementary Fig. 43 to 52.

### Procedure for the biocatalytic synthesis of adiponitrile

A 100 mL reaction mixture consisting of whole-cell catalyst suspension in 50 mM KPB, pH = 7.0 (0.6–4.0 wt% BWW, containing OxdA or OxdB) and the solid adipaldehyde dioxime (**1d**, 1.0–10 g) were mixed in a sealable glass flask. Argon was flushed through the flask and it was sealed afterwards. The mixture was stirred at 180 rpm at 30 °C. In case of using DMSO as a co-solvent, the reaction mixture consisted of 80 mL cell suspension and 20 mL DMSO. An aliquot of 1.0 mL was taken several times to determine the conversion via gas chromatography (GC). For this, the aliquot was mixed with 1.0 mL 2-Me-THF and extracted for 1 min. The supernatant was taken off und injected into the GC apparatus. The conversion was determined according to calibration curves. After complete conversion to adiponitrile, the reaction mixture was extracted three times with MTBE (1:1, v/v). In case of using DMSO as co-solvent, the combined extracts were washed once with brine (1:3, v/v). Subsequently, the extracts were dried over MgSO_4_, filtered and the solvent was removed in vacuo to yield adiponitrile (**2d**) as pale yellow liquid with 98–99% purity. The purity of the product was determined via ^1^H-NMR- and GC analysis. For more details, see Supplementary Methods and Supplementary Table 3.

### Determination of solubility

Solubility measurements were carried out with the Crystal16 from Technobis group, Netherlands. The measurement principle of this device is based on transmissivity detection through a defined amount of sample while varying the temperature to find clear points ( = solubility limit) and cloud ( = spontaneous crystallization) points. This device utilizes 16 sample ports for 2.5 ml HPLC vials each which are arranged in 4 times 4 blocks. In each block, rpm, and temperature can be set independently to the other blocks. The protocol for the solubility measurement used in each block started by setting the ports to 20 °C, followed by a hold and placement of blank vials with only solvent for a transmissivity tuning step. Next, the samples were inserted and while stirring with magnets at 600 rpm, the samples were heated to 90 °C and then cooled to 0 °C with 0.5 °C per minute. After a hold of 30 min at 0 °C, the samples were again brought to 90 °C to obtain a second solubility point for each sample, which was then used together with the first point for averaging.

## Electronic supplementary material

Supplementary Material

Reporting Summary

## Data Availability

Data supporting the findings of this study are available within this article and its related Supplementary Information or are available from the corresponding author upon reasonable request.
