# Supplementary material for: Biocatalytic production of adiponitrile and related aliphatic linear α,ω-dinitriles
Source: Nat Commun. 2018 Nov 30;9:5112. doi: 10.1038/s41467-018-07434-0 (PMC6269433; doi:10.1038/s41467-018-07434-0)
Supplement: Supplementary file 1 — Supplementary Material [file 41467_2018_7434_MOESM1_ESM.pdf]

Supplementary Information

Biocatalytic production of adiponitrile and related aliphatic linear α,ω-dinitriles

Betke *et al.*

Supplementary Figures

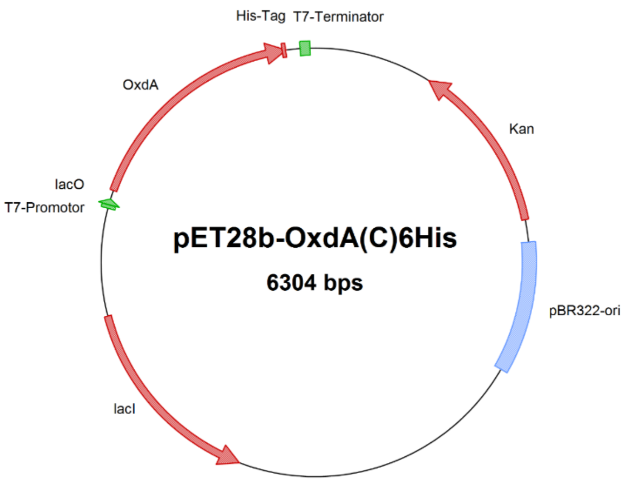

Supplementary Figure 1. Structure of the plasmid encoding for OxdA.

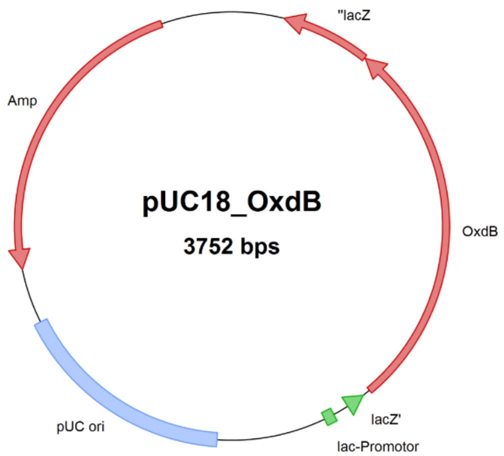

Supplementary Figure 2. Structure of the plasmid encoding for OxdB.

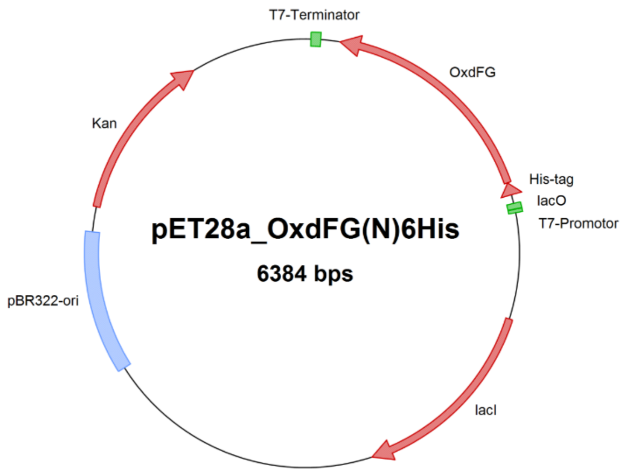

Supplementary Figure 3. Structure of the plasmid encoding for OxdFG.

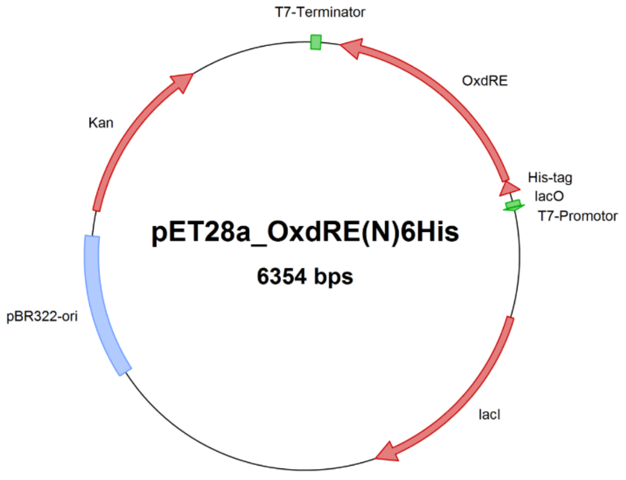

Supplementary Figure 4. Structure of the plasmid encoding for OxdRE.

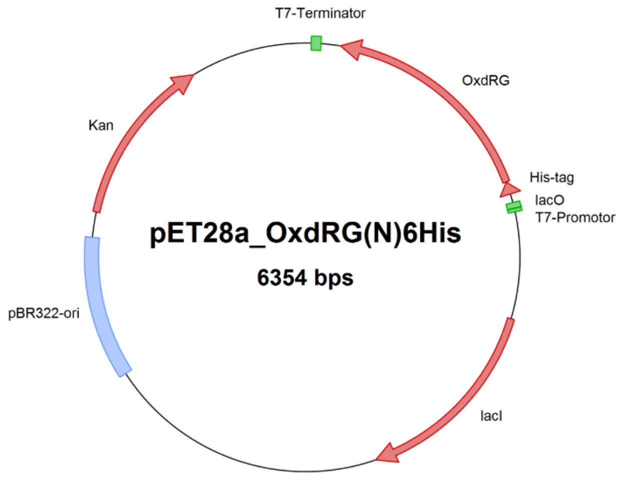

Supplementary Figure 5. Structure of the plasmid encoding for OxdRG.

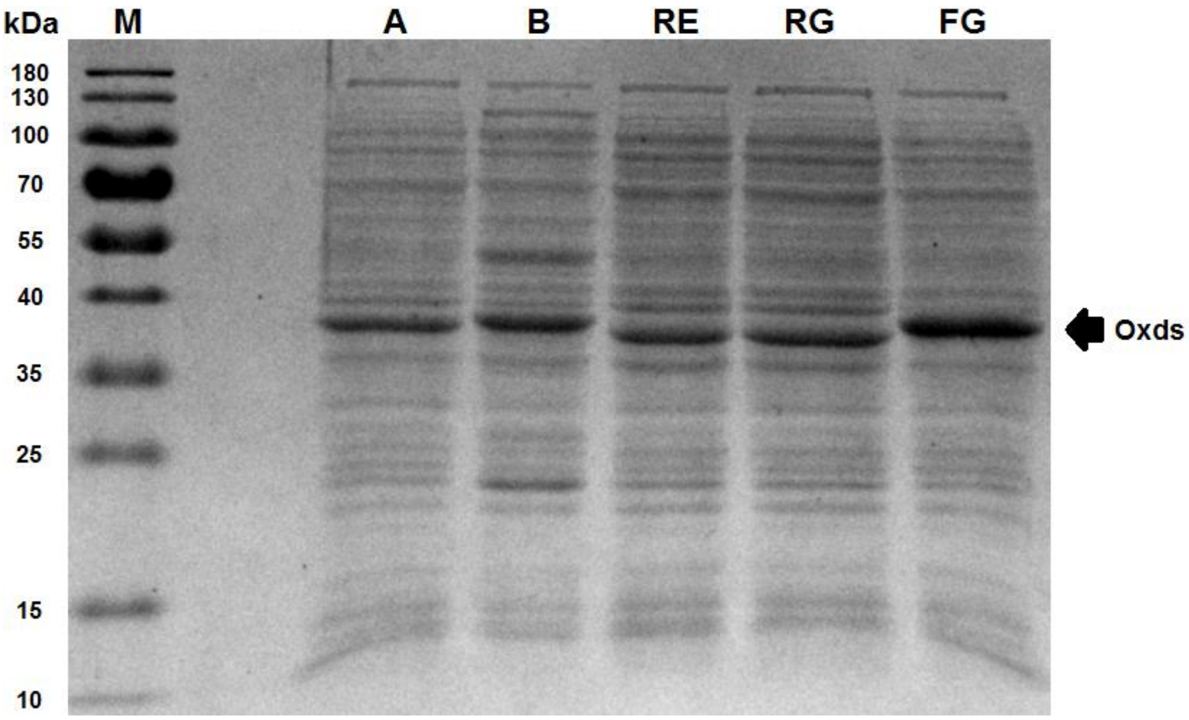

Supplementary Figure 6. SDS-PAGE of all five crude extracts for OxdA (A), OxdB (B), OxdFG (FG), OxdRE (RE), OxdRG (RG). The molecular weight of the Oxds is in good agreement with the literature data.[1–7]

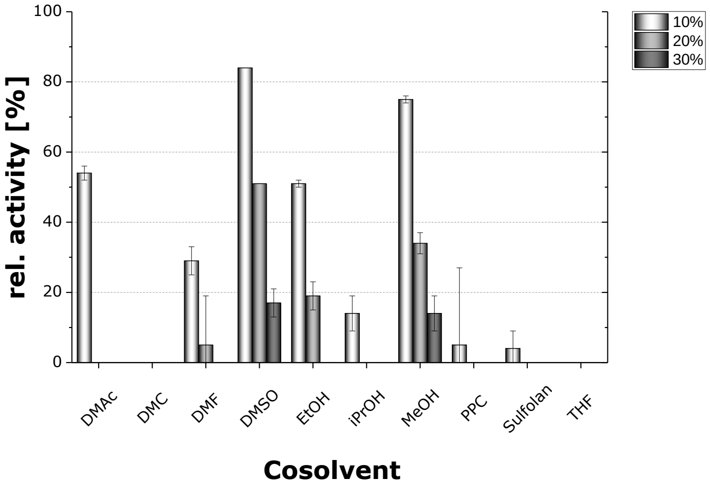

Supplementary Figure 7. Relative activity of OxdA(C) in presence of water soluble co-solvents (for different volumetric percentages). The whole-cell suspension was incubated for 20 minutes with the co-solvent prior to the activity assay. Each experiment was conducted in duplicate. The relative activity was determined by comparison with a reference experiment, in which no co-solvent was added during the incubation time. Error bars represent the standard error of the duplicate HPLC measurements.

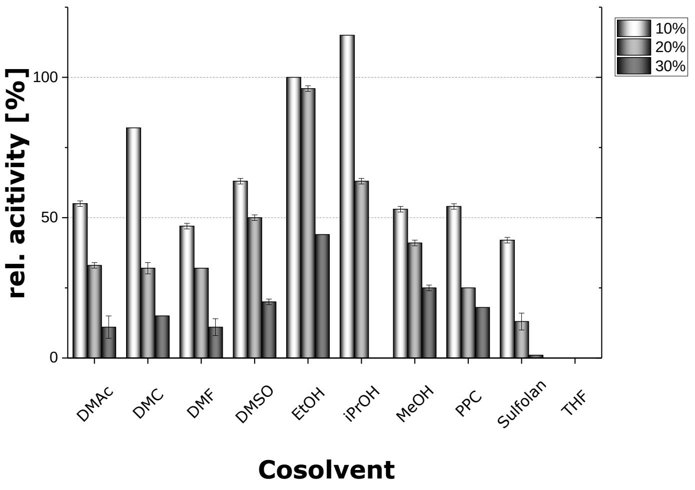

Supplementary Figure 8. Relative activity of OxdB in presence of water soluble co-solvents (for different volumetric percentages). The whole-cell suspension was incubated for 20 minutes with the co-solvent prior to the activity assay. Each experiment was conducted in duplicate. The relative activity was determined by comparison with a reference experiment, in which no co-solvent was added during the incubation time. Error bars represent the standard error of the duplicate HPLC measurements.

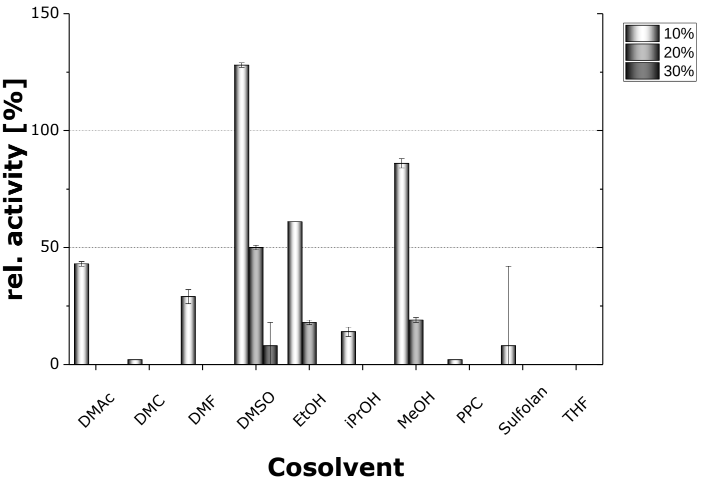

Supplementary Figure 9. Relative activity of OxdFG(N) in presence of water soluble co-solvents (for different volumetric percentages). The whole-cell suspension was incubated for 20 minutes with the co-solvent prior to the activity assay. Each experiment was conducted in duplicate. The relative activity was determined by comparison with a reference experiment, in which no co-solvent was added during the incubation time. Error bars represent the standard error of the duplicate HPLC measurements.

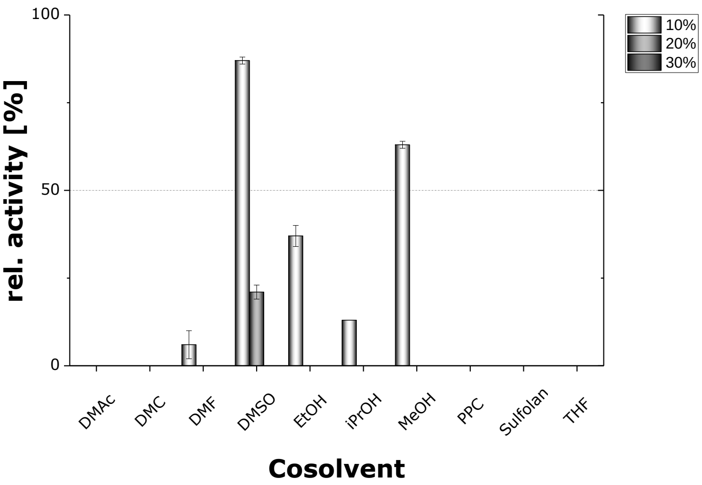

Supplementary Figure 10. Relative activity of OxdRE(N) in presence of water soluble co-solvents (for different volumetric percentages). The whole-cell suspension was incubated for 20 minutes with the co-solvent prior to the activity assay. Each experiment was conducted in duplicate. The relative activity was determined by comparison with a reference experiment, in which no co-solvent was added during the incubation time. Error bars represent the standard error of the duplicate HPLC measurements.

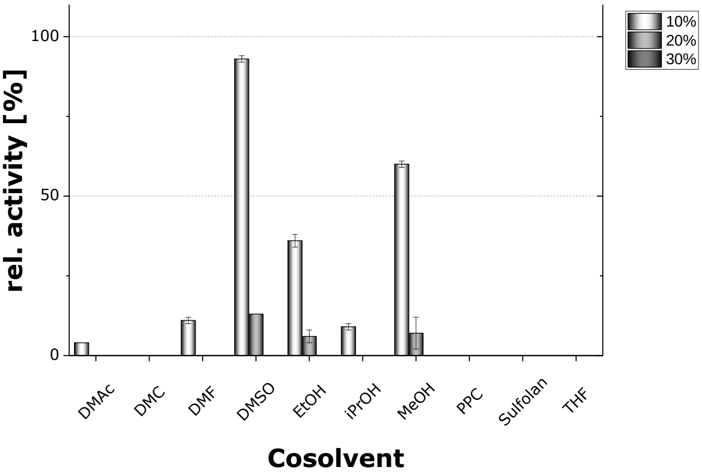

Supplementary Figure 11. Relative activity of OxdRG(N) in presence of water soluble co-solvents (for different volumetric percentages). The whole-cell suspension was incubated for 20 minutes with the co-solvent prior to the activity assay. Each experiment was conducted in duplicate. The relative activity was determined by comparison with a reference experiment, in which no co-solvent was added during the incubation time. Error bars represent the standard error of the duplicate HPLC measurements.

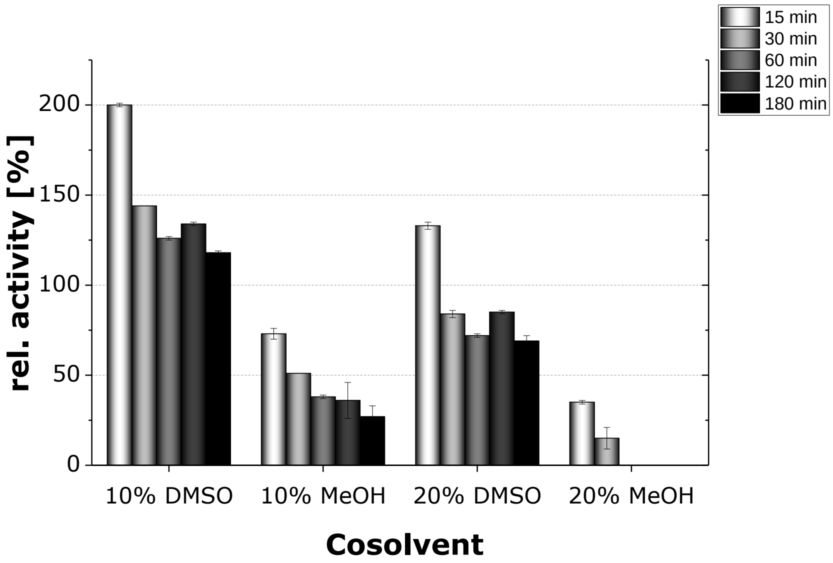

Supplementary Figure 12. Long-term stability study for OxdA(C). The whole-cell suspension was incubated for 15, 30, 60, 120 and 180 minutes with a co-solvent prior to the activity assay. Each experiment was conducted in duplicate. The relative activity was determined by comparison with a reference experiment, in which no co-solvent was added during the incubation time. Error bars represent the standard error of the duplicate HPLC measurements.

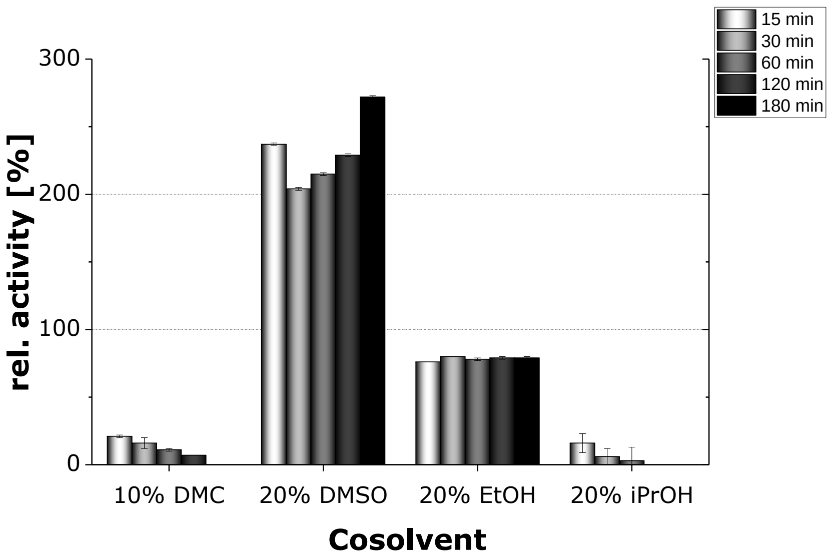

Supplementary Figure 13. Long-term stability study for OxdB. The whole-cell suspension was incubated for 15, 30, 60, 120 and 180 minutes with a co-solvent prior to the activity assay. Each experiment was conducted in duplicate. The relative activity was determined by comparison with a reference experiment, in which no co-solvent was added during the incubation time. Error bars represent the standard error of the duplicate HPLC measurements.

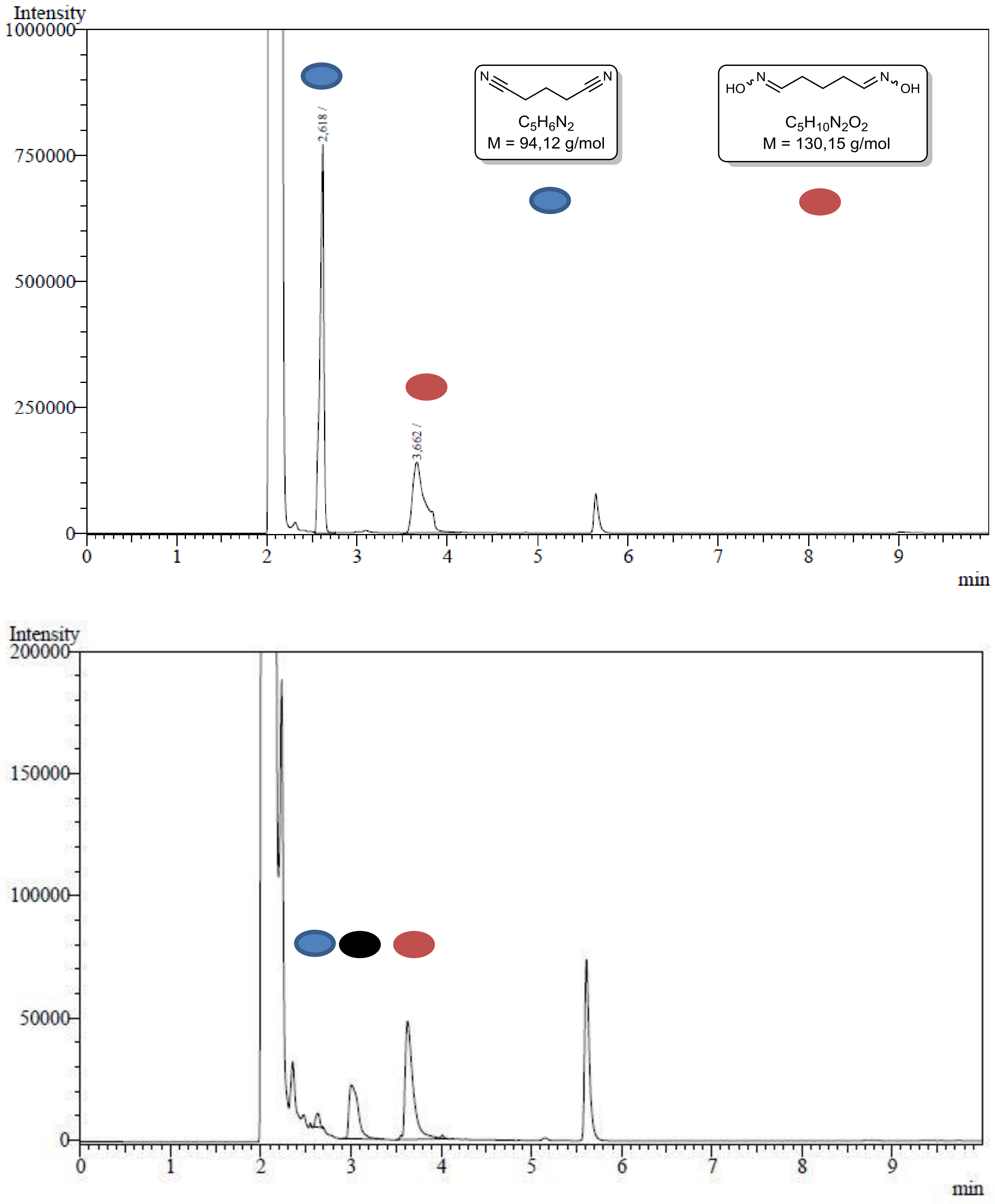

Supplementary Figure 14. GC chromatograms of a reference mixture of the C5 dinitrile and dioxime and a chromatogram of a preparative biotransformation after one day.

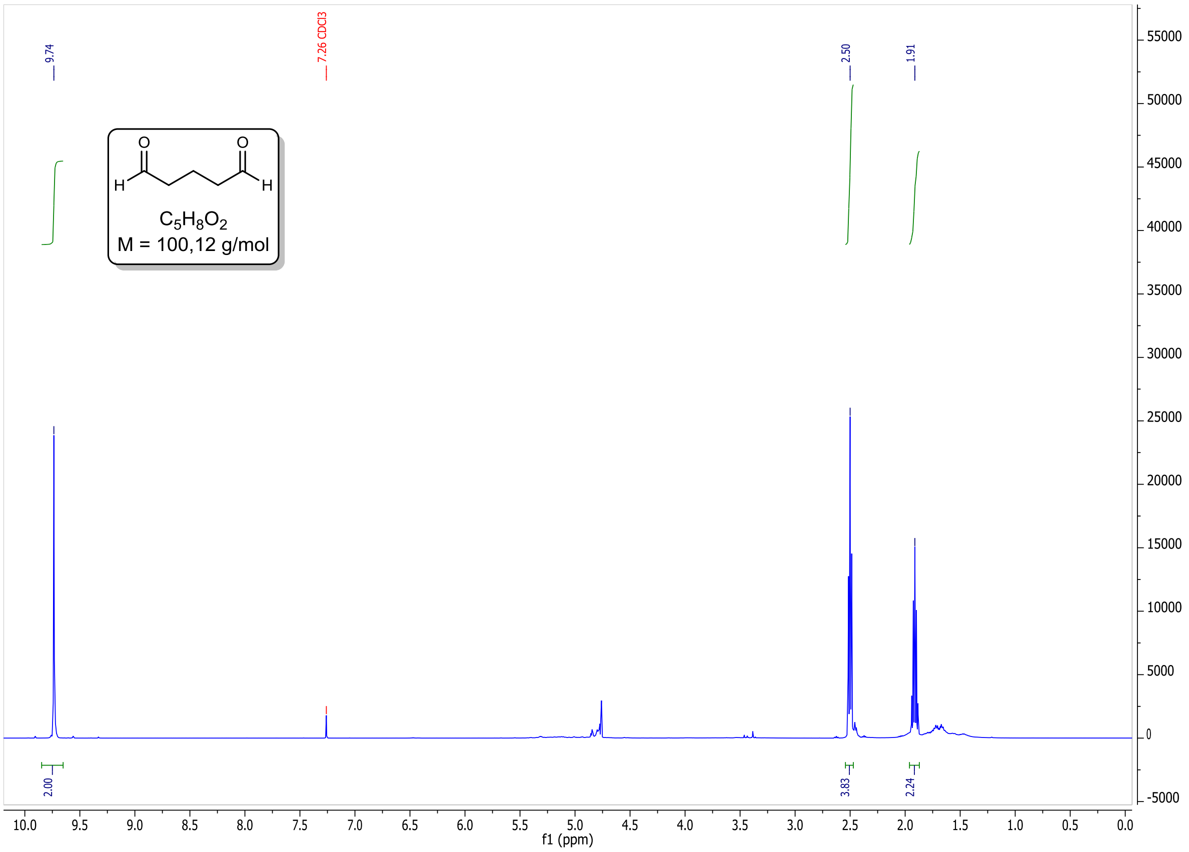

Supplementary Figure 15. ^{1}H-NMR spectra of glutaraldehyde.

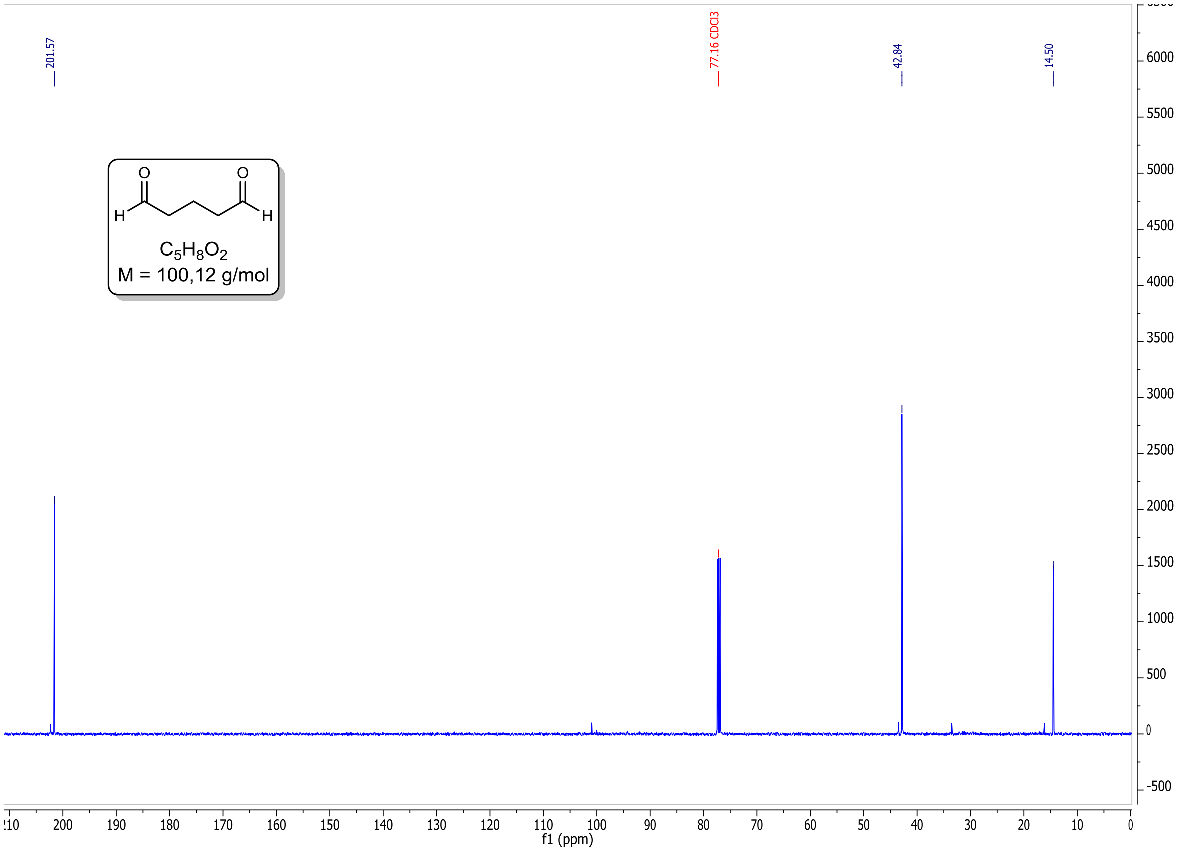

Supplementary Figure 16. ^{13}C-NMR spectra of glutaraldehyde.

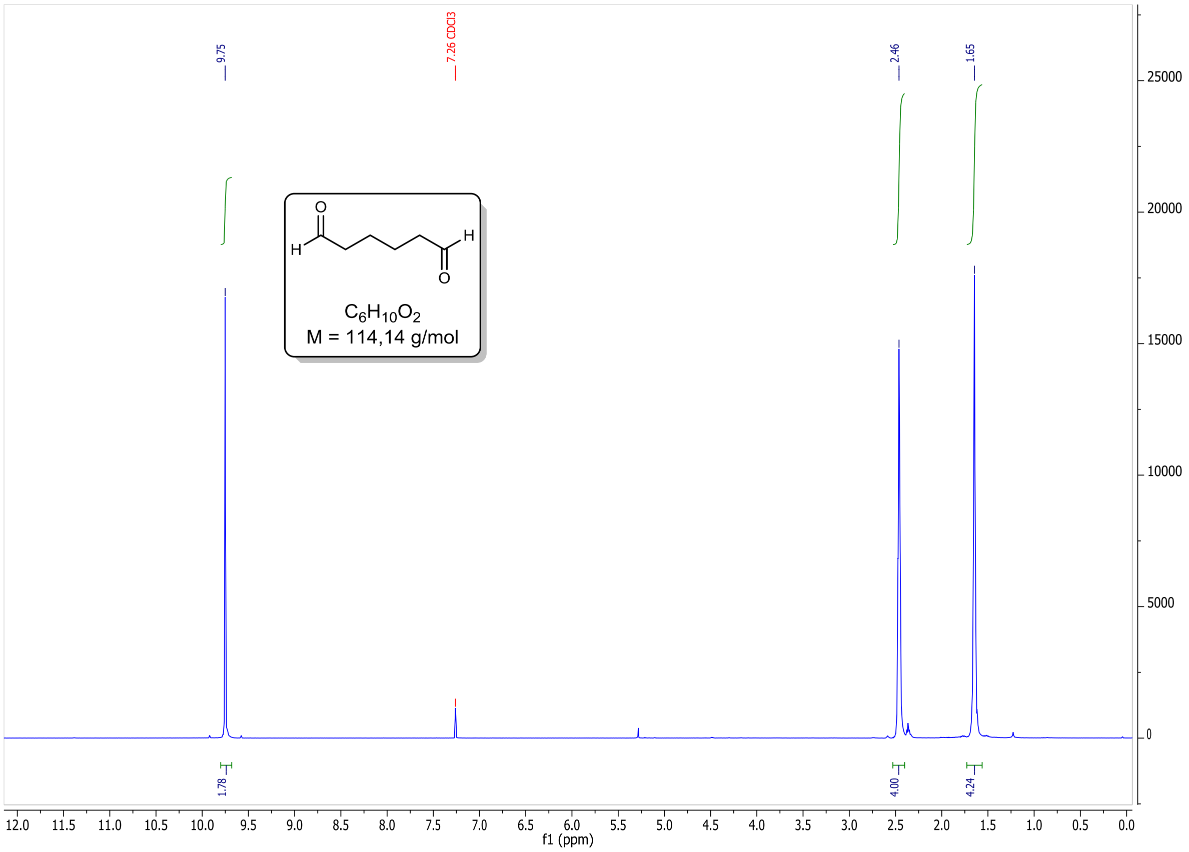

Supplementary Figure 17. ^{1}H-NMR spectra of adipaldehyde.

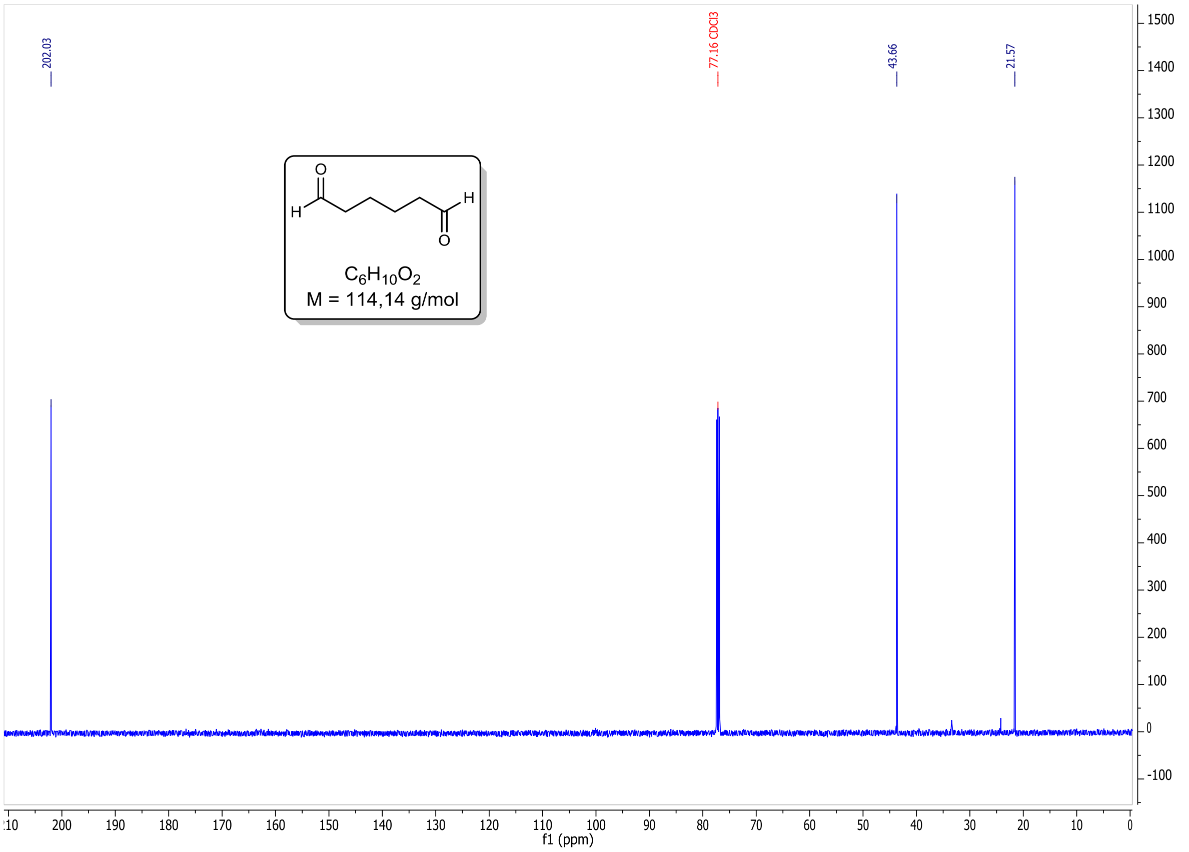

Supplementary Figure 18. ^{13}C-NMR spectra of glutaraldehyde.

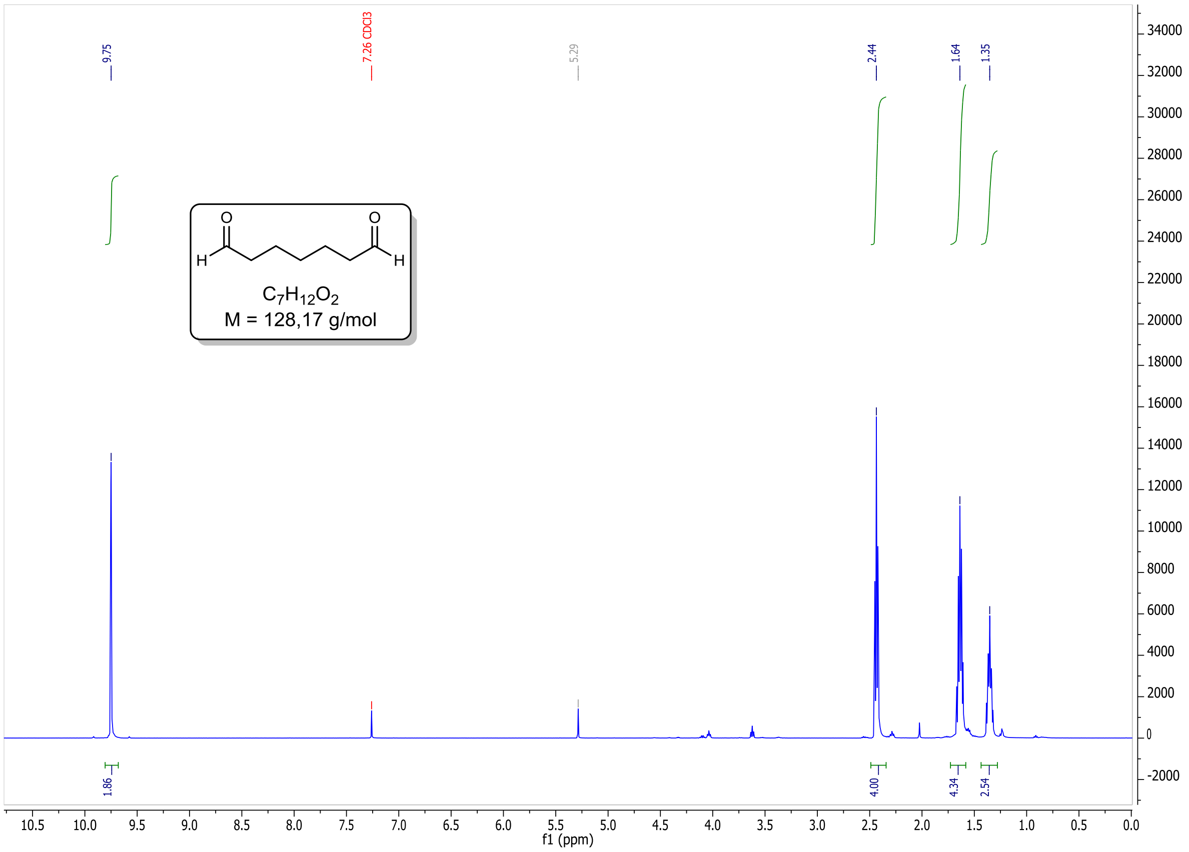

Supplementary Figure 19. ¹H-NMR spectra of heptanedial.

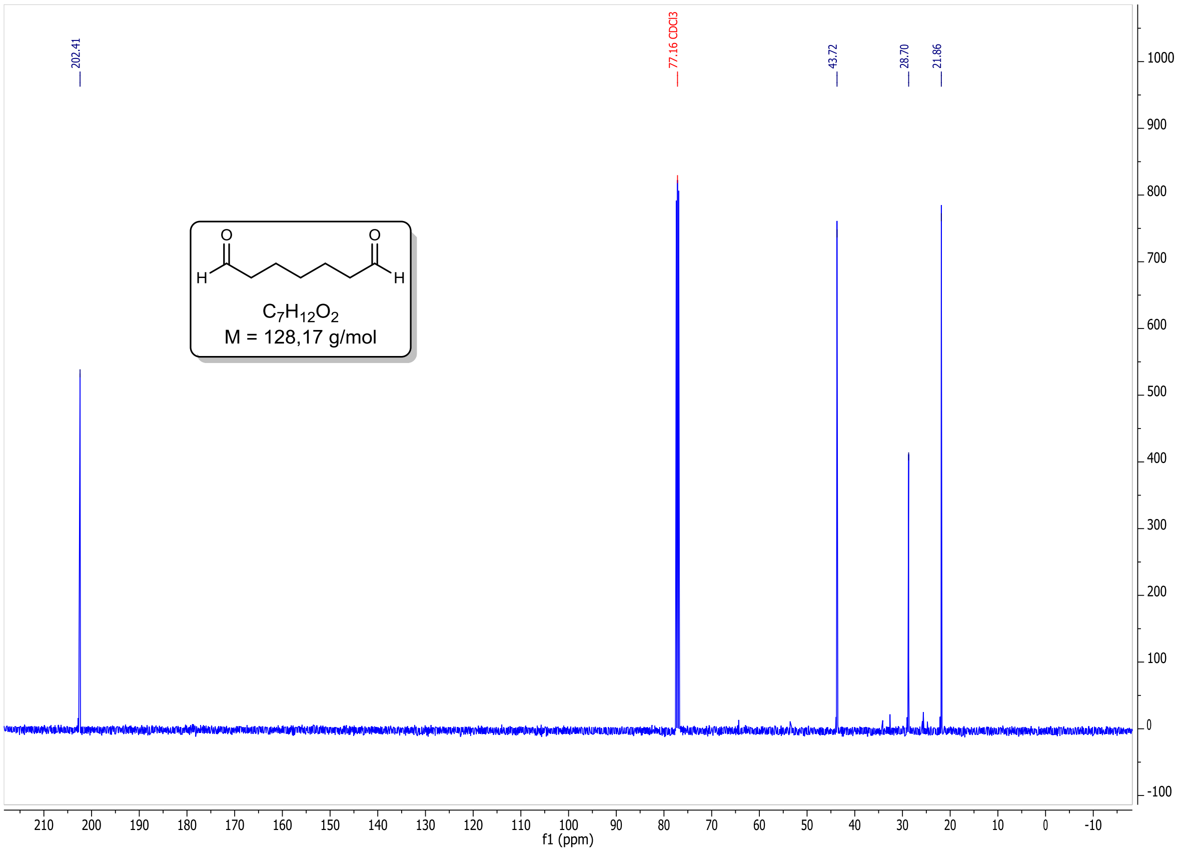

Supplementary Figure 20. ¹³C-NMR spectra of heptanedial.

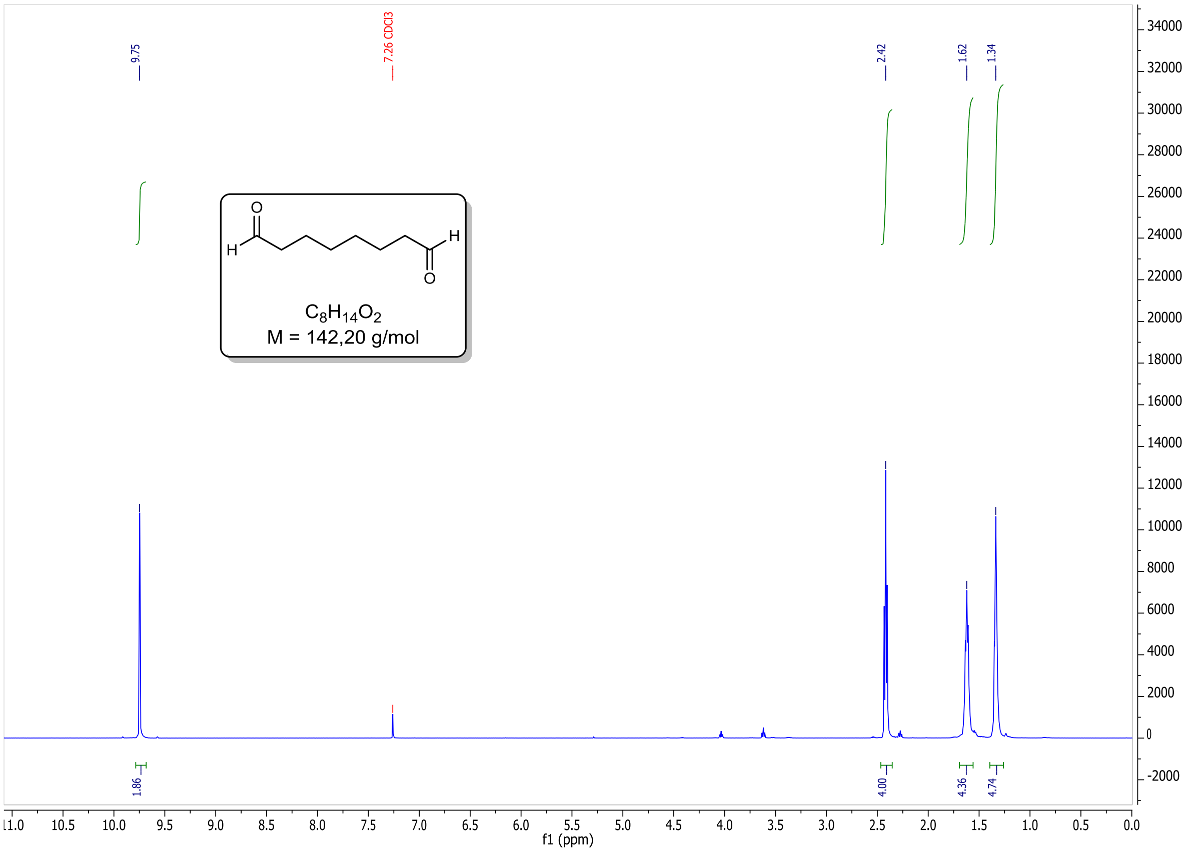

Supplementary Figure 21. ¹H-NMR spectra of octanedial.

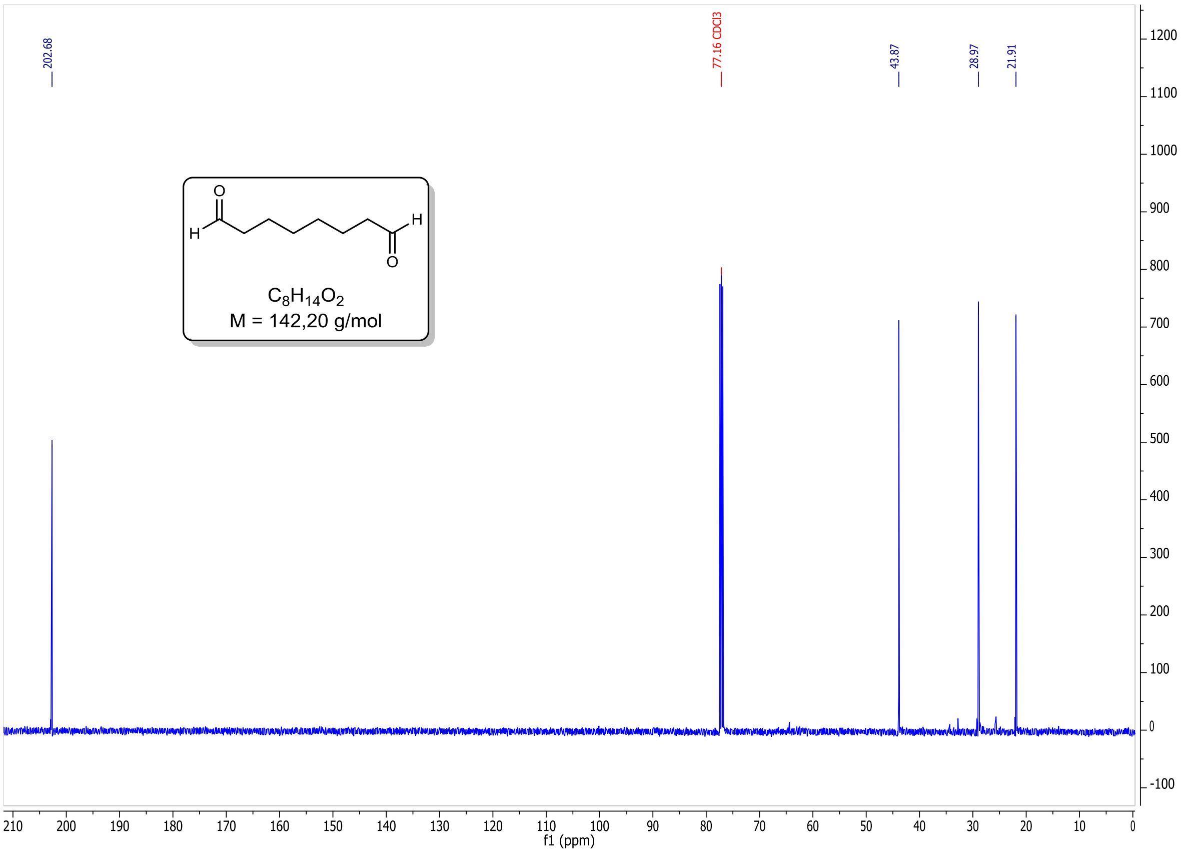

Supplementary Figure 22. ¹³C-NMR spectra of octanedial.

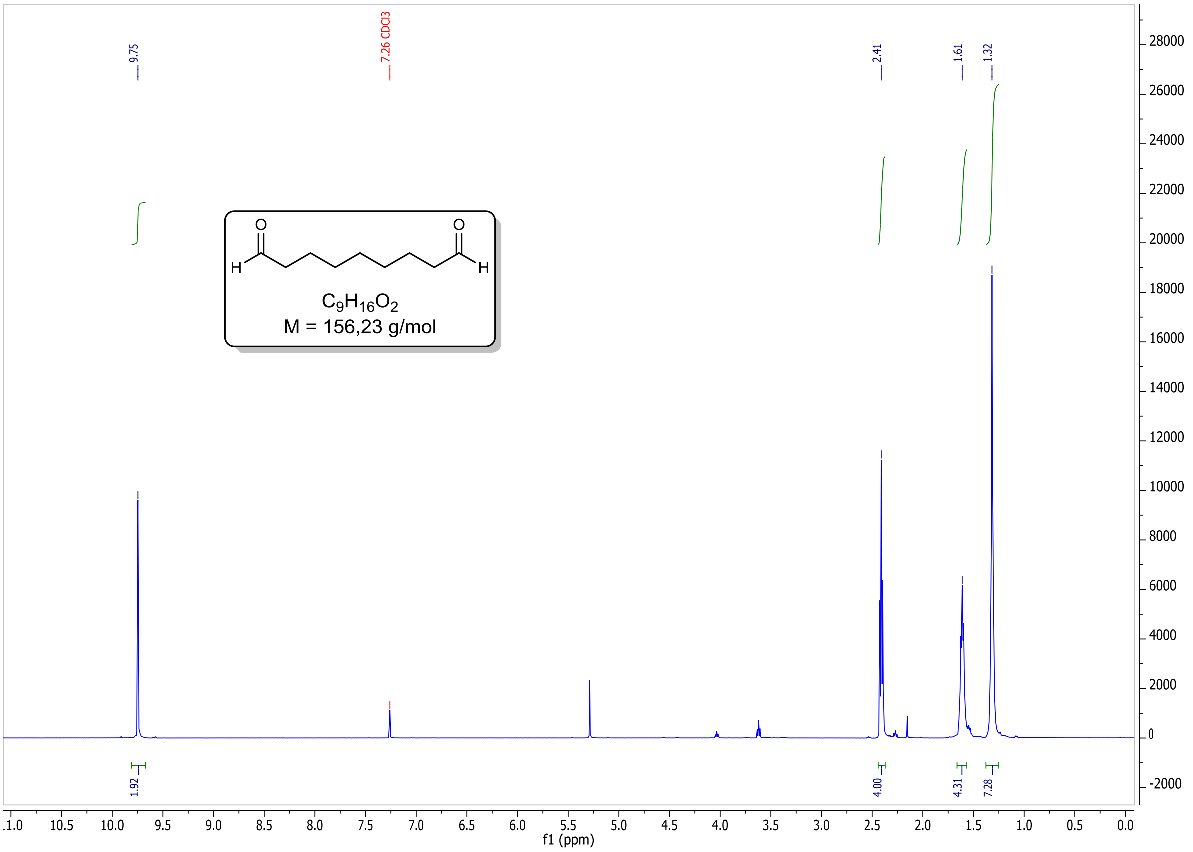

Supplementary Figure 23. **^{1}H-NMR spectra of nonanedial.**

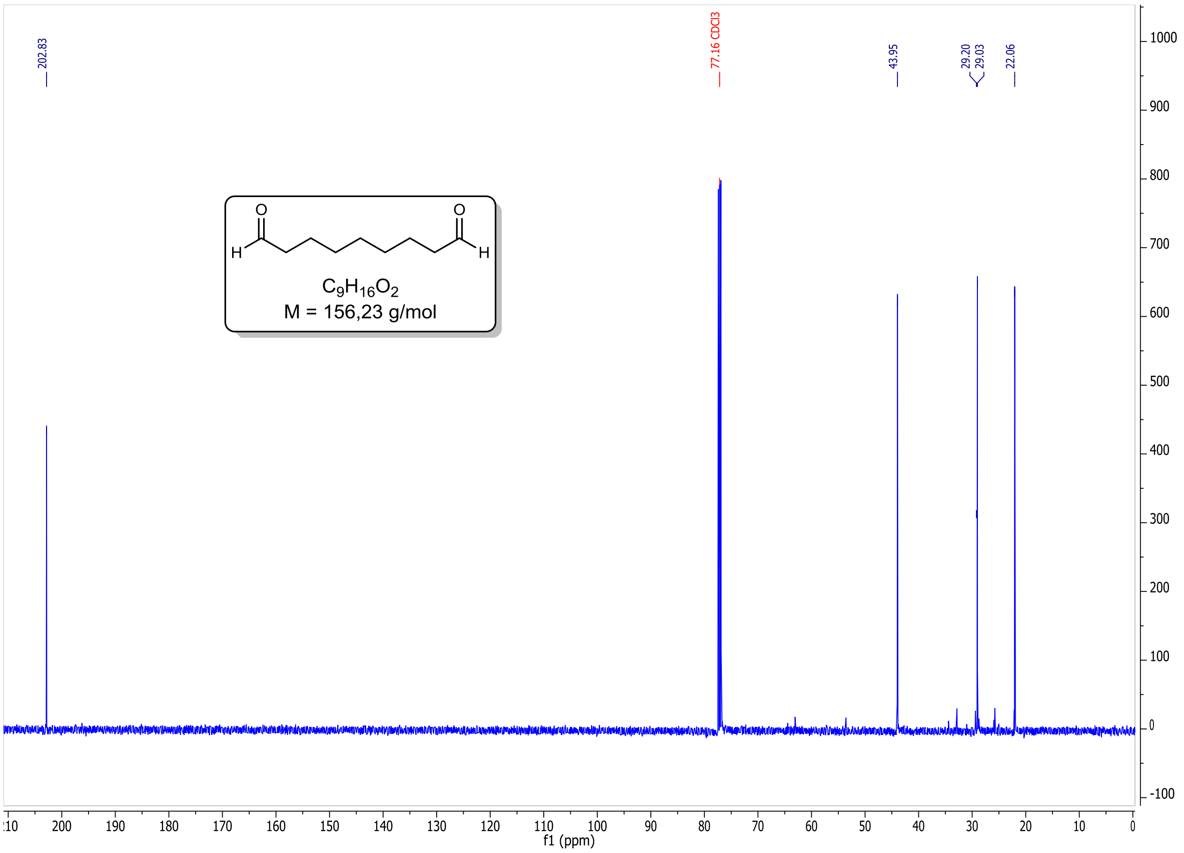

Supplementary Figure 24. **^{13}C-NMR spectra of nonanedial.**

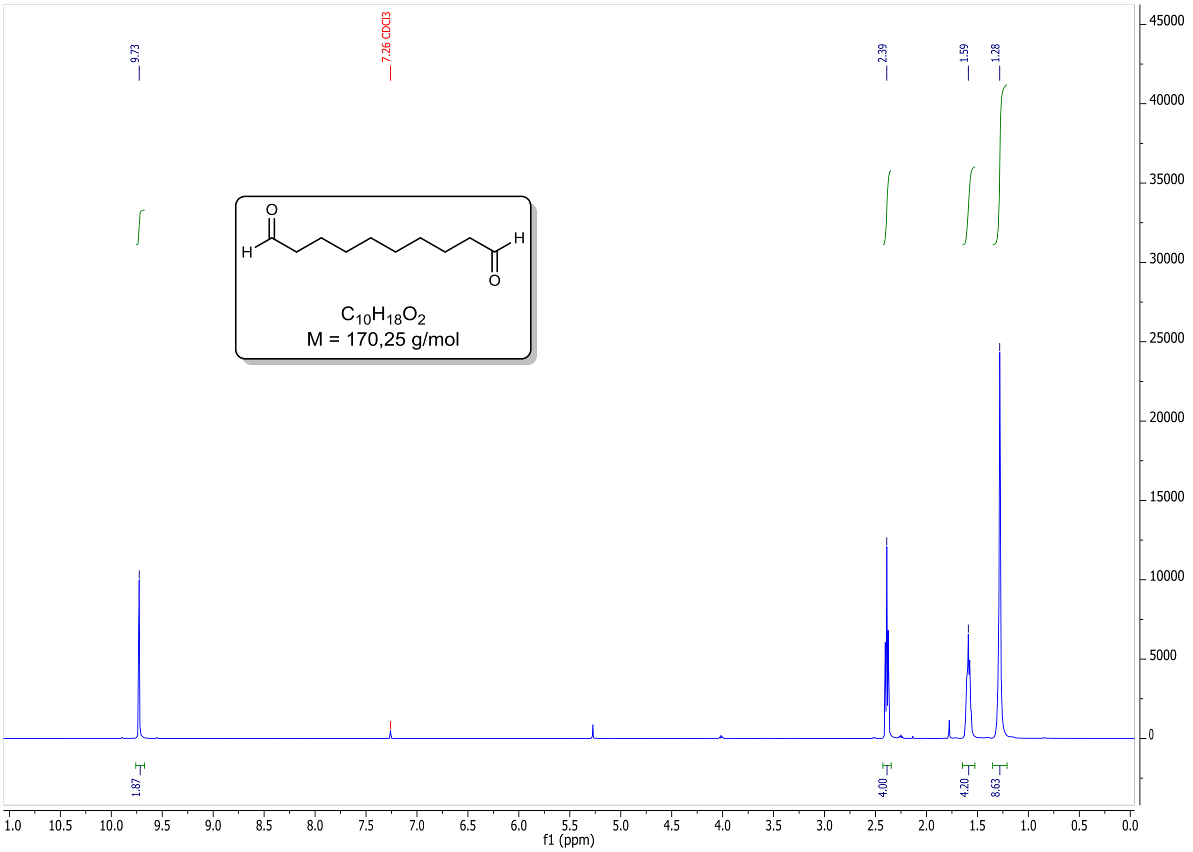

Supplementary Figure 25. ^{1}H-NMR spectra of decanedial.

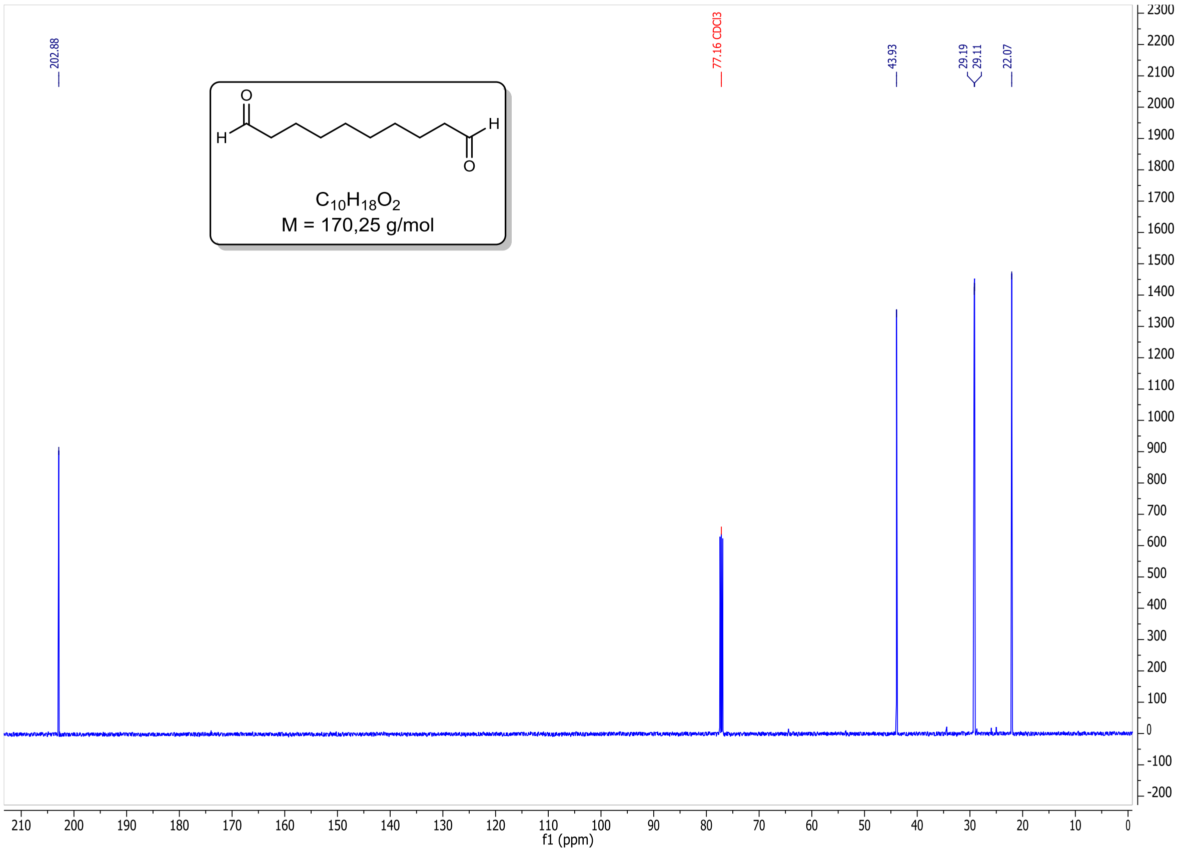

Supplementary Figure 26. ^{13}C-NMR spectra of decanedial.

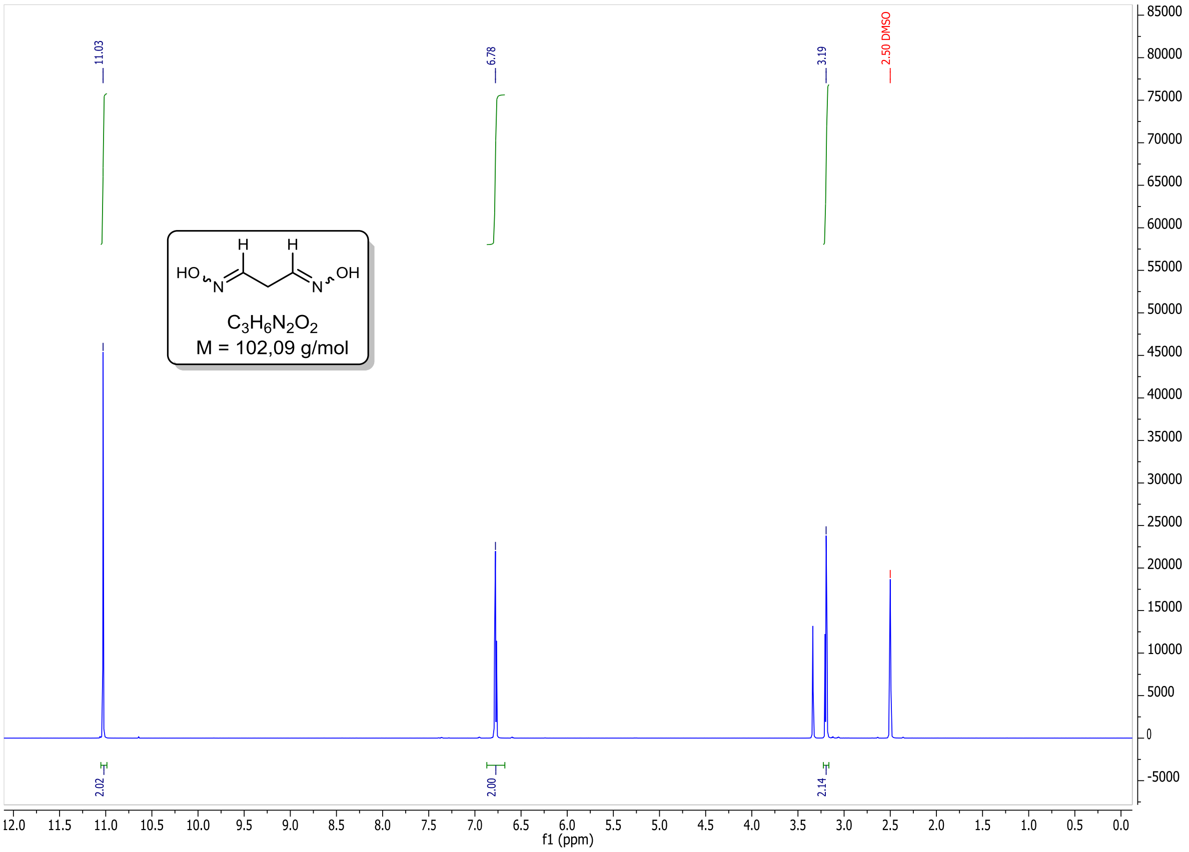

Supplementary Figure 27. ^{1}H-NMR spectra of malonaldehyde dioxime (1a).

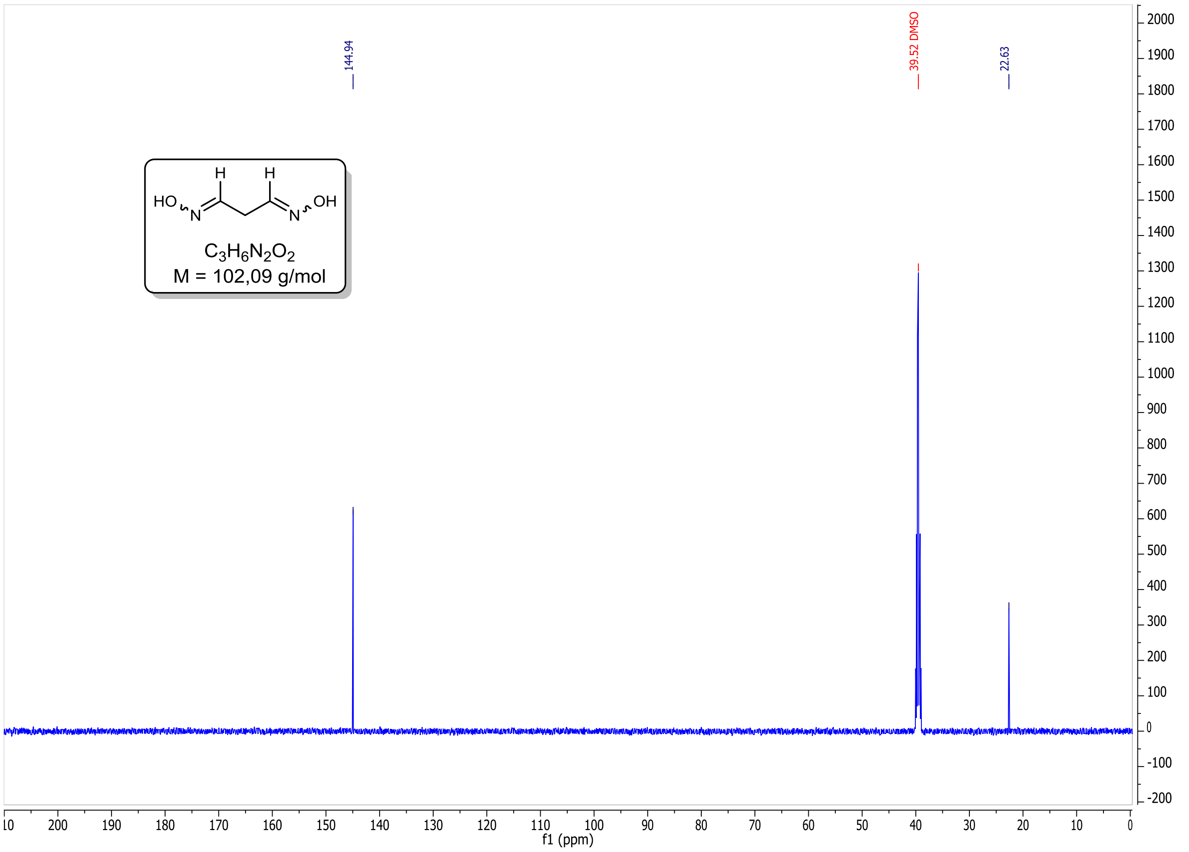

Supplementary Figure 28. ^{13}C-NMR spectra of malonaldehyde dioxime (1a).

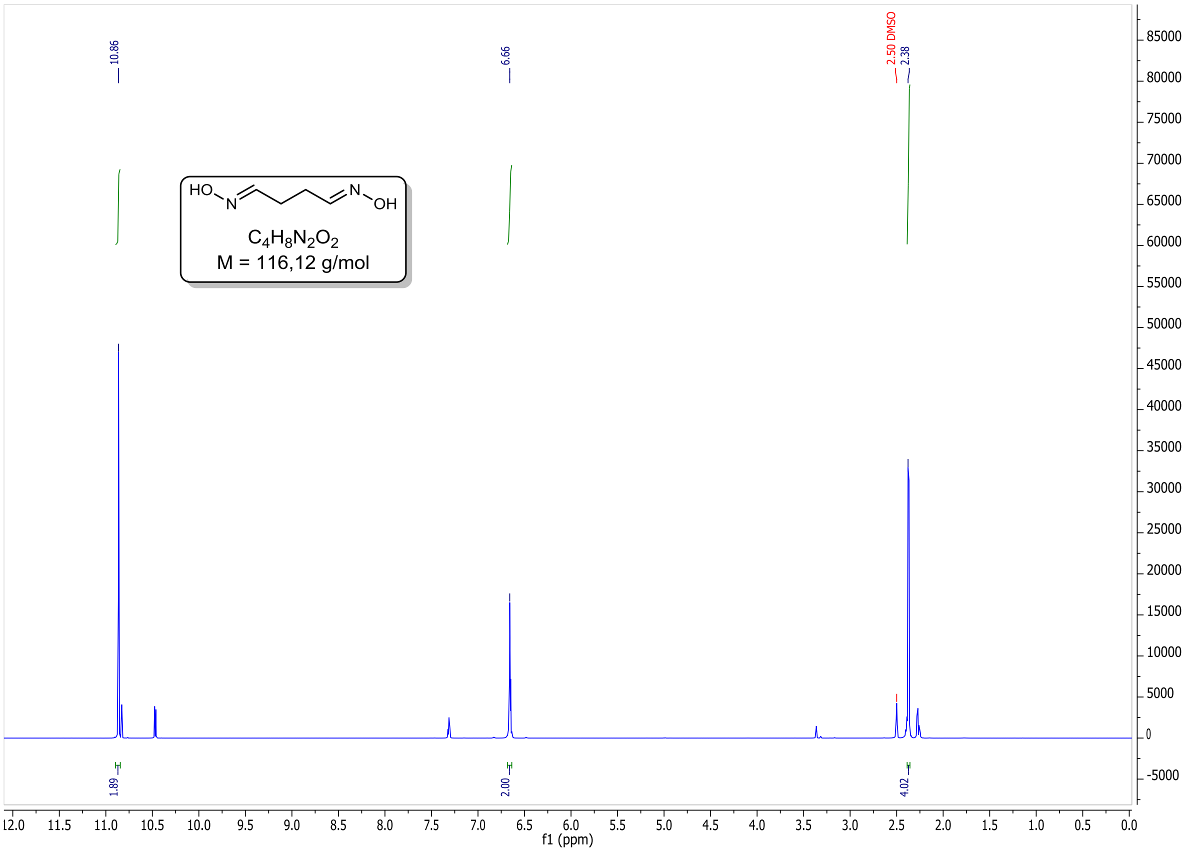

Supplementary Figure 29. ^{1}H-NMR spectra of succinaldehyde dioxime (1b).

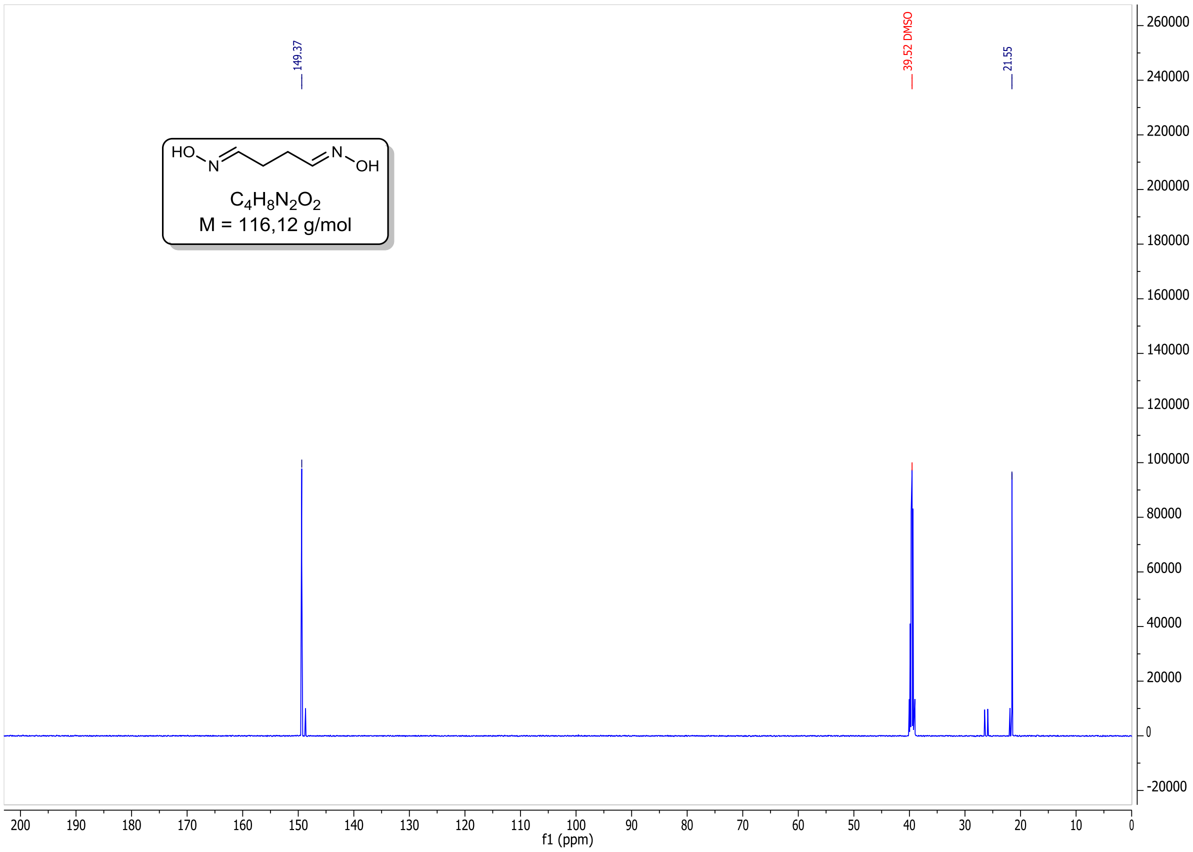

Supplementary Figure 30. ^{13}C-NMR spectra of succinaldehyde dioxime (1b).

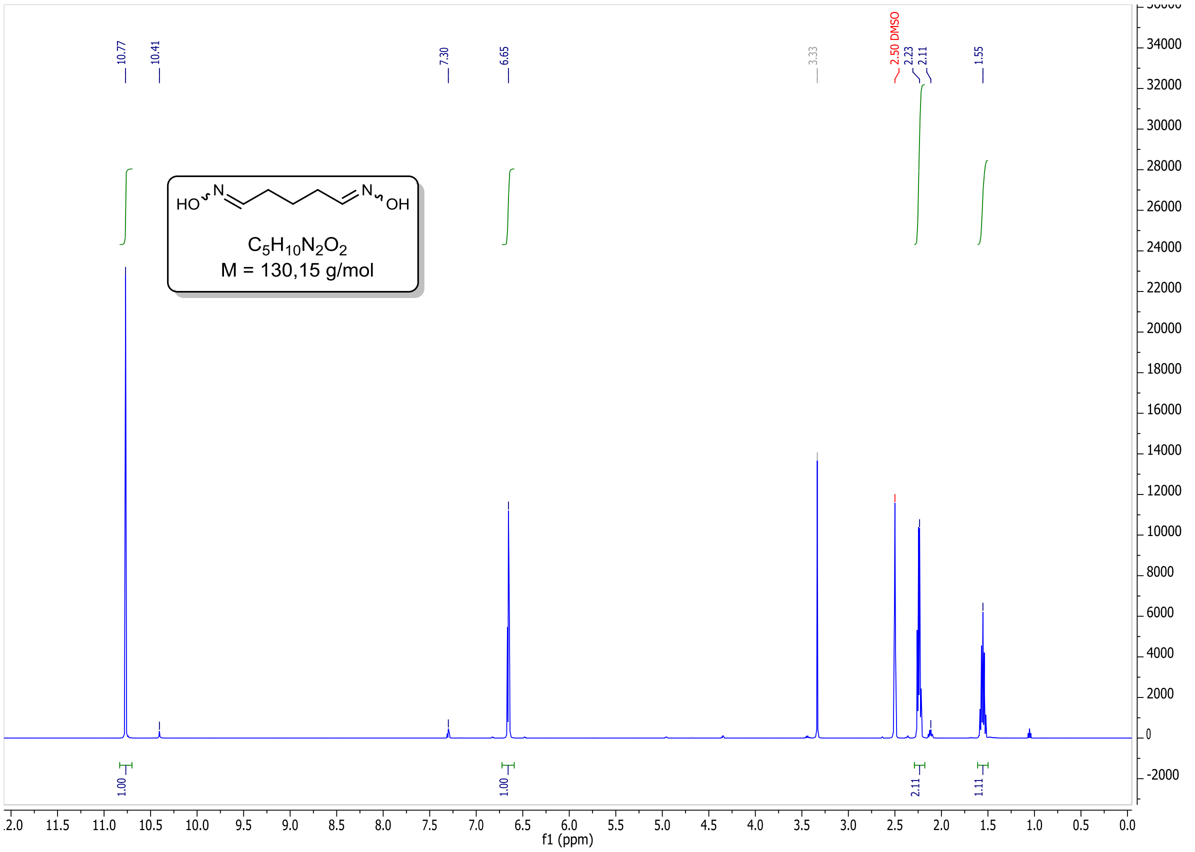

Supplementary Figure 31. ^{1}H-NMR spectra of glutaraldehyde dioxime (1c).

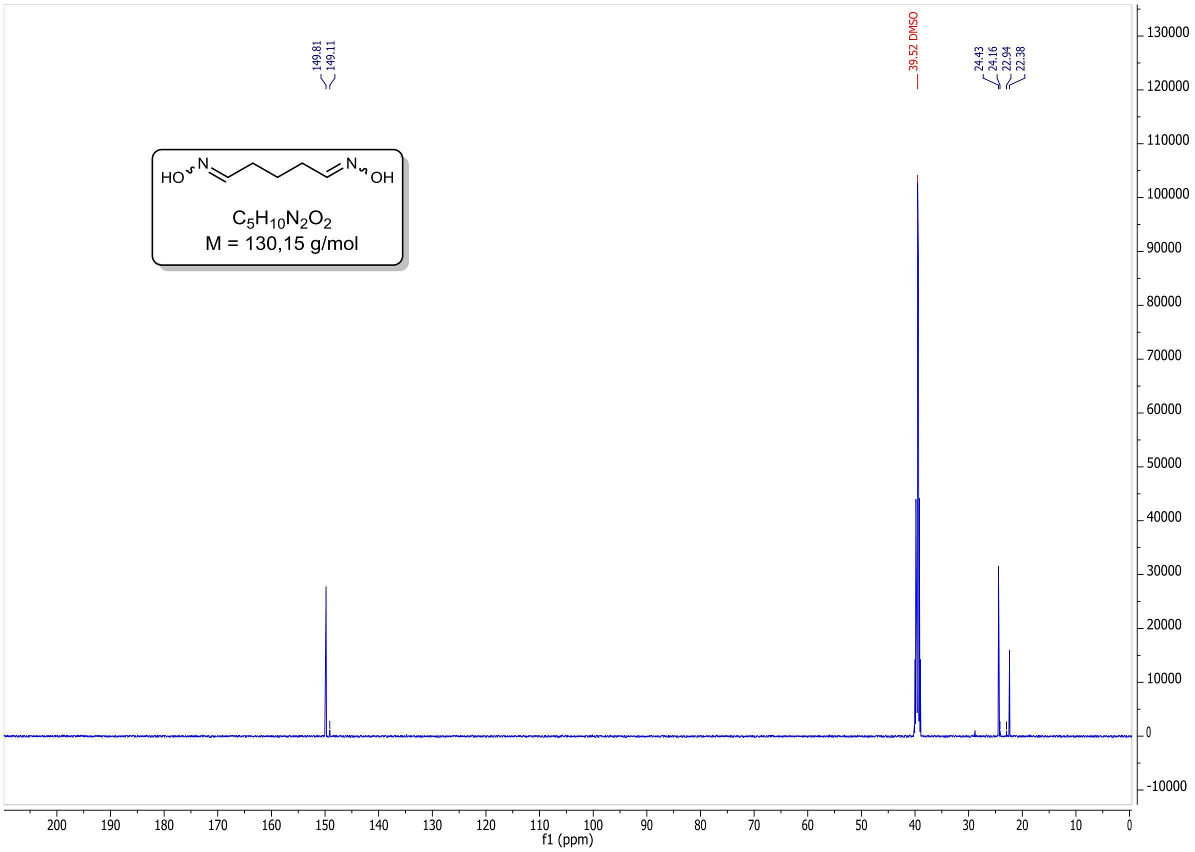

Supplementary Figure 32. ^{13}C-NMR spectra of glutaraldehyde dioxime (1c).

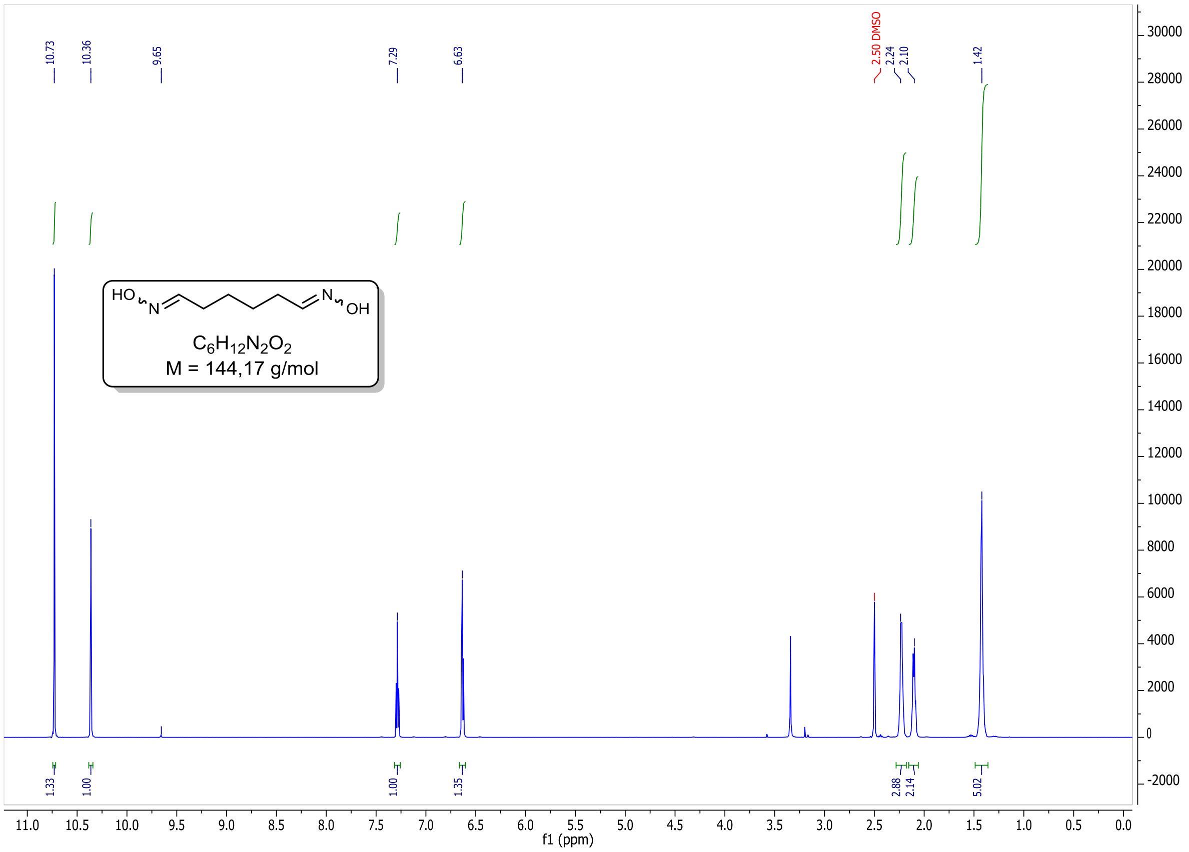

Supplementary Figure 33. ^{1}H-NMR spectra of adipaldehyde dioxime (1d).

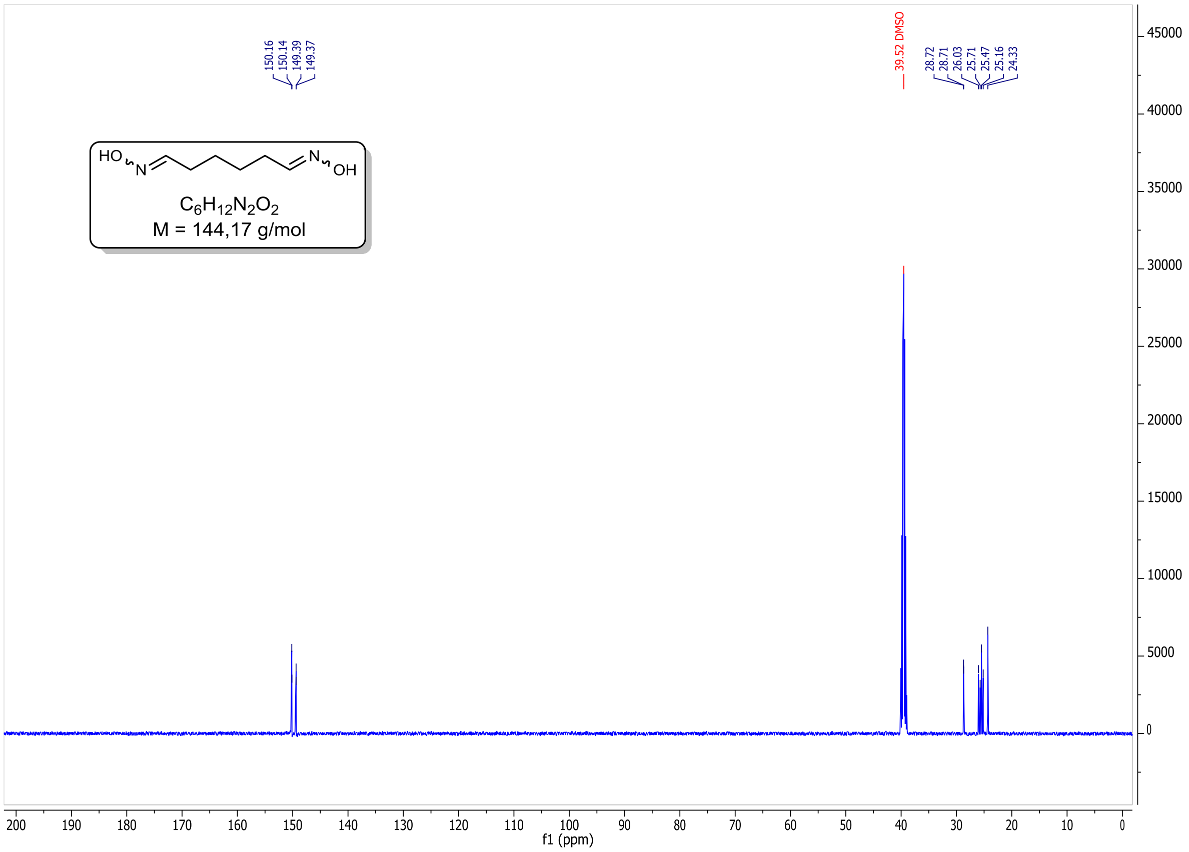

Supplementary Figure 34. ^{13}C-NMR spectra of adipaldehyde dioxime (1d).

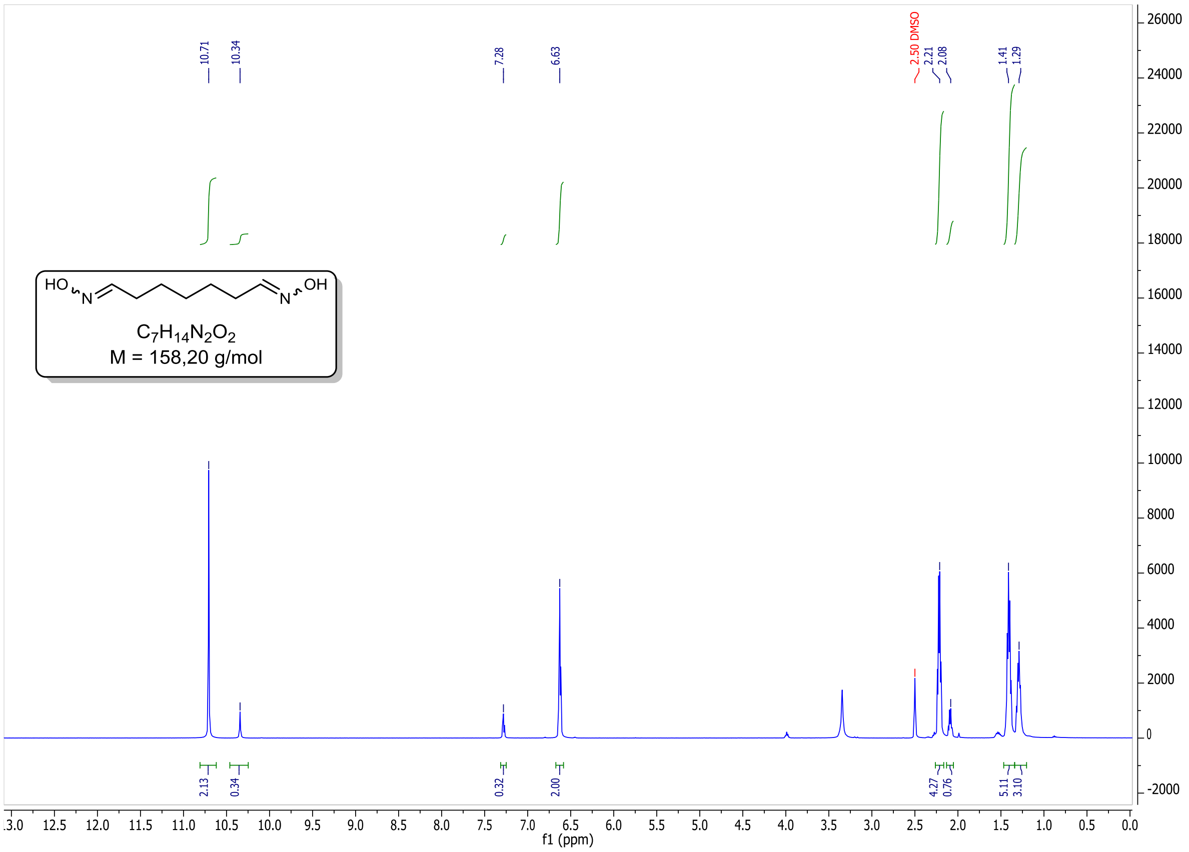

Supplementary Figure 35. ^{1}H-NMR spectra of heptanedial dioxime (1e).

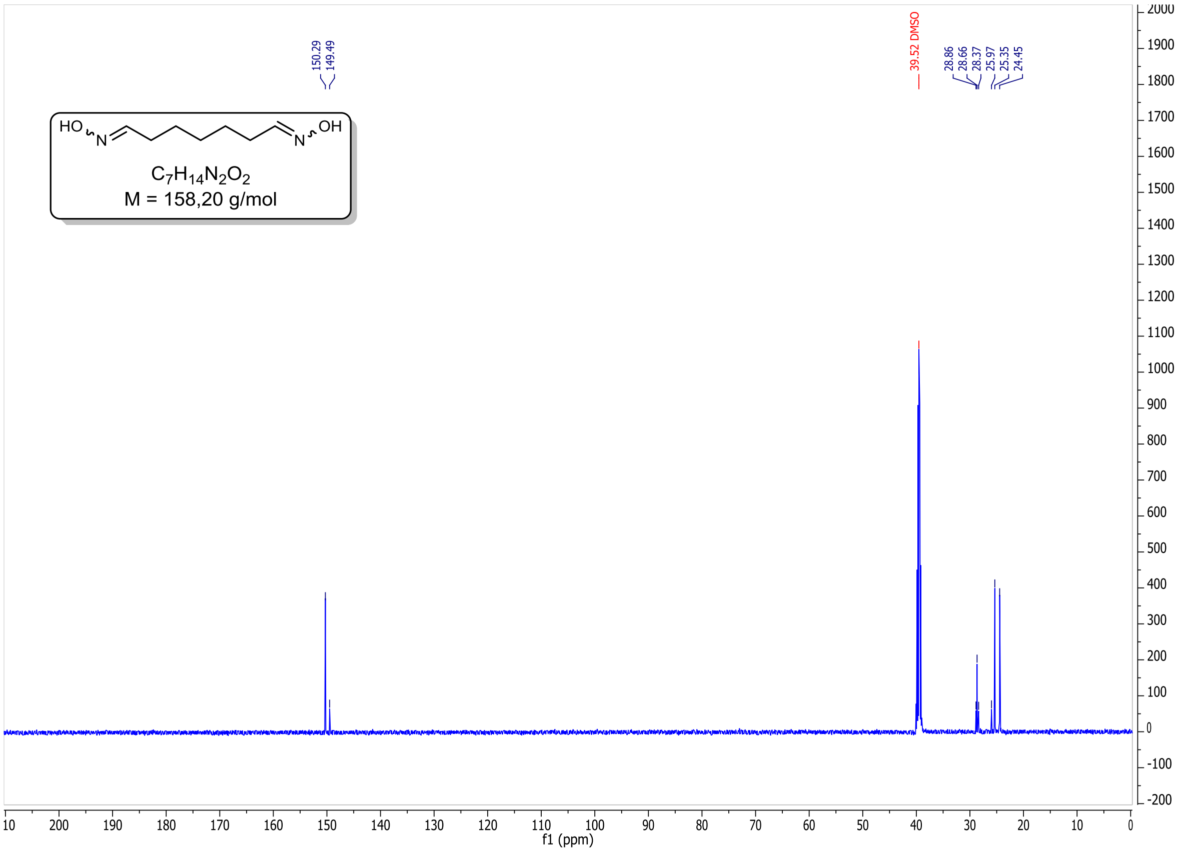

Supplementary Figure 36. ^{13}C-NMR spectra of heptanedial dioxime (1e).

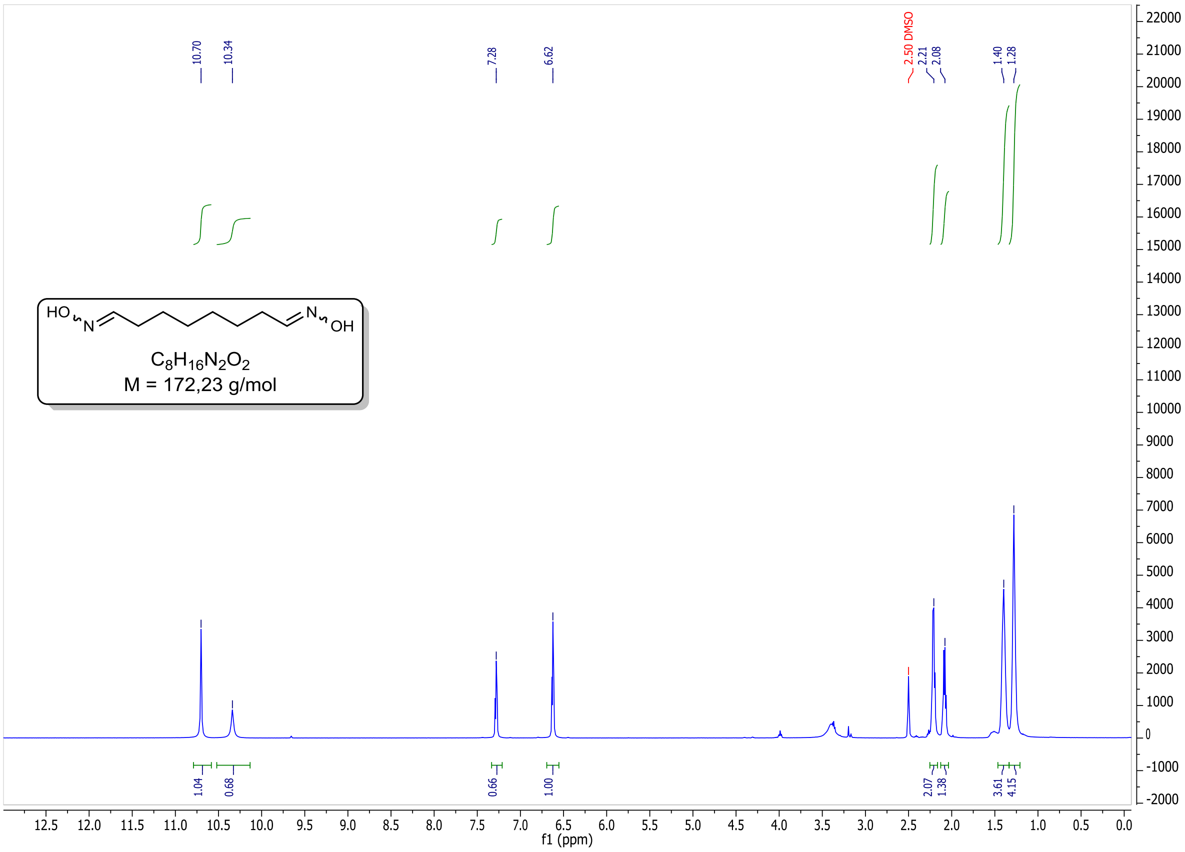

Supplementary Figure 37. **^{1}H-NMR spectra of octanedial dioxime (1f).**

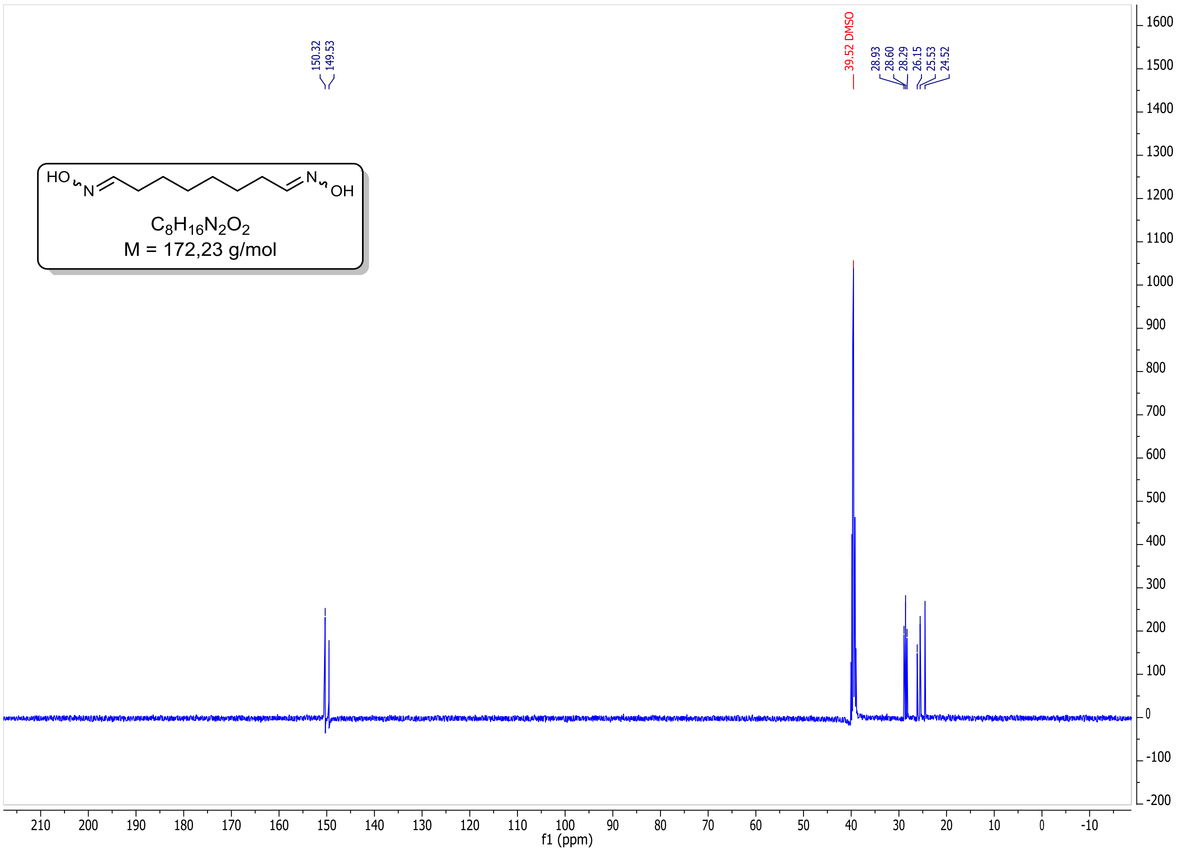

Supplementary Figure 38. **^{13}C-NMR spectra of octanedial dioxime (1f).**

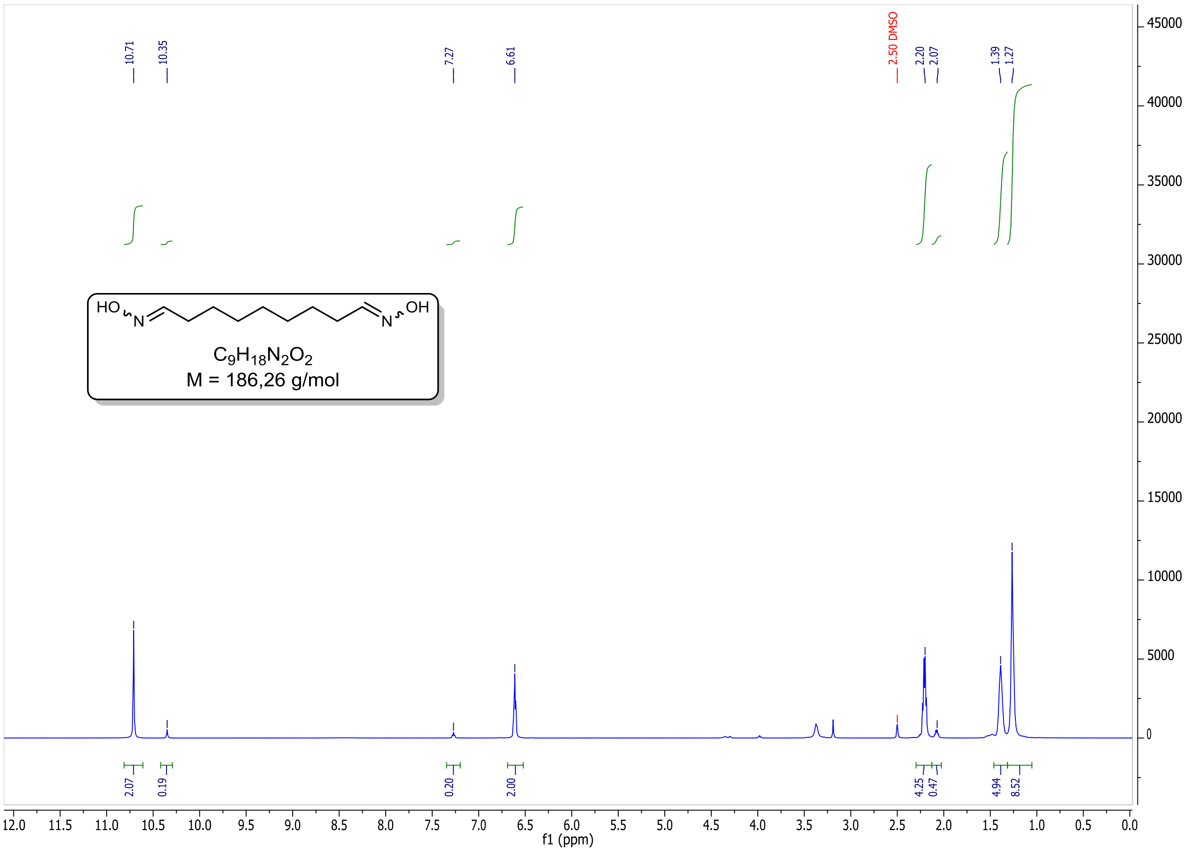

Supplementary Figure 39. ^{1}H-NMR spectra of nonanedial dioxime (1g).

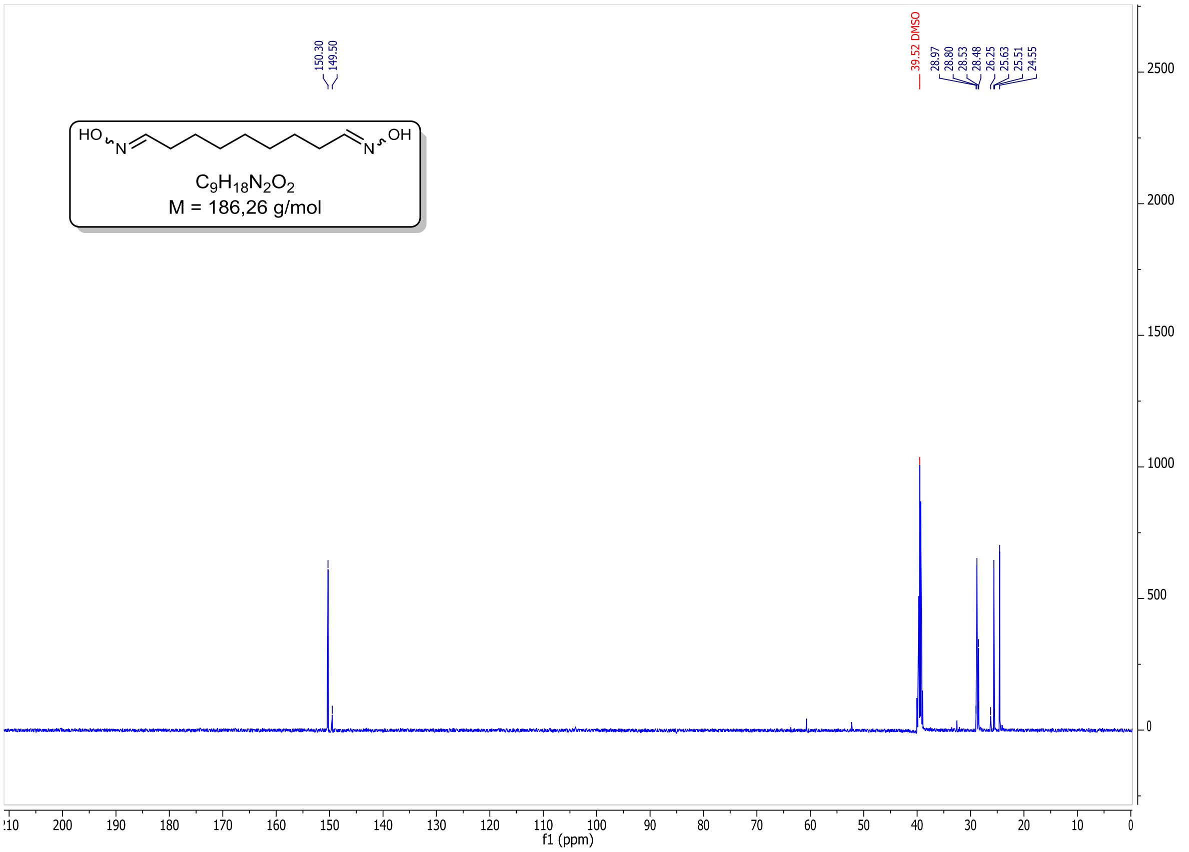

Supplementary Figure 40. ^{13}C-NMR spectra of nonanedial dioxime (1g).

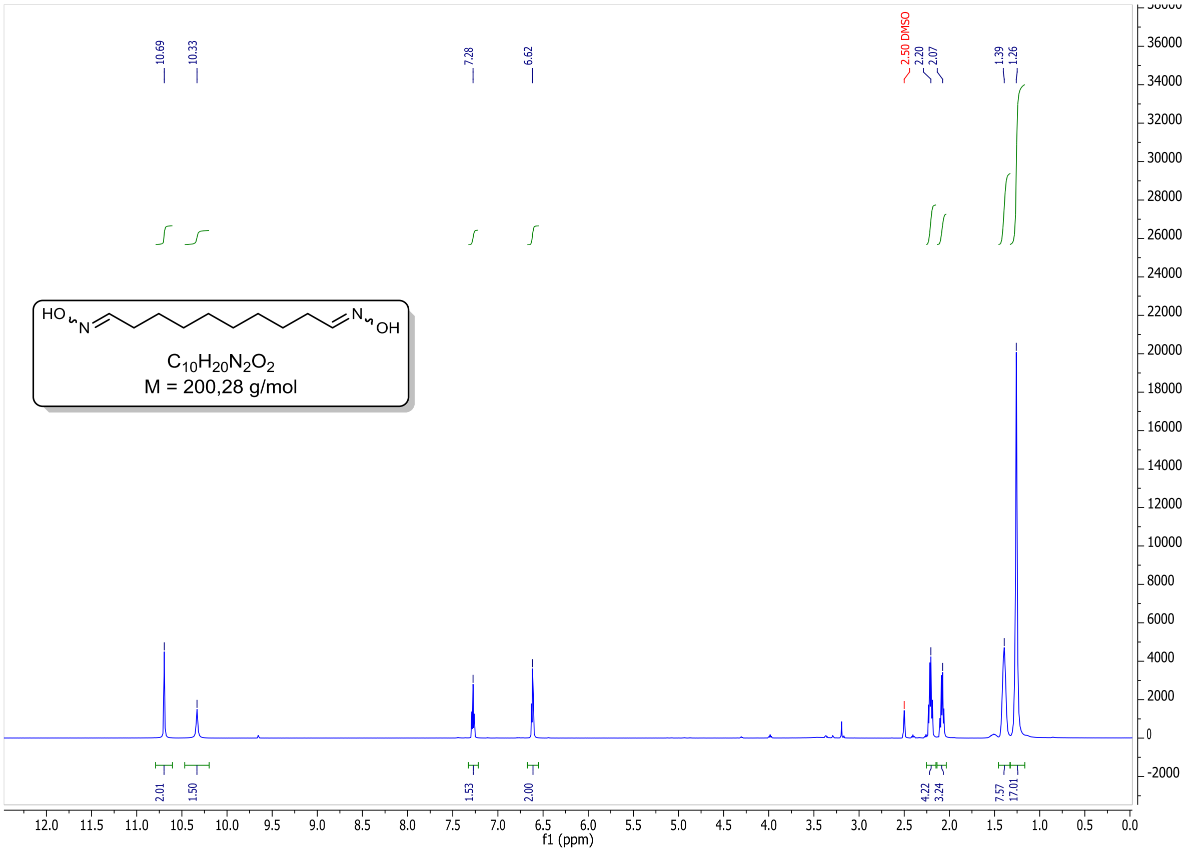

Supplementary Figure 41. ^{1}H-NMR spectra of decanedial dioxime (1h).

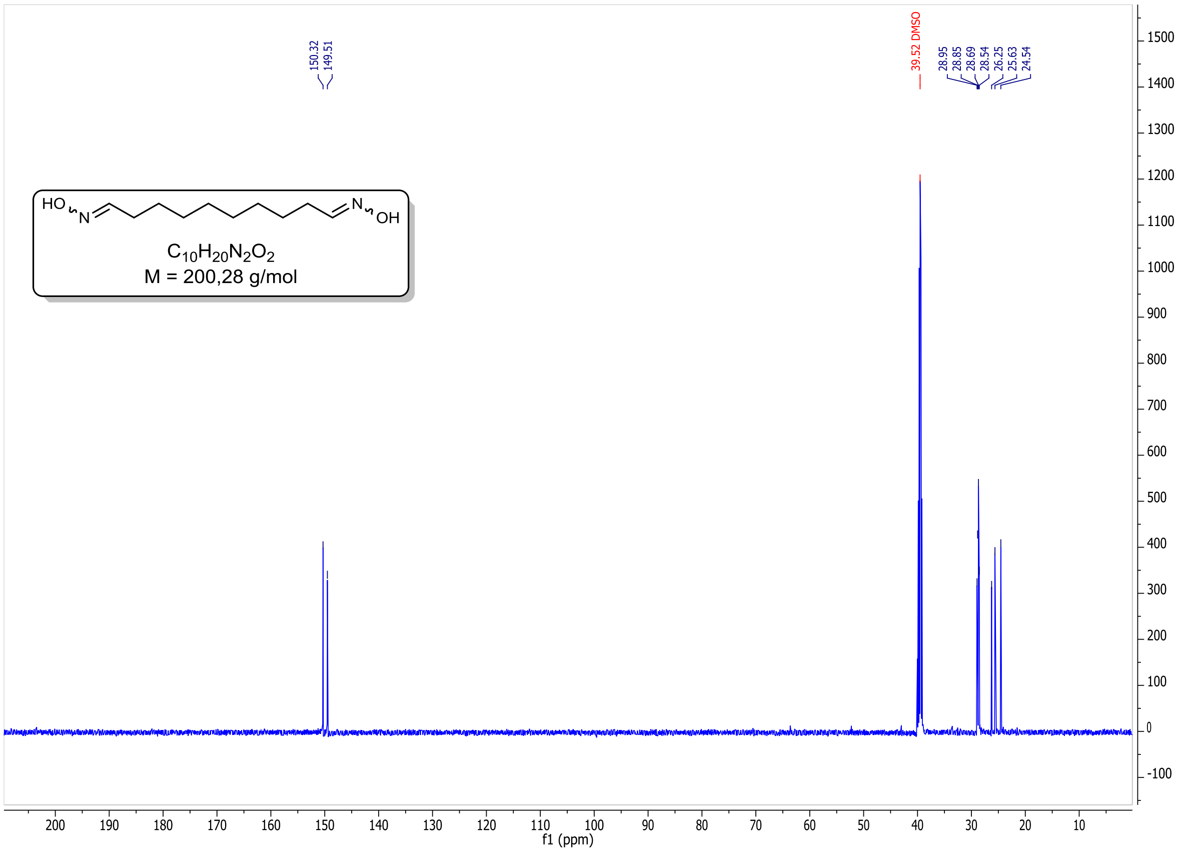

Supplementary Figure 42. ^{13}C-NMR spectra of decanedial dioxime (1h).

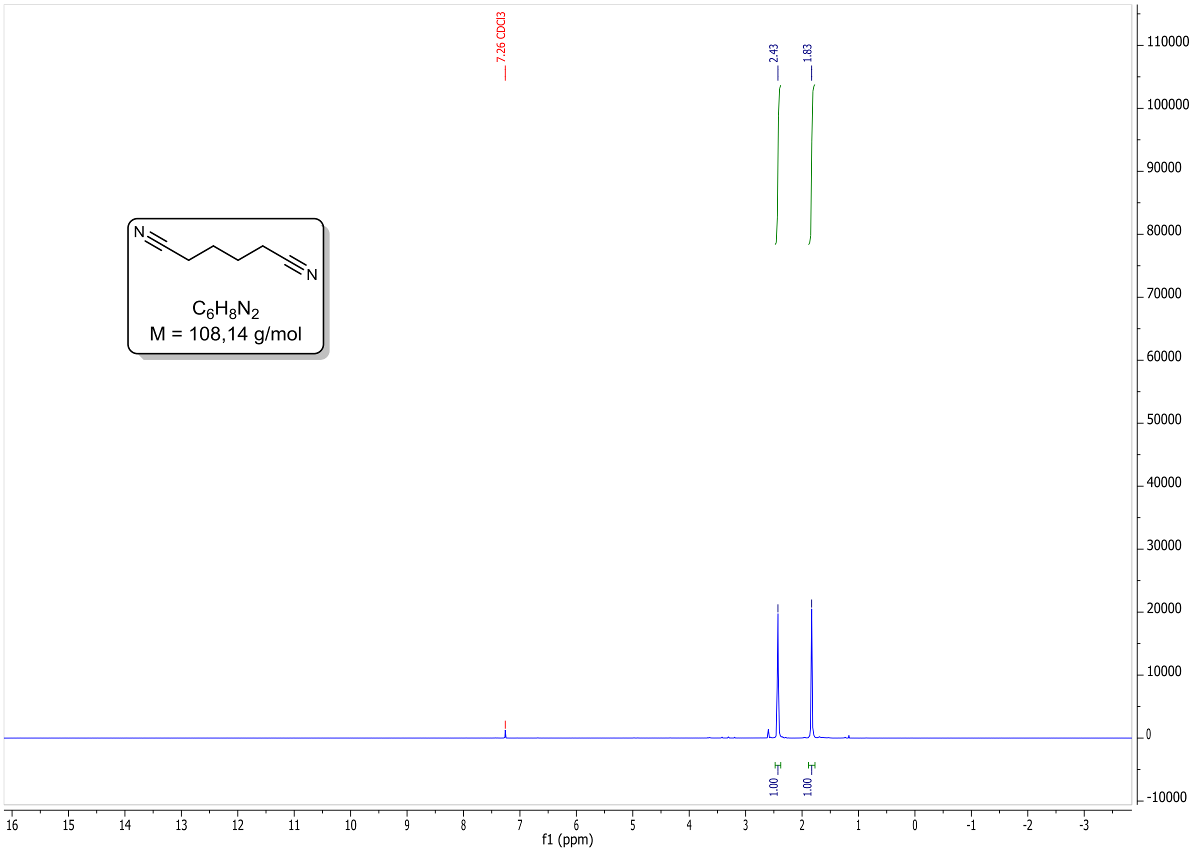

Supplementary Figure 43. ^{1}H-NMR spectra of adiponitrile (2d).

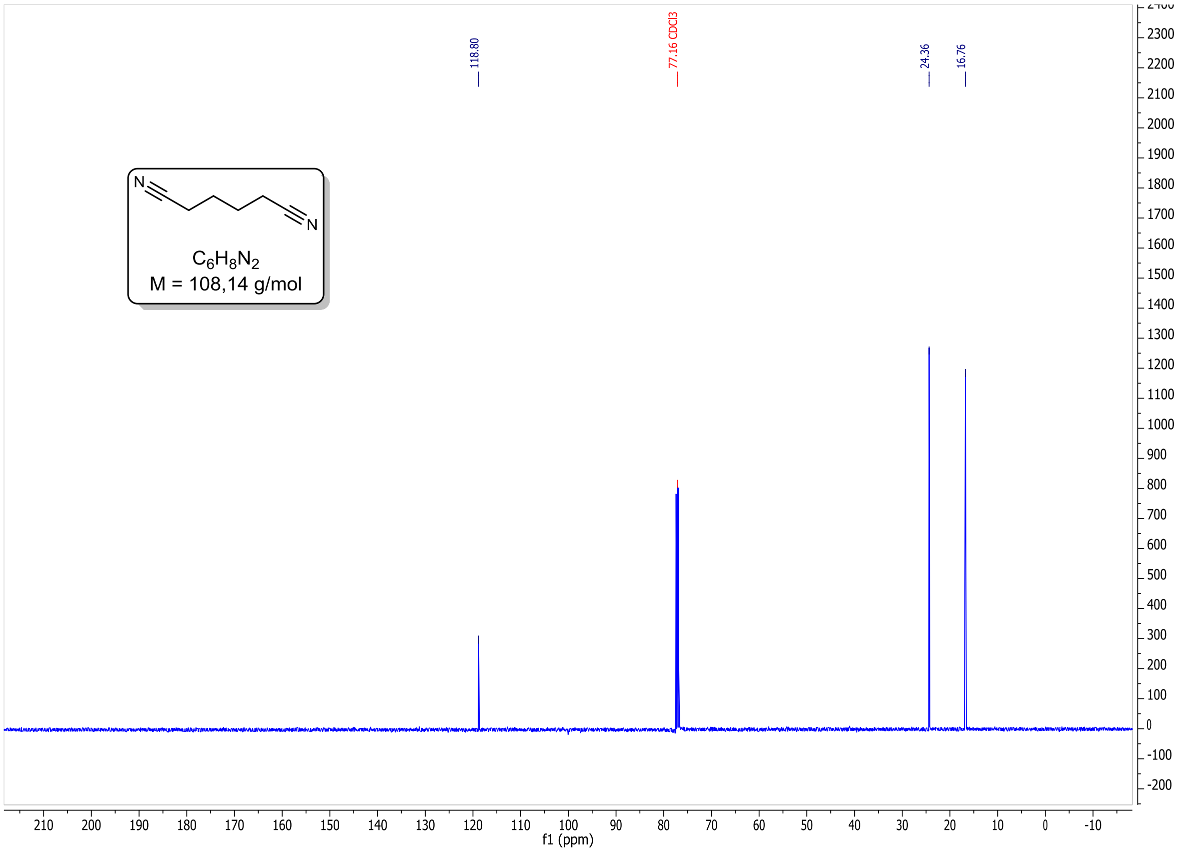

Supplementary Figure 44. ^{13}C-NMR spectra of adiponitrile (2d).

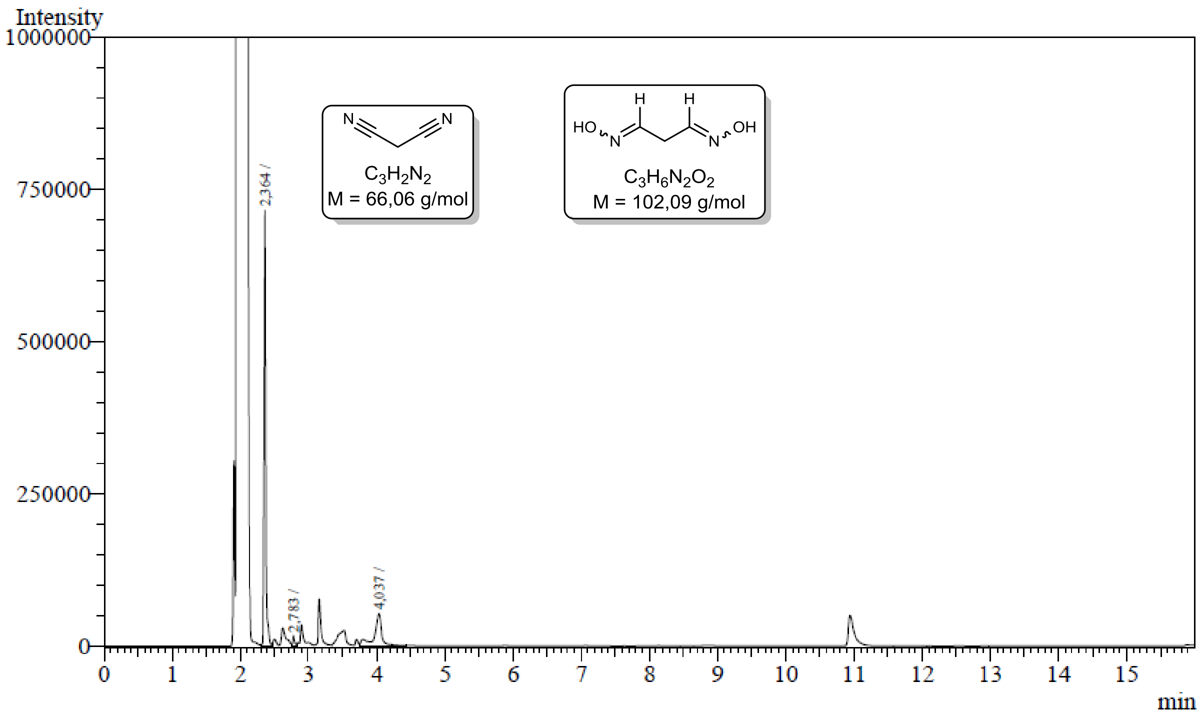

Supplementary Figure 45. GC-chromatogram of malononitrile (2a) and malonaldehyde dioxime (1a).

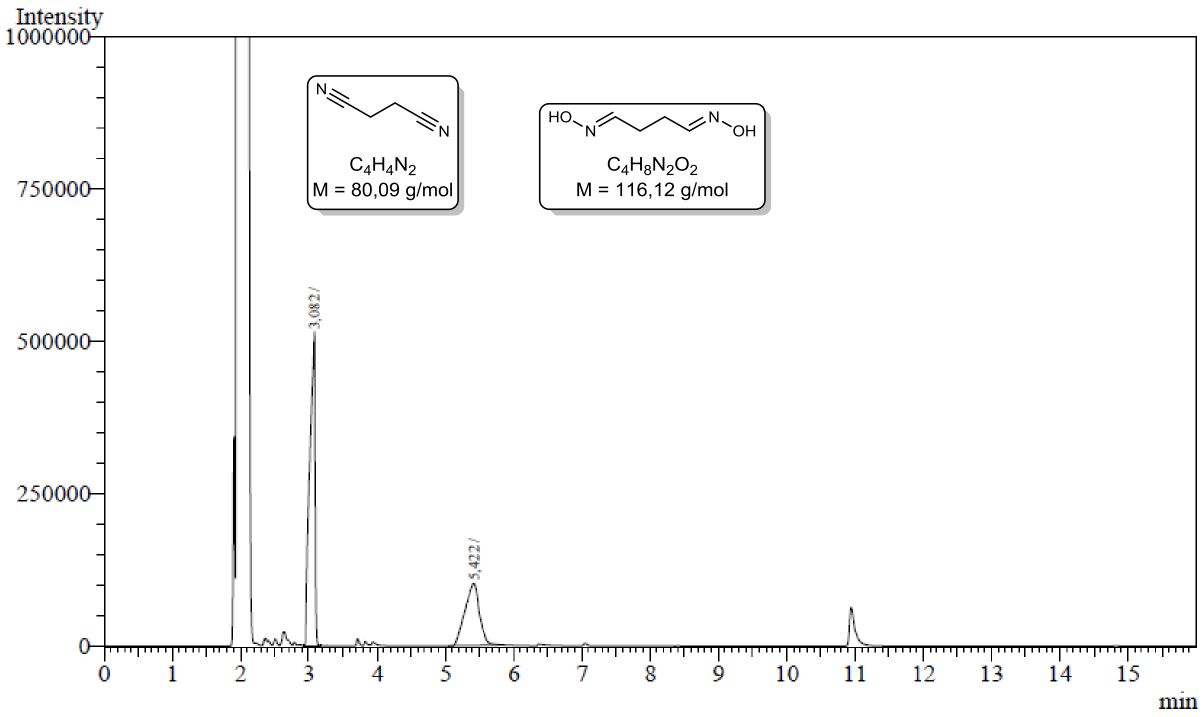

Supplementary Figure 46. GC-chromatogram of succinonitrile (2b) and succinaldehyde dioxime (1b).

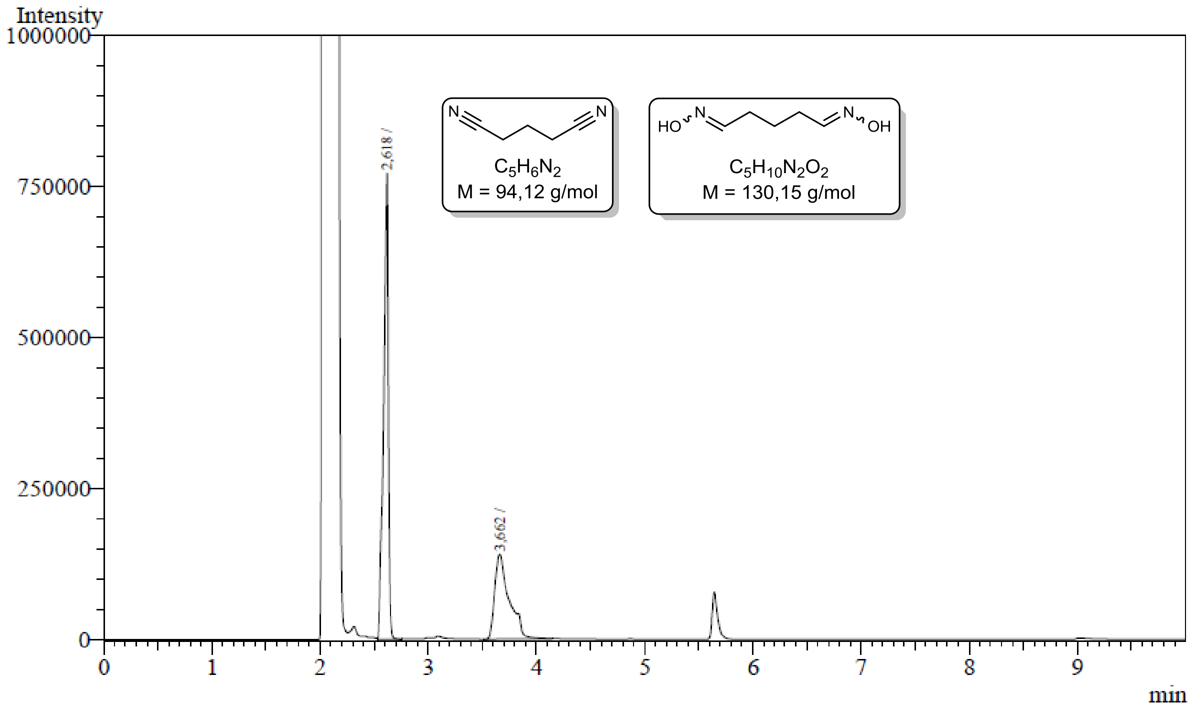

Supplementary Figure 47. GC-chromatogram of glutaronitrile (2c) and glutaraldehyde dioxime (1c).

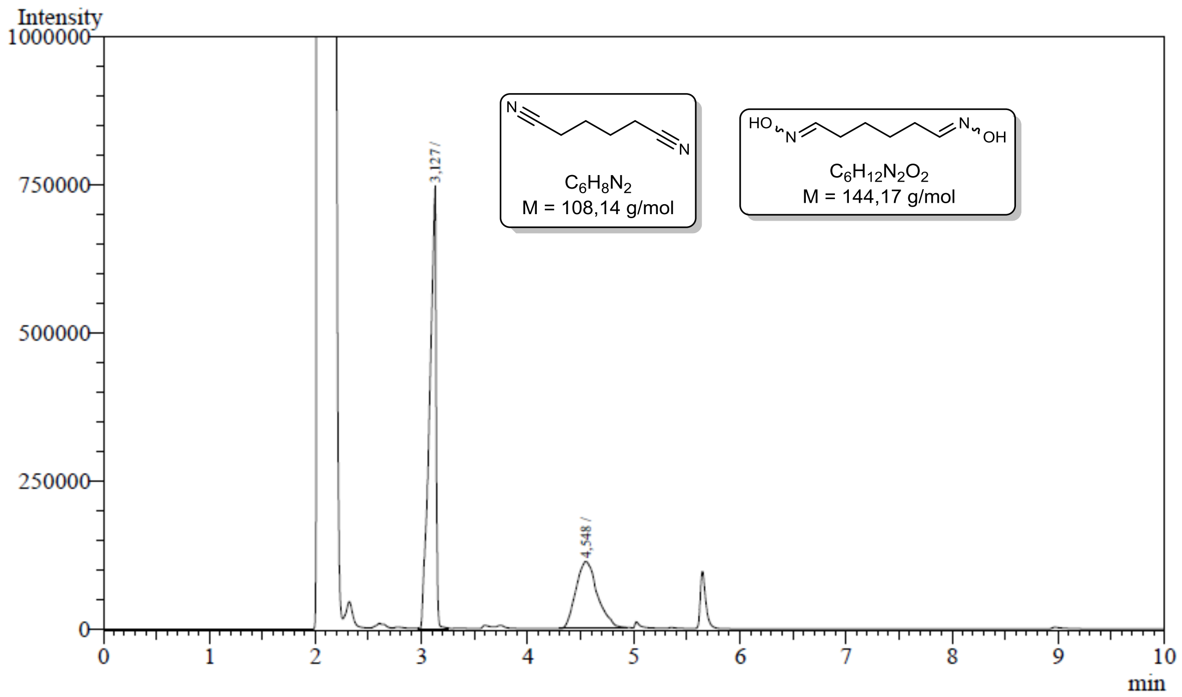

Supplementary Figure 48. GC-chromatogram of adiponitrile (2d) and adipaldehyde dioxime (1d).

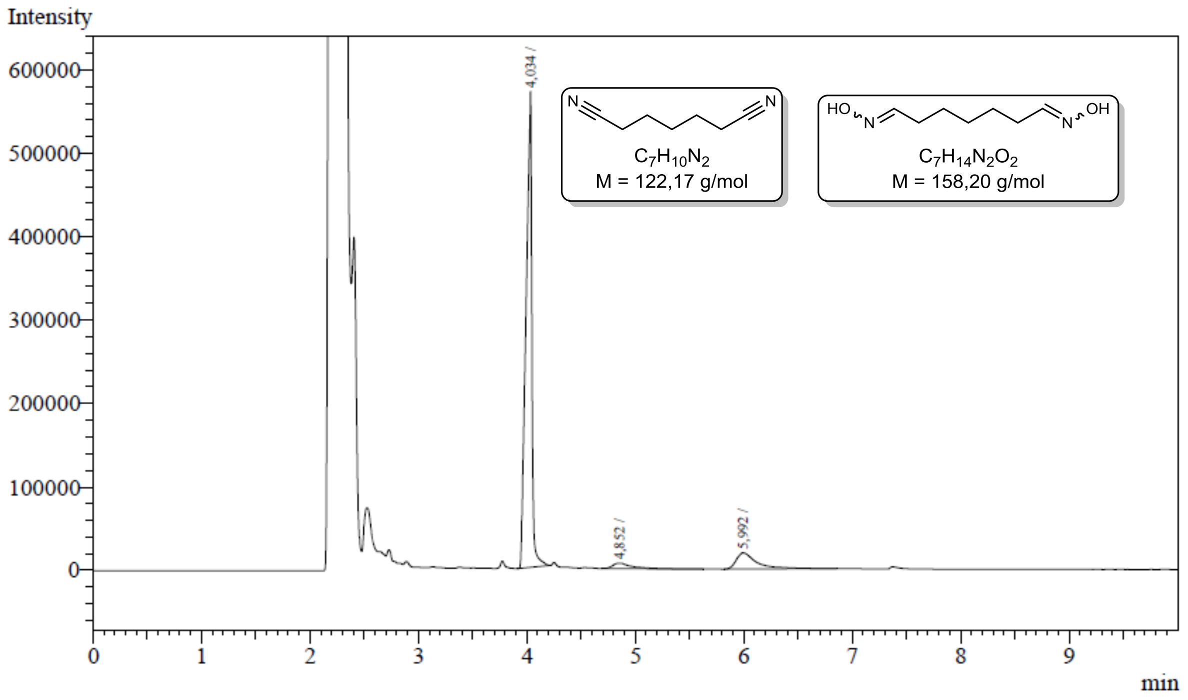

Supplementary Figure 49. GC-chromatogram of heptanedinitrile (2e) and heptanedial dioxime (1e).

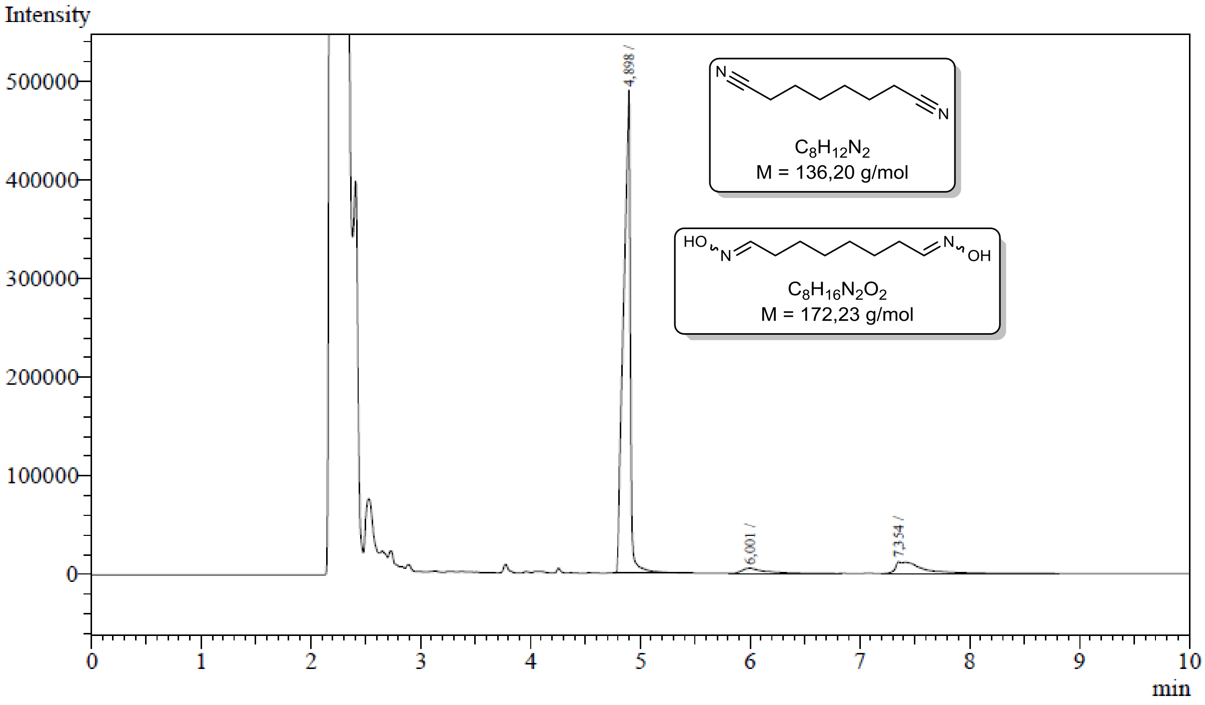

Supplementary Figure 50. GC-chromatogram of octanedinitrile (2f) and octanedial dioxime (1f).

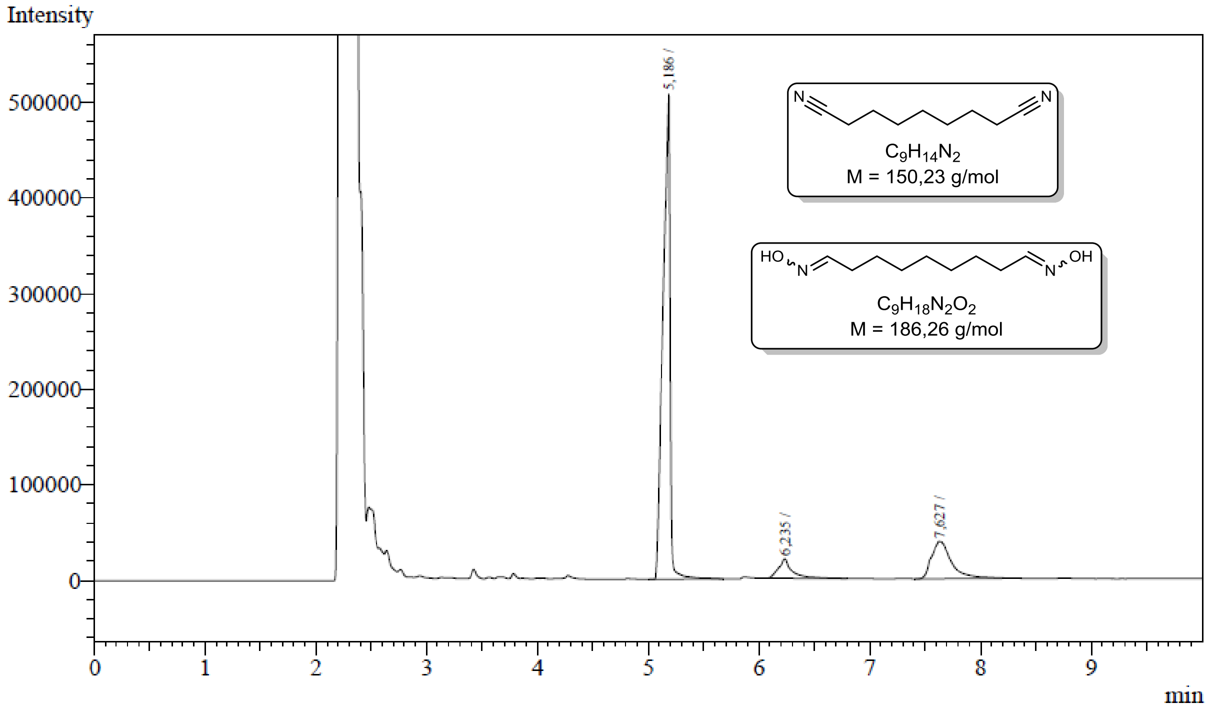

Supplementary Figure 51. GC-chromatogram of nonanedinitrile (2g) and nonanedial dioxime (1g).

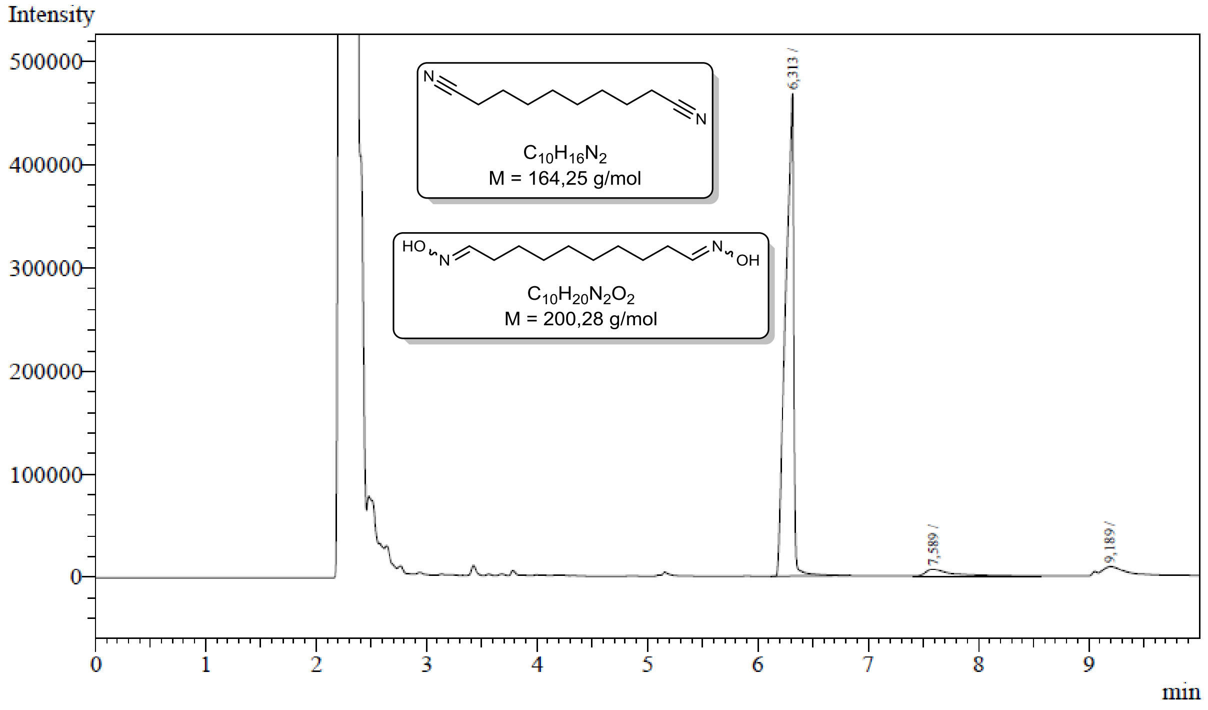

Supplementary Figure 52. GC-chromatogram of decanedinitrile (2h) and decanedial dioxime (1h).

Supplementary Tables

Supplementary Table 1. Activity values for OxdA and OxdB whole-cell catalysts in mU/mg_{BWW}. The activity values were determined according to the protocol described in the Supplementary methods. Each activity value was determined in triplicate and the standard deviation is given as error value.

Substrate[a]/ Conc.	3.0 mM	6.25 mM	12.5 mM	25 mM	50 mM	75 mM
C4 (OxdA)	n.d.	3.3±0.1	4.7±1.3	10.2±0.4	12.3±1.2	15.1[b]±0.5
C5 (OxdA)	2.7±0.1	6.6±0.0	9.7±0.1	13.0±0.2	16.9[b]±0.2	15.6[b]±0.1
C6 (OxdA)	27.8±0.9	34.6±0.3	41.4±0.4	40.4[b]±3.2	38.7[b]±0.4	45.6[b]±5.0
C7 (OxdA)	32.7±0.3	42.4±0.4	46.3[b]±0.3	45.9[b]±0.3	29.8[b]±0.3	24.2[b]±0.1
C8 (OxdA)	26.6±0.2	35.4[b]±0.8	50.1[b]±0.2	39.6[b]±0.2	28.3[b]±0.1	17.5[b]±0.3
C9 (OxdA)	24.8±0.7	36.1[b]±0.3	50.6[b]±0.7	40.9[b]±0.4	32.0[b]±0.0	23.6[b]±0.5
C10 (OxdA)	6.1±0.1	12.3[b]±0.3	21.7[b]±0.2	29.6[b]±0.1	41.7[b]±0.1	49.4[b]±1.7
C4 (OxdB)	15.2±0.4	28.1±0.9	37.3±0.5	33.6±0.8	18.5±0.1	16.6[b]±1.6
C5 (OxdB)	4.9±0.1	7.9±0.0	10.1±0.1	12.3±0.4	15.0[b]±0.0	15.0[b]±0.7
C6 (OxdB)	51.5±0.2	91.8±0.4	145±0.3	169[b]±0.9	151[b]±0.6	114[b]±0.1
C7 (OxdB)	61.4±0.3	62.0±0.1	151[b]±0.9	129[b]±0.7	117[b]±0.1	139[b]±0.2
C8 (OxdB)	25.1±0.5	38.9[b]±0.5	54.5[b]±0.3	39.2[b]±0.1	30.8[b]±0.2	29.6[b]±0.1
C9 (OxdB)	28.7±0.4	43.9[b]±0.6	70.3[b]±0.1	62.1[b]±0.3	58.2[b]±0.2	67.2[b]±0.5
C10 (OxdB)	7.0±0.2	9.1[b]±0.1	13.5[b]±0.0	17.7[b]±0.2	22.9[b]±0.1	27.9[b]±0.5

a) no conversion detected for the C3-dioxime; b) partial precipitation of the substrate.

Supplementary Table 2: Used plasmids for the transformation of *E. coli*. The plasmid names, source organism of the Oxd, abbreviation of the Oxd and the provider are given in the table. For the plasmid cards see Supplementary Figures and for the base sequences of the Oxds Supplementary Methods.

Entry	vector/plasmid	Source organism	Oxd	provider	resistance
1	pET28_OxdA(C)	*Pseudomonas chlororaphis B23*	OxdA	Asano group	Kanamycin
2	pUC18_OxdB	*Bacillus sp. OxB-1*	OxdB	Asano group	Carbenicillin
3	pET28_OxdFG(N)	*Fusarium graminearum*	OxdFG	Thermo Fisher Scientific	Kanamycin
4	pET28_OxdRE(N)	*Rhodococcus erythropolis*	OxdRE	Thermo Fisher Scientific	Kanamycin
5	pET28_OxdRG(N)	*Rhodococcus globerulus A-4*	OxdRG	Thermo Fisher Scientific	Kanamycin

Supplementary Table 3. Preparative scale synthesis with 10-100 g/L substrate concentration. OxdA and OxdB were utilized as whole-cell catalysts and the isolated yield is described after aqueous extraction of adiponitrile.

**Entry**	**Oxd**	**Substrate conc. [g/L]**	**Biomass [g_{BWW}][a]**	**Time [h]**	**Conv. [%]**	**Yield [%]**
1	OxdA/ 20% DMSO	10	0.58 (23 U)	96	>99	75 (608 mg)
2	OxdA	10	1.16 (46 U)	64	>99	59 (480 mg)
3	OxdB/ 20% DMSO	10	0.51 (57 U)	18	>99	55 (446 mg)
4	OxdB	10	0.51 (57 U)	15	>99	70 (570 mg)
5	OxdB /20% DMSO	50	1.50 (171 U)	87	>99	67 (2.47 g)
6	OxdB	50	1.50 (171 U)	22	>99	80 (2.91 g)
7[b]	OxdB	50	24.0 (6000 U)	27	>99	62 (23.1 g)
8[c]	OxdB /20% DMSO	100	0.75 (86 U)	41	70	63 (1.18 g)
9	OxdB	100	4.00 (456 U)	41	75	63 (4.78 g)

[a] BWW = Bio wet weight, U = Unit, defined as µmol/min produced product; [b] 1000 mL reaction volume; [c] 25 mL reaction scale.

Supplementary Methods

All compounds were purchased by Acros Organics, Sigma-Aldrich, TCI Chemicals, VWR Chemicals and Merck. All compounds were used as received. 2-Methyltetrahydrofuran (containing BHT as inhibitor) was distilled prior to use for extraction of the C_7-C_{10} dioximes and dinitriles to avoid overlap of the signals in GC analysis.

NMR spectra were recorded on a Bruker Avance III 500 at a frequence of 500 MHz (^{1}H) or 125 MHz (^{13}C). The chemical shift δ is given in ppm and referenced to the corresponding solvent signal ($CDCl_3$ or DMSO-d6). Coupling constants are given in Hz.

CHN analysis was conducted by the CHN measurement service of the Bielefeld University.

IR spectra were measured on a Nicolet 380 of the Thermo Electron Corporation.

Nano-ESI mass spectra were recorded using an Esquire 3000 ion trap mass spectrometer (Bruker Daltonik GmbH, Bremen, Germany) equipped with a standard nano-ESI source.

High resolution mass spectra are recorded using an Agilent 6220 time-of-flight mass spectrometer (Agilent Technologies, Santa Clara, CA, USA) in extended dynamic range mode equipped with a Dual-ESI source, operating with a spray voltage of 2.5 kV. Nitrogen served both as the nebulizer gas and the dry gas. Nitrogen was generated by a nitrogen generator NGM 11. Samples are introduced with a 1200 HPLC system consisting of an autosampler, degasser, binary pump, column oven and diode array detector (Agilent Technologies, Santa Clara, CA, USA) using a C18 Hypersil Gold column (length: 50 mm, diameter: 2.1 mm, particle size: 1,9 µm) with a short gradient (in 4 min from 0% B to 98% B, back to 0% B in 0.2 min, total run time 7.5 min) at a flow rate of 250 µL/min and column oven temperature of 40°C. HPLC solvent A consists of 94.9% water, 5% acetonitrile and 0.1% formic acid, solvent B of 5% water, 94.9% acetonitrile and 0.1% formic acid.

The mass axis was externally calibrated with ESI-L Tuning Mix (Agilent Technologies, Santa Clara, CA, USA) as calibration standard.

Conversion of the biotransformations was determined by GC measurements in comparison to a calibration curve. GC-chromatograms were recorded on a Shimadzu GC-2010 using the column Phenomenex ZB-5MSi with different temperature programs and H_2 as carrier gas.

E.coli BL21-CodonPlus(DE3)-RIL cells were transformed with the corresponding plasmid containing the gene for each of the aldoxime dehydratases and stored at -80 °C as cryo culture in glycerol prior to use.

The gene for the aldoxime dehydratase from Bacillus sp. OxB-1 (OxdB) was located on a pUC 18 vector.

The genes for the aldoxime dehydratases from Pseudomonas chlororaphis (OxdA), Fusarium graminearum (OxdFG), Rhodococcus erythropolis (OxdRE) and Rhodococcus globerulus (OxdRG) were purchased by GeneArt (Thermo Scientific) or provided by the Asano group in their codon optimized form, located on pET28 plasmids with an sixfold N- or C-terminal His-Tag.

Base sequence sequence (codon-optimized for *E. coli*)[7] of the Aldoxime dehydratase from *Pseudomonas chlororaphis* B23 (OxdA(C_{His6}))

ATGGAAAGCGCAATTGATACCCATCTGAAATGTCCGCGTACCCTGAGCCGTCGT
GTTCCGGAAGAATATCAGCCTCCGTTTCCGATGTGGGTTGCACGTGCCGATGAA
CAGCTGCAGCAGGTTGTTATGGGTTATCTGGGTGTTCAGTATCGTGGTGAAGCA
CAGCGTGAAGCAGCACTGCAGGCAATGCGTCATATTGTTAGCAGCTTTAGCCTG
CCGGATGGTCCGCAGACCCATGATCTGACCCATCATACCGATAGCAGCGGTTTT
GATAATCTGATGGTTGTGGGTTATTGGAAAGATCCGGCAGCACATTGTCGTTGG
CTGCGTAGTGCCGAAGTTAATGATTGGTGGACCAGCCAGGATCGTCTGGGTGA
AGGTCTGGGTTATTTTCGTGAAATTAGCGCACCGCGTGCAGAACAGTTTGAAAC
CCTGTATGCATTTCAGGATAATCTGCCTGGTGTTGGTGCAGTTATGGATAGCAC
CAGCGGTGAAATTGAAGAACATGGTTATTGGGGTAGCATGCGTGATCGTTTTCC
GATTAGCCAGACCGATTGGATGAAACCGACCAATGAACTGCAGGTTGTTGCCG
GTGATCCGGCAAAAGGTGGTCGTGTTGTTATTATGGGTCATGATAACATTGCAC
TGATTCGTAGCGGTCAGGATTGGGCAGATGCAGAAGCAGAAGAACGTAGCCTG
TATCTGGATGAAATTCTGCCGACCCTGCAGGATGGTATGGATTTTCTGCGTGAT

AATGGTCAGCCGCTGGGTTGTTATAGCAATCGTTTTGTTCGTAATATCGATCTGG
ATGGCAATTTTCTGGATGTGAGCTATAACATTGGTCATTGGCGTAGCCTGGAAA
AACTGGAACGTTGGGCAGAAAGCCATCCGACCCATCTGCGTATTTTTGTTACCT
TTTTTCGTGTTGCAGCCGGTCTGAAAAAACTGCGTCTGTATCATGAAGTTAGCGT
GAGTGATGCAAAAAGCCAGGTGTTTGAATATATCAACTGTCATCCGCATACCGG
CATGCTGCGTGATGCAGTTGTTGCACCGACCAAGCTTGCGGCCGCACTCGAGC
ACCACCACCACCACCACTGA

Base sequence (changed the start codon from TTG to ATG)[6] of Aldoxime dehydratase from *Bacillus* sp. strain OxB-1 (OxdB)

ATGAAAAATATGCCGGAAAATCACAATCCACAAGCGAATGCCTGGACTGCCGAA
TTTCCTCCTGAAATGAGCTATGTAGTATTTGCGCAGATTGGGATTCAAAGCAAGT
CTTTGGATCACGCAGCGGAACATTTGGGAATGATGAAAAAGAGTTTCGATTTGC
GGACAGGCCCCAAACATGTGGATCGAGCCTTGCATCAAGGAGCCGATGGATAC
CAAGATTCCATCTTTTTAGCCTACTGGGATGAGCCTGAAACATTTAAATCATGGG
TTGCGGATCCTGAAGTACAAAAGTGGTGGTCGGGTAAAAAAATCGATGAAAATA
GTCCAATCGGGTATTGGAGTGAGGTAACGACCATTCCGATTGATCACTTTGAGA
CTCTTCATTCCGGAGAAAATTACGATAATGGGGTTTCACACTTTGTACCGATCAA
GCATACAGAAGTCCATGAATATTGGGGAGCAATGCGCGACCGCATGCCGGTGT
CTGCCAGTAGTGATTTGGAAAGCCCCCTTGGCCTTCAATTACCGGAACCCATTG
TCCGGGAGTCTTTCGGAAAACGGCTAAAAGTCACGGCGCCGGATAATATTTGCT
TGATTCGAACCGCTCAAAATTGGTCTAAATGTGGTAGCGGGGAAAGGGAAACGT
ATATAGGACTAGTGGAACCGACCCTCATAAAAGCGAATACGTTTCTTCGTGAAAA
TGCTAGTGAAACAGGCTGTATTAGTTCAAAATTAGTCTATGAACAGACCCATGAC
GGCGAAATAGTAGATAAATCATGTGTCATCGGATATTATCTCTCCATGGGGCATC
TTGAACGCTGGACGCATGATCATCCAACACATAAAGCGATCTACGGAACCTTTT
ATGAGATGTTGAAAAGGCATGATTTTAAGACCGAACTTGCTTTATGGCACGAGG
TTTCGGTGCTTCAATCCAAAGATATCGAGCTTATCTATGTCAACTGCCATCCGAG
TACTGGATTTCTTCCATTCTTTGAAGTGACAGAAATTCAAGAGCCTTTACTGAAA
AGCCCTAGCGTCAGGATCCAGTGA

Base sequence (codon-optimized for *E. coli*) of the Aldoxime dehydratase from *Fusarium graminearum* MAFF305135 (OxdFG(N_{His6}))

ATGGGCAGCAGCCATCATCATCATCATCACAGCAGCGGCCTGGTGCCGCGCGG
CAGCCATATGCTGCGTAGCCGTTTTCCGGCAAGCCATCATTTCACCGTTAGCGT
TTTTGGTTGTCAGTATCATAGCGAAGCACCGAGCGTTGAAAAAACCGAACTGAT
TGGTCGTTTCGATAAACTGATTGATAGCGCAGCAATTCATGTGGAACATCTGGA
ACAGAATGATGTGCCGAGCAAAATTTGGATGAGCTATTGGGAAAGTCCGCAGAA
ATTCAAACAGTGGTGGGAAAAAGATGATACCGCAAGCTTTTGGGCAAGCCTGCC
GGATGATGCAGGTTTTTGGCGTGAAACCTTTAGCCTGCCTGCAACCCGTGCAAT
GTATGAAGGCACCGGTAAAGATGCCTATGGTTTTGGTCATTGTGGTAGCCTGAT
TCCGCTGACCACCAAAACCGGCTATTGGGGTGCATATCGTAGCCGTATGACACC
GGATTTTGAAGGTGATACCTTTTCAAGCCCGATTCCGACCTATGCAGATCAGAG
CGTTCCGGCAGATAAAATTCGTCCGGGTCGTGTTCGTATTACCGATTTTCCGGA
TAATCTGTGCATGGTTGTTGAAGGTCAGCATTATGCAGATATGGGTGAACGTGA
ACGCGAATATTGGAACGAAAATTTTGATGGTCTGACGAAACAGTGGGTTACCAA
TGTTGTTACCGCAGGTCATGAACAGGGTATGGTTATTGCACGTGCCTGTCATGG
TTTTGCCGGTGAAAAAAAACTGGGTGCAACCAATGGTCCGGTGAATGGTATTTT
TCCGGGTCTGGATTATGTTCATCAGGCACAGATTCTGATTTGGCAGGATATTAG
CAAAATGGAACATATCGGTCGTTATGATCAGACCCATGTTAAACTGCGTCGCGA
TTTTATGAAAGCCTATGGTCCGGGTGGTGAAATGGAAGGTGGTGATCTGCTGCT
GTGGGTTGATCTGGGTATTCTGAAAAAAGACGAAATCGATGCCGAATATGTGGG
TTGCTATGAAAGTACCGGTTTTCTGAAACTGGATAAAGGCCAGTTTTTCAAAGTT
GAAAGCACCGCAGGTAGCAAACTGCCGAGCTTTTTTGATGAACCGATTGAAAGC
AAACCGATCGAATGGTAA

Base sequence (codon-optimized for *E. coli*) of the Aldoxime dehydratase from *Rhodococcss erythropolis* (*Rhodococcss* sp. N-771, OxdRE(N_{His6}))

ATGGGCAGCAGCCATCATCATCATCATCACAGCAGCGGCCTGGTGCCGCGCGG
CAGCCATATGGAAAGCGCAATTGGTGAACATCTGCAGTGTCCGCGTACCCTGAC
CCGTCGTGTTCCGGATACCTATACCCCTCCGTTTCCGATGTGGGTTGGTCGTGC
AGATGATGCACTGCAGCAGGTTGTTATGGGTTATCTGGGTGTTCAGTTTCGTGA

TGAAGATCAGCGTCCGGCAGCACTGCAGGCAATGCGTGATATTGTTGCAGGTTT
TGATCTGCCGGATGGTCCGGCACATCATGATCTGACCCATCATATTGATAATCA
GGGCTATGAAAACCTGATTGTGGTGGGTTATTGGAAAGATGTTAGCAGCCAGCA
TCGTTGGAGCACCAGCACCCCGATTGCAAGTTGGTGGGAAAGCGAAGATCGTC
TGAGTGATGGTCTGGGTTTTTTTCGTGAAATTGTGGCACCGCGTGCAGAACAGT
TTGAAACCCTGTATGCATTTCAAGAAGATCTGCCTGGCGTTGGTGCAGTTATGG
ATGGTATTAGCGGTGAAATTAACGAACATGGTTATTGGGGTAGCATGCGTGAAC
GTTTTCCGATTAGCCAGACCGATTGGATGCAGGCAAGCGGTGAACTGCGTGTTA
TTGCCGGTGATCCGGCAGTTGGTGGTCGTGTTGTTGTTCGTGGTCATGATAACA
TTGCACTGATTCGTAGCGGTCAGGATTGGGCAGATGCCGAAGCAGATGAACGT
AGCCTGTATCTGGATGAAATTCTGCCGACCCTGCAGAGCGGTATGGATTTTCTG
CGTGATAATGGTCCTGCAGTTGGTTGTTATAGCAATCGTTTTGTGCGCAACATTG
ATATCGATGGCAATTTTCTGGATCTGAGCTATAACATTGGTCATTGGGCAAGCCT
GGATCAGCTGGAACGTTGGAGCGAAAGCCATCCGACCCATCTGCGTATTTTTAC
CACCTTTTTTCGCGTTGCAGCCGGTCTGAGCAAACTGCGTCTGTATCATGAAGT
TAGCGTTTTTGATGCAGCAGATCAGCTGTATGAATACATTAATTGTCATCCGGGT
ACAGGTATGCTGCGTGATGCAGTTACCATTGCAGAACATTAA

Base sequence (codon-optimized for *E. coli*) of the Aldoxime dehydratase from *Rhodococcss globerulus* A-4 (OxdRG(N_{His6}))

ATGGGCAGCAGCCATCATCATCATCATCACAGCAGCGGCCTGGTGCCGCGCGG
CAGCCATATGGAAAGCGCAATTGGTGAACATCTGCAGTGTCCGCGTACCCTGAC
CCGTCGTGTTCCGGATACCTATACCCCTCCGTTTCCGATGTGGGTTGGTCGTGC
AGATGATACCCTGCATCAGGTTGTTATGGGTTATCTGGGTGTTCAGTTTCGTGG
TGAAGATCAGCGTCCGGCAGCACTGCGTGCAATGCGTGATATTGTTGCAGGTTT
TGATCTGCCGGATGGTCCGGCACATCATGATCTGACCCATCATATTGATAATCA
GGGCTATGAAAACCTGATTGTGGTGGGTTATTGGAAAGATGTTAGCAGCCAGCA
TCGTTGGAGCACCAGCCCTCCGGTTAGCAGTTGGTGGGAAAGCGAAGATCGTC
TGAGTGATGGTCTGGGTTTTTTTCGTGAAATTGTGGCACCGCGTGCAGAACAGT
TTGAAACCCTGTATGCATTTCAGGATGATCTGCCTGGTGTTGGTGCAGTTATGG
ATGGTGTTAGCGGTGAAATTAATGAACATGGTTATTGGGGTAGCATGCGTGAAC
GTTTTCCGATTAGCCAGACCGATTGGATGCAGGCAAGCGGTGAACTGCGTGTT

GTTGCCGGTGATCCGGCAGTTGGCGGTCGTGTTGTGGTTCGTGGTCATGATAA
CATTGCACTGATTCGTAGCGGTCAGGATTGGGCAGATGCCGAAGCAGATGAAC
GTAGCCTGTATCTGGATGAAATTCTGCCGACCCTGCAGAGCGGTATGGATTTTC
TGCGTGATAATGGTCCTGCAGTTGGTTGTTATAGCAATCGTTTTGTGCGCAACAT
TGATATCGATGGCAATTTTCTGGATCTGAGCTATAACATTGGTCATTGGGCAAGC
CTGGATCAGCTGGAACGTTGGAGCGAAAGCCATCCGACCCATCTGCGTATTTTT
ACCACCTTTTTTCGCGTTGCAGAAGGTCTGAGCAAACTGCGTCTGTATCATGAA
GTTAGCGTTTTTGATGCAGCAGATCAGCTGTATGAATACATTAATTGTCATCCGG
GTACAGGTATGCTGCGTGATGCAGTTATTACCGCAGAACATTAA

Expression and storage of the aldoxime dehydratases (Oxds)

Pre-culture: 10 mL LB-medium in a 100 mL Erlenmeyer flask containing 50 µg/mL Kanamycin or 100 µg/mL Carbenicillin and 34 µg/mL Chloramphenicol were inoculated with an *E. coli* clone and incubated at 37 °C and 180 rpm for 24 hours.

Main culture: 100-450 mL (in 100-500 mL Erlenmeyer flasks) Auto-induction medium (Recipe for 1L: 890 mL TB-Medium (pH = 7.0), 10 mL 50 g/L Glucose and 100 mL 20 g/L Lactose) was inoculated with 1.0 Vol.-% of the pre-culture, followed by addition of 50 µg/mL Kanamycin or 100 µg/mL Carbenicillin and 34 µg/mL Chloramphenicol. The culture was incubated for one hour at 37 °C and 180 rpm, followed by incubation at 15 °C (OxdA, OxdFG, OxdRE, OxdRG) or 30 °C (OxdB) for 72 hours.

The cells were harvested by centrifugation (4000 g, 4 °C, 15 min). The supernatant was discarded and the pellets were washed twice with 50 mM potassium phosphate buffer (pH = 7.0). After repeated centrifugation (4000 g, 4 °C, 15 min) and weighing of the pellets (bio wet weight, BWW), they were suspended in 50 mM potassium phosphate buffer (50-fold concentrated, pH = 7.0), optionally overlaid with argon and stored at 4 °C as resting cell suspension (typically 25-35 wt%).

Overexpression of the aldoxime dehydratases was checked via SDS-PAGE. A 25 wt% cell suspension was disrupted by ultrasound sonification (*Sonoplus HD 2070*, 5 x 2 min, 10% power) on ice. Insoluble cell components were separated via centrifugation (21500 g, 4 °C, 15 min). 10 µL of the diluted crude extract (1 mg protein/mL) were pipetted into the collection gel and analyzed via SDS-PAGE (12% separation gel).

Influence of water soluble co-solvents on the activity of Oxds (short term studies)

Into a 1.5 mL micro reaction tube with shaking of 1400 rpm at 30 °C were given 400/350/300 µL 50 mM KPB (pH = 7.0) and 50 µL resting cell suspension. Afterwards a water-soluble co-solvent (37.5/87.5/137.5 µL) was added. The suspension was incubated for 20 minutes and the assay was started by addition of 12.5 µL phenylacetaldehyde oxime (400 mM, final concentration of 10 mM), dissolved in the corresponding co-solvent. 100 µL 0.1 M HCl and 400 µL Acetonitrile were added after 60 seconds to quench the reaction. 800 µL of the supernatant after centrifugation (15000 g, 4 °C, 5 min) were transferred into a vial and measured on RP-HPLC for conversion according to a calibration curve. The relative activity was determined by comparison with a reference experiment, in which no co-solvent was added during the incubation time. Each assay was conducted in duplicate and analyzed via RP-HPLC in duplicate.

Influence of water soluble co-solvents on the activity of OxdA und OxdB (long term studies)

Into a 1.5 mL micro reaction tube with shaking of 1400 rpm at 30 °C were given 350/300 µL 50 mM KPB (pH = 7.0) and 100 µL resting cell suspension. Afterwards were added 37.5/87.5 µL of MeOH/DMSO (for OxdA) or 37.5/87.5 µL EtOH/iPrOH/DMSO/DMC (for OxdB). The suspension was incubated for 15/30/60/120/180 minutes and the assay was started by addition of 12.5 µL substrate (400 mM, final concentration of 10 mM), dissolved in the corresponding co-solvent. 100 µL 0.1 M HCl and 400 µL Acetonitrile were added after 60 seconds to quench the reaction. 800 µL of the supernatant after centrifugation (15000 g, 4 °C, 5 min) were transferred into a vial and measured on RP-HPLC for conversion. The relative activity was determined by comparison with a reference experiment, in which no co-solvent was added during the incubation time.

Activity assay for the biocatalytic dehydration of dioximes by OxdA and OxdB

800 µL of a whole cell catalyst suspension in 50 mM KPB, pH = 7.0 (2.0 mg_{BWW}, containing OxdA or OxdB) was mixed with 0-192 µL DMSO and incubated for five minutes at 30 °C and vigorous shaking. The assay was started by adding 8-200 µL of a 375 mM stock solution of the substrate in DMSO. The assay was stopped after 15 minutes by addition of 1.0 mL 2-Me-THF and immediate extraction of the substrate by vortexing for 60 seconds. After centrifugation (4 °C, 15000 g, 5 min), the supernatant (800 µL) was transferred into a GC-vial and analyzed by gas chromatography. The conversion was determined by calibration curves, including a correction factor accounting for incomplete extraction of the substrate and product. Each assay was analyzed via GC in duplicate or triplicate.

Synthesis of adipaldehyde by oxidation of *trans*-1,2-Cyclohexanediol

Silica (275 g) was suspended in 900 mL CH_2Cl_2 in a 2 L three-necked flask equipped with two 500 mL dropping funnels. Afterwards, the apparatus was flushed with argon and a solution of $NaIO_4$ (38.0 g, 178 mmol) in 250 mL H_2O was added dropwise. *trans*-1,2-Cyclohexanediol (15.8 g, 136 mmol) was dissolved in 500 mL CH_2Cl_2 and also added dropwise to the suspension. After stirring for 24 hours at room temperature, the solid was filtered off and washed with CH_2Cl_2. The solvent of the filtrate was evaporated *in vacuo* to yield Adipaldehyde as pale yellow liquid.

Yield: 14.4 g, 93%.

^{1}H-NMR (500 MHz, $CDCl_3$): δ [ppm] = 9.75 (m, 2H, $CH_2CH_2C\mathit{HO}$), 2.46 (m, 4H, $CH_2C\mathit{H_2}CHO$), 1.65 (m, 4H, $C\mathit{H_2}CH_2CHO$).

^{13}C-NMR (125 MHz, $CDCl_3$): δ [ppm] = 202.03, 43.66, 21.57.

The analytical data corresponds with the literature.[8]

Synthesis of malonoaldehyde dioxime via condensation of 1,1,3,3-tetramethoxypropane with hydroxylamine hydrochloride

1,1,3,3-tetramethoxypropane (5.00 mL, 30.4 mmol) was given to a solution of hydroxylamine hydrochloride (6.34 g, 91.2 mmol) in 20 mL H_2O. The solution was degassed under vacuum, followed by flushing with argon to establish an inert atmosphere. Heating to 40 °C resulted in a yellow solution, into which sodium carbonate (4.83 g, 45.6 mmol) was added. The suspension was cooled to 0 °C for 24 hours. The solid was filtered off, washed with water and dried *in vacuo*. The product was obtained as colorless solid. *E*/*Z*-ratio (including both oxime groups) was 1:99 according to ^{1}H-NMR.

Yield: 744 mg, 28%.

^{1}H-NMR (500 MHz, DMSO): δ [ppm] = 11.03 (s, 2H, $CH_2(CHNO\mathit{H})_2$), 6.78 (t, 2H, ^{3}J = 5.3 Hz, $CH_2(C\mathit{H}NOH)_2$), 3.19 (t, 2H, ^{3}J = 5.3 Hz, $C\mathit{H_2}(CHNOH)_2$).

^{13}C-NMR (125 MHz, DMSO): δ [ppm] = 144.94, 22.63.

GC (FID): Phenomenex ZB-5MSi, 1.03 ml/min (H_2), Inj. Temp.: 300 °C, Det. Temp.: 350 °C; 100 °C -> 125 °C (5 °C/min), 125 °C -> 205 °C (40 °C/min); $R_{t\ dinitrile}$ = 2.36 min, $R_{t\ dioxime}$ = 4.04 min.

HRMS (ESI): calcd for $C_3H_7N_2O_2$ $[M+H]^+$: 103.0502, found: 103.0508.

IR (neat) [cm^{-1}]: 3081, 3041, 2809, 1660, 1434, 1399, 1320, 1252, 946, 927, 860, 782, 746, 676.

Synthesis of succinaldehyde dioxime via condensation of succinaldehyde Bis(dimethylacetal) with hydroxylamine hydrochloride

Succinaldehyde Bis(dimethylacetal) (2.65 mL, 15.0 mmol) was given to a solution of hydroxylamine hydrochloride (3.13 g, 45.0 mmol) in 10 mL H_2O. The solution was degassed under vacuum, followed by flushing with argon to establish an inert atmosphere. The phase separation disappeared at room temperature, upon which

sodium carbonate (2.38 g, 22.5 mmol) was added. The suspension was cooled to 0 °C for 24 hours. The solid was filtered off, washed with water and dried *in vacuo*. The product was obtained as colorless solid. *E*/*Z*-ratio (including both oxime groups) was 8:92 according to ^{1}H-NMR.

Yield: 1.12 g, 64%.

^{1}H-NMR (500 MHz, DMSO): δ [ppm] = 10.86 (s, 2H, $CH_2(CHNO\mathit{H})_2$), 6.66 (m, 2H, $CH_2(C\mathit{H}NOH)_2$), 2.38 (m, 2H, $C\mathit{H_2}(CHNOH)_2$).

^{13}C-NMR (125 MHz, DMSO): δ [ppm] = 149.37, 21.55.

GC (FID): Phenomenex ZB-5MSi, 1.03 ml/min (H_2), Inj. Temp.: 300 °C, Det. Temp.: 350 °C; 100 °C -> 135 °C (5 °C/min), 135 °C -> 215 °C (40 °C/min); $R_{t\ dinitrile}$ = 3.04 min, $R_{t\ dioxime}$ = 5.30 min.

HRMS (ESI): calcd for $C_4H_9N_2O_2$ $[M+H]^+$: 117.0659, found: 145.0669.

IR (neat) [cm^{-1}]: 3085, 3043, 2868, 2810, 1671, 1448, 1420, 1328, 1234, 1037, 935, 918, 879, 807, 774, 753, 717.

Synthesis of glutaraldehyde dioxime via condensation of glutaraldehyde with hydroxylamine hydrochloride

A 50wt% solution of Glutaraldehyde (2.00 mL, 11.2 mmol) was given to a solution of hydroxylamine hydrochloride (2.34 g, 33.6 mmol) and sodium carbonate (3.56 g, 33.6 mmol) in 50 mL H_2O. After 30 min the solution turned into a colorless suspension. After two hours complete conversion was achieved according to TLC. The purity of the crude product after extraction with ethyl acetate was satisfactory for further syntheses. The *E*/*Z*-ratio (including both oxime groups) was 8:92 according to ^{1}H-NMR.

Yield: 960 mg, 66%.

^{1}H-NMR (500 MHz, DMSO): δ [ppm] = 10.77 (s, 2H, *Z*, CHNO*H*), 10.41 (s, 2H, *E*, CHNO*H*), 7.30 (t, 2H, *E*, ^3J = 5.8 Hz, C*H*NOH), 6.65 (t, 2H, *Z*, ^3J = 5.4 Hz, C*H*NOH), 2.23 (m, 4H, *Z*, CH_2CHNOH), 2.11 (m, 4H, *E*, CH_2CHNOH), 1.55 (m, 2H, *E*/*Z*, CH_2).

^{13}C-NMR (125 MHz, DMSO): δ [ppm] = 149.81 (*Z*), 149.11 (*E*), 24.43 (*Z*)., 24.16 (*E*), 22.94 (*E*), 22.38 (*Z*).

GC (FID): Phenomenex ZB-5MSi, 0.87 ml/min (H_2), Inj. Temp.: 300 °C, Det. Temp.: 350 °C; 140 °C -> 190 °C (5 °C/min); $R_{t\ dinitrile}$ = 2.61 min, $R_{t\ dioxime}$ = 3.63 min.

The analytical data corresponds with the literature.[9]

Synthesis of adipaldehyde dioxime via condensation of adipaldehyde with hydroxylamine hydrochloride

Adipaldehyde (14.1 g, 124 mmol) was dissolved in 100 mL H2O and 25 mL methanol. Hydroxylamine hydrochloride (25.9 g, 372 mmol) was added and afterwards sodium carbonate (19.7 g, 186 mmol). The suspension was stirred at room temperature until complete conversion according to TLC was achieved. The solid was filtered off, washed with water and dried *in vacuo*. The product was obtained as colorless solid. *E*/*Z*-ratio (including both oxime groups) was 7:93 according to ^{1}H-NMR.

Yield: 12.6 g, 70%.

^{1}H-NMR (500 MHz, DMSO): δ [ppm] = 10.73 (s, 2H, *Z*, CHNO*H*), 10.36 (s, 2H, *E*, CHNO*H*), 7.29 (t, 2H, *E*, ^3J = 5.9 Hz, C*H*NOH), 6.63 (t, 2H, *Z*, ^3J = 5.3 Hz, C*H*NOH), 2.24 (m, 4H, *Z*, CH_2CHNOH), 2.10 (m, 4H, *E*, CH_2CHNOH), 1.42 (m, 4H, *E*/*Z*, CH_2).

^{13}C-NMR (125 MHz, DMSO): δ [ppm] = 150.16, 150.14, 149.39, 149.37, 28.72, 28.71, 26.03, 25.71, 25.47, 25.16, 24.33.

GC (FID): Phenomenex ZB-5MSi, 0.87 ml/min (H_2), Inj. Temp.: 300 °C, Det. Temp.: 350 °C; 140 °C -> 190 °C (5 °C/min); $R_{t\ dinitrile}$ = 3.08 min, $R_{t\ dioxime}$ = 4.52 min.

HRMS (ESI): calcd for $C_6H_{13}N_2O_2$ $[M+H]^+$: 145.0972, found: 145.0972.

IR (neat) [cm^{-1}]: 3182, 3084, 3039, 2934, 2865, 2810, 1664, 1451, 1415, 1345, 1322, 1058, 924, 826, 803, 733, 721, 705.

Synthesis of heptanedial dioxime via condensation of heptanedial with hydroxylamine hydrochloride

Hydroxylamine hydrochloride (1.02 g, 14.7 mmol) was dissolved in 10 mL H_2O and 2.5 mL methanol. Heptanedial (630 mg, 4.92 mmol) was added, followed by sodium carbonate (782 mg, 7.38 mmol). The suspension was stirred at room temperature until complete conversion according to TLC was achieved. The solid was filtered off, washed with water and dried *in vacuo*. The product was obtained as colorless solid. *E*/*Z*-ratio (including both oxime groups) was 14:86 according to ^{1}H-NMR.

Yield: 532 mg, 68% yield.

^{1}H-NMR (500 MHz, DMSO): δ [ppm] = 10.71 (s, 2H, *Z*, CHNO*H*), 10.36 (s, 2H, *E*, CHNO*H*), 7.28 (t, 2H, *E*, ^3J = 5.9 Hz, C*H*NOH), 6.63 (t, 2H, *Z*, ^3J = 5.3 Hz, C*H*NOH), 2.21 (m, 4H, *Z*, CH_2CHNOH), 2.08 (m, 4H, *E*, CH_2CHNOH), 1.41 (m, 4H, *E*/*Z*, C$H_2$$CH_2$CHNOH), 1.29 (m, 2H, *E/Z*, C$H_2$$CH_2$$CH_2$CHNOH).

^{13}C-NMR (125 MHz, DMSO): δ [ppm] = 150.29, 149.49, 28.86, 28.66, 28.37, 25.97, 25.35, 24.45.

GC (FID): Phenomenex ZB-5MSi, 0.87 ml/min (H_2), Inj. Temp.: 300 °C, Det. Temp.: 350 °C; 140 °C -> 190 °C (5 °C/min); $R_{t\ dinitrile}$ = 4.02 min, $R_{t\ dioxime}$ = 4.78+5.95 min.

MS (ESI): m/z = 159.0 $[M+H]^+$.

HRMS (ESI): calcd for $C_7H_{15}N_2O_2$ $[M+H]^+$: 159.1128, found: 159.1131.

IR (neat) [cm^{-1}]: 3180, 3078, 3033, 2928, 2859, 1456, 1438, 1417, 1313, 1059, 920, 886, 814, 769, 722.

Synthesis of octanedial dioxime via condensation of octanedial with hydroxylamine hydrochloride

Hydroxylamine hydrochloride (1.32 g, 19.0 mmol) was dissolved in 16 mL H_2O and 4 mL methanol. Octanedial (900 mg, 6.33 mmol) was added, followed by sodium carbonate (1.01 g, 9.49 mmol). The suspension was stirred at room temperature until complete conversion according to TLC was achieved. The solid was filtered off, washed with water and dried *in vacuo*. The product was obtained as colorless solid. *E*/*Z*-ratio (including both oxime groups) was 40:60 according to ^{1}H-NMR.

Yield: 900 mg, 83% yield.

^{1}H-NMR (500 MHz, DMSO): δ [ppm] = 10.70 (s, 2H, *Z*, CHNO*H*), 10.34 (s, 2H, *E*, CHNO*H*), 7.28 (t, 2H, *E*, ^3J = 5.9 Hz, C*H*NOH), 6.62 (t, 2H, *Z*, ^3J = 5.3 Hz, C*H*NOH), 2.21 (m, 4H, *Z*, CH_2CHNOH), 2.08 (m, 4H, *E*, CH_2CHNOH), 1.40 (m, 4H, *E*/*Z*, C$H_2$$CH_2$CHNOH), 1.28 (m, 4H, *E/Z*, C$H_2$$CH_2$$CH_2$CHNOH).

^{13}C-NMR (125 MHz, DMSO): δ [ppm] = 150.32, 149.53, 28.93, 28.60, 28.29, 26.15, 25.53, 24.52.

GC (FID): Phenomenex ZB-5MSi, 0.87 ml/min (H_2), Inj. Temp.: 300 °C, Det. Temp.: 350 °C; 140 °C -> 190 °C (5 °C/min); $R_{t\ dinitrile}$ = 4.89 min, $R_{t\ dioxime}$ = 5.90+7.37 min.

MS (ESI): m/z = 173.0 $[M+H]^+$.

HRMS (ESI): calcd for $C_8H_{17}N_2O_2$ $[M+H]^+$: 173.1285, found: 173.1284.

IR (neat) [cm^{-1}]: 3177, 3085, 3038, 2925, 2851, 1464, 1450, 1417, 1329, 1071, 918, 861, 813, 737, 719, 711.

Synthesis of nonanedial dioxime via condensation of nonanedial with hydroxylamine hydrochloride

Hydroxylamine hydrochloride (1.55 g, 22.3 mmol) was dissolved in 16 mL H_2O and 4 mL methanol. Nonanedial (1.16 g, 7.42 mmol) was added, followed by sodium carbonate (1.18 g, 11.1 mmol). The suspension was stirred at room temperature until complete conversion according to TLC was achieved. The solid was filtered off, washed with water and dried *in vacuo*. The product was obtained as colorless solid. *E*/*Z*-ratio (including both oxime groups) was 9:91 according to ^{1}H-NMR.

Yield: 1.02 g, 74% yield.

^{1}H-NMR (500 MHz, DMSO): δ [ppm] = 10.71 (s, 2H, *Z*, CHNO*H*), 10.35 (s, 2H, *E*, CHNO*H*), 7.27 (t, 2H, *E*, ^3J = 5.9 Hz, C*H*NOH), 6.61 (t, 2H, *Z*, ^3J = 5.3 Hz, C*H*NOH), 2.20 (m, 4H, *Z*, CH_2CHNOH), 2.07 (m, 4H, *E*, CH_2CHNOH), 1.40 (m, 4H, *E*/*Z*, CH_2CH$_2$CHNOH), 1.27 (m, 6H, *E/Z*, CH_2CH_2CH$_2$CH$_2$CHNOH).

^{13}C-NMR (125 MHz, DMSO): δ [ppm] = 150.30, 149.50, 28.97, 28.80, 28.53, 28.48, 26.25, 25.63, 25.51, 24.55.

GC (FID): Phenomenex ZB-5MSi, 0.87 ml/min (H_2), Inj. Temp.: 300 °C, Det. Temp.: 350 °C; 150 °C -> 200 °C (5 °C/min); $R_{t\ dinitrile}$ = 5.18 min, $R_{t\ dioxime}$ = 6.18+7.61 min.

MS (ESI): m/z = 187.0 $[M+H]^+$.

HRMS (ESI): calcd for $C_9H_{19}N_2O_2$ $[M+H]^+$: 187.1441, found: 187.1446.

IR (neat) [cm^{-1}]: 3194, 3085, 2923, 2848, 1463, 1440, 1416, 1329, 1308, 916, 840, 814, 749, 737, 712.

Synthesis of decanedial dioxime via condensation of decanedial with hydroxylamine hydrochloride

Hydroxylamine hydrochloride (1.72 g, 24.7 mmol) was dissolved in 20 mL H_2O and 5 mL methanol. Decanedial (1.40 g, 8.22 mmol) was added, followed by sodium carbonate (1.31 g, 12.3 mmol). The suspension was stirred at room temperature until complete conversion according to TLC was achieved. The solid was filtered off, washed with water and dried *in vacuo*. The product was obtained as colorless solid. *E*/*Z*-ratio (including both oxime groups) was 43:57 according to ^{1}H-NMR.

Yield: 1.22 g, 74% yield.

^{1}H-NMR (500 MHz, DMSO): δ [ppm] = 10.69 (s, 2H, *Z*, CHNO*H*), 10.33 (s, 2H, *E*, CHNO*H*), 7.28 (t, 2H, *E*, ^3J = 5.9 Hz, C*H*NOH), 6.62 (t, 2H, *Z*, ^3J = 5.3 Hz, C*H*NOH), 2.20 (m, 4H, *Z*, CH_2CHNOH), 2.07 (m, 4H, *E*, CH_2CHNOH), 1.39 (m, 4H, *E*/*Z*, CH_2CH_2CHNOH), 1.26 (m, 8H, *E/Z*, $CH_2CH_2CH_2CH_2$CHNOH).

^{13}C-NMR (125 MHz, DMSO): δ [ppm] = 150.32, 149.51, 28.95, 28.85, 28.69, 28.54, 26.25, 25.63, 24.54.

GC (FID): Phenomenex ZB-5MSi, 0.87 ml/min (H_2), Inj. Temp.: 300 °C, Det. Temp.: 350 °C; 150 °C -> 200 °C (5 °C/min); $R_{t\ dinitrile}$ = 6.32 min, $R_{t\ dioxime}$ = 7.58+9.13 min.

MS (ESI): m/z = 201.1 $[M+H]^+$.

HRMS (ESI): calcd for $C_{10}H_{21}N_2O_2$ $[M+H]^+$: 201.1598, found: 201.1595.

IR (neat) [cm^{-1}]: 3184, 3080, 2923, 2849, 1465, 1446, 1323, 918, 884, 816, 745, 718.

Synthesis of heptanedial by oxidation of 1,7-heptanediol with Bobbitt's salt (4-(Acetylamino)-2,2,6,6-tetramethyl-1-oxo-piperidinium tetrafluoroborate)

The synthesis was carried out according to *Miller et al.*.[10] To a heat dried round bottom flask was added 1,7-heptanediol (1.32 g, 10.0 mmol) and 100 mL dichloromethane under an inert gas atmosphere. After stirring for five minutes at room temperature, silica (2.64 g) and Bobbitt's salt (6.30 g, 21.0 mmol) were added, resulting in a yellow suspension. After stirring for 120 hours, filtration of the slurry through a 3 cm thick pad of silica was conducted. The residue was washed with dichlormethane and the filtrate was freed from the solvent *in vacuo* to yield the heptanedial as pale yellow liquid.

Yield: 650 mg, 51%.

^{1}H-NMR (500 MHz, $CDCl_3$): δ [ppm] = 9.75 (m, 2H, CH_2COH), 2.44 (t, 4H, ^3J = 7.3 Hz, CH_2COH), 1.64 (qi, 4H, ^3J = 7.4 Hz, CH_2CH_2COH), 1.35 (m, 2H, $CH_2CH_2CH_2COH$).

^{13}C-NMR (125 MHz, $CDCl_3$): δ [ppm] = 202.41, 43.72, 28.70, 21.86.

The analytical data corresponds with the literature.[10]

Synthesis of octanedial by oxidation of 1,8-octanediol with Bobbitt's salt (4-(Acetylamino)-2,2,6,6-tetramethyl-1-oxo-piperidinium tetrafluoroborate)

The synthesis was carried out according to *Miller et al.*.[10] To a heat dried round bottom flask was added 1,8-octanediol (1.46 g, 10.0 mmol) and 100 mL dichloromethane under an inert gas atmosphere. After stirring for five minutes at room temperature, silica (2.64 g) and Bobbitt's salt (6.30 g, 21.0 mmol) were added, resulting in a yellow suspension. After stirring for 120 hours, filtration of the slurry through a 3 cm thick pad of silica was conducted. The residue was washed with dichlormethane and the filtrate was freed from the solvent *in vacuo* to yield the octanedial as pale yellow liquid.

Yield: 925 mg, 65%.

^{1}H-NMR (500 MHz, $CDCl_3$): δ [ppm] = 9.75 (m, 2H, CH_2COH), 2.42 (t, 4H, ^3J = 7.3 Hz, CH_2COH), 1.62 (qi, 4H, ^3J = 7.0 Hz, CH_2CH_2COH), 1.34 (m, 4H, $CH_2CH_2CH_2COH$).

^{13}**C-NMR** (125 MHz, $CDCl_3$): δ [ppm] = 202.68, 43.87, 28.97, 21.91.

The analytical data corresponds with the literature.[10]

Synthesis of nonanedial by oxidation of 1,9-nonanediol with Bobbitt's salt (4-(Acetylamino)-2,2,6,6-tetramethyl-1-oxo-piperidinium tetrafluoroborate)

The synthesis was carried out according to *Miller et al.*.[10] To a heat dried round bottom flask was added 1,9-nonanediol (1.60 g, 10.0 mmol) and 100 mL dichloromethane under an inert gas atmosphere. After stirring for five minutes at room temperature, silica (2.64 g) and Bobbitt's salt (6.30 g, 21.0 mmol) were added, resulting in a yellow suspension. After stirring for 120 hours, filtration of the slurry through a 3 cm thick pad of silica was conducted. The residue was washed with dichlormethane and the filtrate was freed from the solvent *in vacuo* to yield the nonanedial as pale yellow liquid.

Yield: 1.18 g, 76%.

^{1}**H-NMR** (500 MHz, $CDCl_3$): δ [ppm] = 9.75 (m, 2H, CH_2COH), 2.41 (t, 4H, ^3J = 7.3 Hz, CH_2COH), 1.61 (qi, 4H, ^3J = 7.1 Hz, CH_2CH_2COH), 1.32 (m, 6H, $CH_2CH_2CH_2CH_2COH$).

^{13}**C-NMR** (125 MHz, $CDCl_3$): δ [ppm] = 202.83, 43.95, 29.20, 29.03, 22.06.

The analytical data corresponds with the literature.[10]

Synthesis of decanedial by oxidation of 1,10-decanediol with Bobbitt's salt (4-(Acetylamino)-2,2,6,6-tetramethyl-1-oxo-piperidinium tetrafluoroborate)

The synthesis was carried out according to *Miller et al.*.[10] To a heat dried round bottom flask was added 1,10-decanediol (1.74 g, 10.0 mmol) and 100 mL dichloromethane under an inert gas atmosphere. After stirring for five minutes at room temperature, silica (2.64 g) and Bobbitt's salt (6.30 g, 21.0 mmol) were added, resulting in a yellow suspension. After stirring for 120 hours, filtration of the slurry through a 3 cm thick pad

of silica was conducted. The residue was washed with dichlormethane and the filtrate was freed from the solvent *in vacuo* to yield the decanedial as pale yellow liquid.

Yield: 1.22 g, 72%.

^{1}H-NMR (500 MHz, $CDCl_3$): δ [ppm] = 9.73 (m, 2H, CH_2COH), 2.39 (t, 4H, ^3J = 7.1 Hz, CH_2COH), 1.59 (qi, 4H, ^3J = 6.9 Hz, CH_2CH_2COH), 1.28 (m, 8H, $CH_2CH_2CH_2CH_2COH$).

^{13}C-NMR (125 MHz, $CDCl_3$): δ [ppm] = 202.88, 43.93, 29.19, 29.11, 22.07.

The analytical data corresponds with the literature.[10]

Preparative biotransformations and scale-up

A 100 mL reaction mixture consisting of whole cell catalyst suspension in 50 mM KPB, pH = 7.0 (0.6-4.0 $wt\%_{BWW}$, containing OxdA or OxdB) and the solid Adipaldehyde oxime (1.0-10 g) were mixed in a sealable glass flask. Argon was flushed through the flask and it was sealed afterwards. The mixture was stirred at 180 rpm at 30 °C. In case of using DMSO as a co-solvent, the reaction mixture consisted of 80 mL cell suspension and 20 mL DMSO. An aliquot of 1.0 mL was taken several times to determine the conversion via Gas chromatography (GC). For this, the aliquot was mixed with 1.0 mL 2-Me-THF and extracted for 1 minute. The supernatant was taken off und injected into the GC apparatus. The conversion was determined according to calibration curves.

After complete conversion to adiponitrile, the reaction mixture was extracted three times with MTBE (1:1, v/v). In case of using DMSO as co-solvent, the combined extracts were washed once with brine (1:3, v/v). Subsequently, the extracts were dried over $MgSO_4$, filtered and the solvent was removed *in vacuo* to yield adiponitrile as pale yellow liquid with 98-99% purity. The purity of the product was determined via ^{1}H-NMR- and GC-analysis.

Supplementary References

1. Xie, S.-X., Kato, Y., Komeda, H., Yoshida, S. & Asano, Y. A gene cluster responsible for alkylaldoxime metabolism coexisting with nitrile hydratase and amidase in Rhodococcus globerulus A-4. *Biochemistry* **42,** 12056–12066 (2003).
2. Kato, Y., Yoshida, S., Xie, S.-X. & Asano, Y. Aldoxime dehydratase co-existing with nitrile hydratase and amidase in the iron-type nitrile hydratase-producer Rhodococcus sp. N-771. *Journal of Bioscience and Bioengineering* **97,** 250–259 (2004).
3. Kato, Y. & Asano, Y. Purification and characterization of aldoxime dehydratase of the head blight fungus, Fusarium graminearum. *Bioscience, Biotechnology, and Biochemistry* **69,** 2254–2257 (2005).
4. Kato, Y., Nakamura, K., Sakiyama, H., Mayhew, S.G. & Asano, Y. Novel Heme-Containing Lyase, Phenylacetaldoxime Dehydratase from Bacillus sp. Strain OxB-1. Purification, Characterization, and Molecular Cloning of the Gene. *Biochemistry* **39,** 800–809 (2000).
5. Oinuma, K.-I. *et al.* Novel aldoxime dehydratase involved in carbon-nitrogen triple bond synthesis of Pseudomonas chlororaphis B23. Sequencing, gene expression, purification, and characterization. *The Journal of biological chemistry* **278,** 29600–29608 (2003).
6. Kato, Y. & Asano, Y. High-level expression of a novel FMN-dependent heme-containing lyase, phenylacetaldoxime dehydratase of Bacillus sp. strain OxB-1, in heterologous hosts. *Protein Expression and Purification* **28,** 131–139 (2003).
7. Miao, Y., Metzner, R. & Asano, Y. Kemp Elimination Catalyzed by Naturally Occurring Aldoxime Dehydratases. *Chembiochem : a European journal of chemical biology* **18,** 451–454 (2017).
8. Lopez, S., Fernandez-Trillo, F., Midon, P., Castedo, L. & Saa, C. First stereoselective syntheses of (-)-siphonodiol and (-)-tetrahydrosiphonodiol, bioactive polyacetylenes from marine sponges. *The Journal of organic chemistry* **70,** 6346–6352 (2005).
9. Maurer, C., Pittenauer, E., Puchberger, M., Allmaier, G. & Schubert, U. Dioximate- and Bis(salicylaldiminate)-Bridged Titanium and Zirconium Alkoxides: Structure Elucidation by Mass Spectrometry. *ChemPlusChem* **78,** 343–351 (2013).
10. Miller, S.A., Bobbitt, J.M. & Leadbeater, N.E. Oxidation of terminal diols using an oxoammonium salt: a systematic study. *Organic & biomolecular chemistry* **15,** 2817–2822 (2017).
